# Allelic variations and gene cluster modularity act as nonlinear bottlenecks for cholera emergence

**DOI:** 10.1073/pnas.2417915122

**Published:** 2025-05-28

**Authors:** Mario López-Pérez, Deepak Balasubramanian, Alicia Campos-Lopez, Cole Crist, Trudy-Ann Grant, Jose M. Haro-Moreno, Asier Zaragoza-Solas, Salvador Almagro-Moreno

**Affiliations:** ^a^Burnett School of Biomedical Sciences, College of Medicine, University of Central, Florida, Orlando, FL, 32827; ^b^Microbial Genomics and Evolution Group, División de Microbiología, Universidad Miguel Hernández, Alicante 03550, Spain; ^c^Division of Molecular Microbiology, Department of Host-Microbe Interactions, St. Jude Children’s Research Hospital, Memphis, TN 38105

**Keywords:** pathogen emergence, *Vibrio cholerae*, modularity, allelic variations, environmental reservoirs

## Abstract

The underlying factors that lead to specific strains within a species to emerge as human pathogens remain mostly enigmatic. The diarrheal disease cholera is caused by strains from a phylogenetically confined group within the *Vibrio cholerae* species, the pandemic cholera group (PCG), making it an ideal model system to tackle this puzzling phenomenon. Comprehensive analyses of over 1,840 *V. cholerae* genomes, including environmental isolates from this study, reveal that the species consists of eleven groups, with the PCG belonging to the largest and located within a lineage shared with environmental strains. This hierarchical classification provided us with a framework to unravel the ecoevolutionary dynamics of the genetic determinants associated with the emergence of toxigenic *V. cholerae*. Our analyses indicate that this phenomenon is largely dependent on the acquisition of unique modular gene clusters and allelic variations that confer a competitive advantage during intestinal colonization. We determined that certain PCG-associated alleles are essential for successful colonization whereas others provide a nonlinear competitive advantage, acting as a critical bottleneck that clarifies the isolated emergence of PCG. For instance, toxigenic strains encoding non-PCG alleles of a) *tcpF* or b) a sextuple allelic exchange mutant for genes *tcpA*, *toxT*, *VC0176*, *VC1791*, *rfbT,* and *ompU*, lose their ability to colonize the intestine. Interestingly, these alleles do not play a role in the colonization of newly established model environmental reservoirs. Our study uncovers the evolutionary roots of toxigenic *V. cholerae* offering a tractable approach for investigating the emergence of pathogenic clones within an environmental population.

## Significance

The underlying factors that lead to specific strains within a species to emerge as human pathogens remain mostly enigmatic. Toxigenic clones of the cholera agent, *Vibrio cholerae*, are encompassed within one phylogenomic clade, the pandemic cholera group (PCG). Here, we investigate the molecular and evolutionary factors that explain the confined nature of this group. Our analyses determined that the emergence of PCG is largely dependent on the acquisition of unique modular gene clusters and allelic variations that confer a competitive advantage during intestinal colonization. These allelic variations act as a critical bottleneck that elucidate the isolated emergence of PCG and provides a tractable blueprint for the study of the emergence of pathogenic clones within an environmental population.

The emergence of human pathogens is one of the most pressing public health concerns of modern times, with new outbreaks of varying magnitude occurring regularly around the world (1–4). Pathogen emergence is a complex and multifactorial phenomenon that culminates with a microorganism acquiring the ability to colonize and harm the human host, cause outbreaks, and persist within the environment (5). To date, the specific factors and evolutionary constraints that govern the emergence of bacterial pathogens from environmental populations remain mostly enigmatic (4). Elucidating how the interplay between the numerous molecular and environmental drivers lead to this complex phenomenon is critical to develop surveillance platforms to predict emergent events and potential sources of outbreaks (4). Furthermore, in addition to the profound public health implications, understanding pathogen emergence addresses one of the central questions of microbial ecology: What are the evolutionary processes within a bacterial population that underlie colonization and adaptation to a new environment? (6).

The Vibrionaceae is a highly diverse family of aquatic bacteria that encompass several species representing distinct paradigms of how pathogens can emerge from environmental populations (7, 8). Toxigenic *Vibrio cholerae* is the causative agent of the severe diarrheal disease cholera, a scourge that affects millions of people each year and is responsible for over 100,000 deaths annually (9). The first six pandemics of cholera are thought to have been caused by the Classical biotype *V. cholerae* O1, a strain that is now considered extinct (9–12). The seventh and current pandemic is caused by the El Tor biotype of *V. cholerae* O1 and started in Indonesia in 1961 (13, 14). The disease typically affects nations with limited access to clean water and poor sanitation and can be lethal if not properly treated (9, 11). Interestingly, only a limited number of *V. cholerae* strains can cause pandemic cholera (15–17). Specifically, toxigenic strains of *V. cholerae* are confined to a phylogenetic group, what we term the pandemic cholera group (PCG) (16, 18–20). Even though the mechanisms of cholera pathogenesis in humans have been extensively studied over the last several decades, the evolutionary events and molecular constraints that explain why only members from PCG can cause pandemic cholera remain unknown (21–26).

Some of the major general events that are essential for the emergence of toxigenic *V. cholerae* from environmental populations include the acquisition of the lipopolysaccharide (LPS) O1 antigen cluster and mobile genetic elements (MGEs) such as CTXΦ phage, *Vibrio* pathogenicity Island-1 (VPI-1), and *Vibrio* pathogenicity Island-2 (VPI-2) (12). Among several other differences with Classical, El Tor strains have also acquired two unique pathogenicity islands (PAIs): *Vibrio* Seventh Pandemic (VSP) I and II. CTXΦ encodes the genes for the cholera toxin, which is responsible for the severity of the profuse diarrhea associated with cholera (27). The toxin coregulated pilus (TCP), an essential colonization factor, is encoded within VPI-1 (28, 29), which also encodes several major virulence regulators such as ToxT and TcpP (29, 30). VPI-2 encodes the genes for sialic acid utilization, which confers a competitive advantage in the gut (31). Vibrio Seventh Pandemic Island I (VSP-I) codes for a regulator that plays a role in intestinal colonization and chemotaxis (32) and Vibrio Seventh Pandemic Island II (VSP-II) is associated with environmental survival and fitness of the bacterium (33–35). It was recently found that VSP-II and VPI-2 encode systems that prevent the uptake of foreign genetic material (36) These MGEs are always found in the PCG but are not exclusive to them as they are also encoded in some environmental strains of *V. cholerae* (37–39). However, to date, their abundance, specific distribution, and dynamics within the *V. cholerae* species are not well understood.

Horizontal acquisition of key virulence genes is a critical step in the emergence of toxigenic clones of *V. cholerae*, however, it is not sufficient to explain why the ability to cause cholera is limited to strains belonging to the PCG. This phylogenetically confined distribution together with the presence of CTXΦ and the PAIs in some environmental strains strongly indicate that other barriers and requirements exist that stringently limit their emergence. We recently investigated the genomic prerequisites that must be present in a population before a pathogenic clone can emerge from an environmental gene pool (19). We determined that the environmental ancestor of the PCG had a particular genomic background containing a specific set of alleles of core genes that conferred preadaptations to virulence and enhanced its pathogenic potential, what we term virulence adaptive polymorphisms (VAPs) (19). Overall, our results indicate that core genomic factors beyond the acquisition of MGEs are critical for the emergence of toxigenic *V. cholerae*. To date, the unique core genes, and allelic variations in the genomic background of toxigenic *V. cholerae* that explain their narrow distribution remain enigmatic.

In this study, we investigated the evolutionary constraints that govern the emergence of bacterial pathogens from environmental populations using toxigenic *V. cholerae* as a model system. To do this, we analyzed 1,840 *V. cholerae* genomes and found that the species is divided into eleven major groups. PCG belongs to the largest group (G3) and is located within one lineage shared with other environmental strains, the pandemic cholera lineage (PCL). Subsequently, we examined the genetic determinants associated with the emergence of toxigenic *V. cholerae* through multilevel genomic analyses. We found that these elements are made up of several modules sparsely distributed among the different phylogenomic groups and include small gene clusters and allelic variations. We determined that these allelic variations confer a competitive advantage during intestinal colonization, acting as a critical bottleneck that elucidates the isolated emergence of PCG, but do not play a role in the colonization of model environmental reservoirs. Overall, our results provide an evolutionary scenario that serves as a blueprint for the study of the emergence of facultative bacterial pathogens.

## Results and Discussion

### Hierarchical Classification and Clustering of the *V. cholerae* Species.

**Fig. 1.**
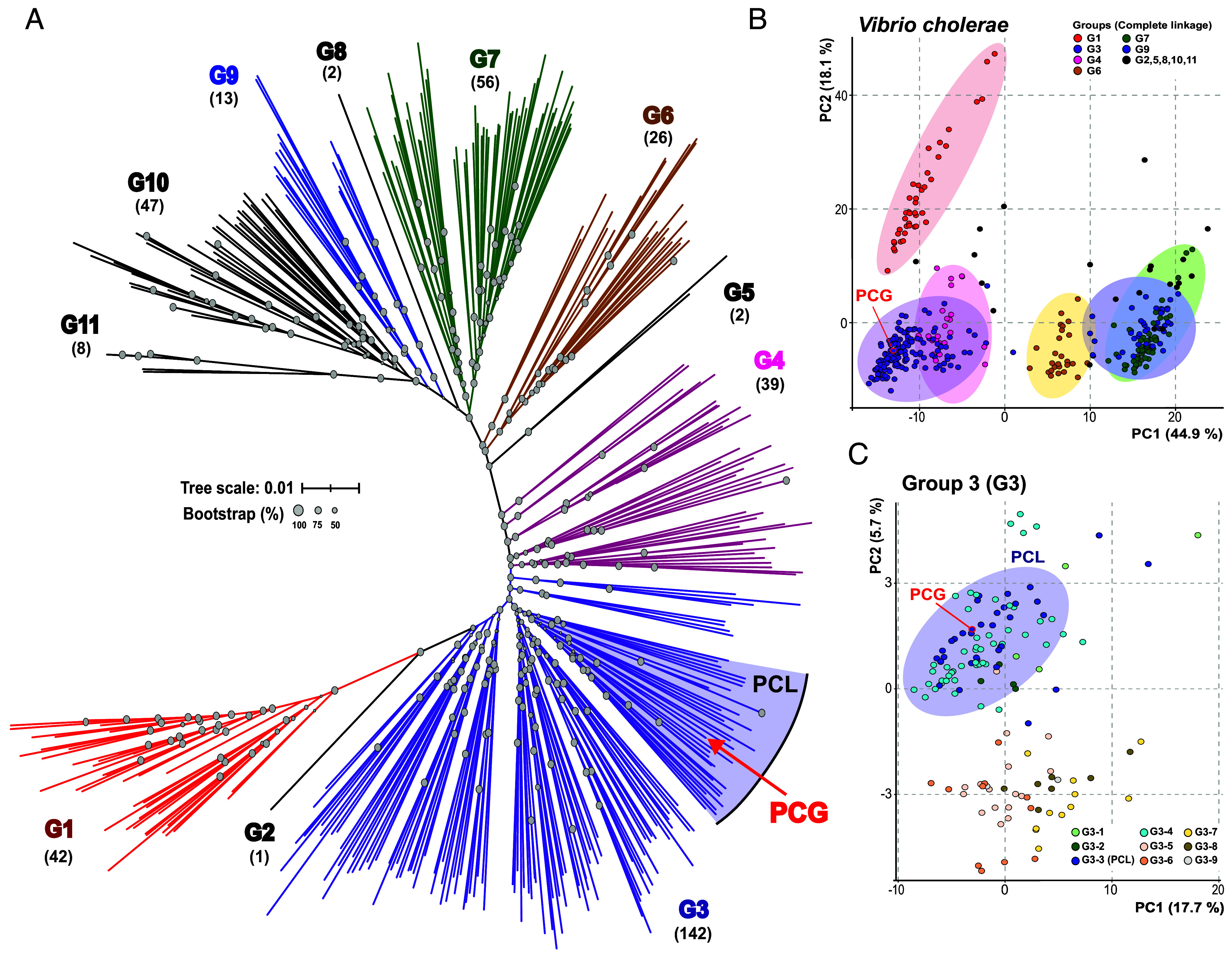
Phylogenomic and population structure of the *V. cholerae* species. (*A*) Phylogenomic tree of dereplicated *V. cholerae* strains available in public databases and strains from this study. The tree was reconstructed from single nucleotide polymorphisms (SNP) of the core genome. The branches of the members belonging to the groups have the same color. The numbers below each cluster represent the number of nonclonal clades within that cluster. The position of the PCG is marked with a red arrow. Gray circles located at the branch nodes represent the bootstrap values. (*B*) The PCA represents the clustering of nonclonal reference clades of *V. cholerae* and IRL strains based on a similarity matrix using genome-wide SNP alignment. (*C*) PCA represents the same analysis carried out for section B but only with the genomes belonging to G3, where the PCG was located.

We isolated and sequenced the genomes of 35 *V. cholerae* strains from a large estuary in eastern Florida, the Indian River Lagoon (IRL), from a diverse set of fractions (*SI Appendix*, Table S1) (40). We examined the relationship of these strains with *V. cholerae* PCG by analyzing the average nucleotide identity (ANI) in a pairwise comparison against *V. cholerae* C6706, a reference O1 strain from the El Tor biotype (*SI Appendix*, Fig. S1) (41). Interestingly, phylogenomic analysis based on whole-genome single nucleotide polymorphism (SNP) shows that the majority of the IRL strains form two major clusters, one of them including the toxigenic strain of *V. cholerae* C6706 (*SI Appendix*, Fig. S1). This prompted us to further characterize the *V. cholerae* species to investigate whether the evolution of the toxigenic strains follow a similar cluster-based pattern, as this would shed important light on the emergence and evolution of the pathogen. First, we collected the *V. cholerae* genomes available in public databases (NCBI RefSeq April 2024) together with our environmental isolates. Naturally, there is a significant bias in the databases toward *V. cholerae* O1 and O139 strains, which are mostly clonal. Therefore, in order to obtain an unbiased phylogeny, we dereplicated all these genomes to an identity of ANI > 99%. From a total of 1,840 initial genomes, we obtained 378 nonclonal reference *V. cholerae* clades (*SI Appendix*, Table S2). To maximize the consistency of our analyses, we built both a SNP-based phylogeny and genome clustering using ANI to determine the groups that constitute the *V. cholerae* species. The application of a divergence threshold of less than 97% in ANI results in a single cluster indicating that the boundary of intrapopulation sequence diversity in *V. cholerae* species is 97%. Next, we used a total of 486,015 SNPs from a core genome size of 2.34 Mb to build the species phylogeny using nonclonal reference clades and IRL strains (Fig. 1*A*). The detailed phylogenetic tree and metadata of the final genome dataset are shown in *SI Appendix*, Fig. S2 and Table S2. Subsequently, we clustered the genomes within the phylogenetic tree according to a similarity matrix using the SNP alignment. Unsupervised hierarchical clustering divided the data into eleven well-defined groups, of which five were found to be paraphyletic (G1, G3, G4, G6, and G9) (Fig. 1*B* and *SI Appendix*, Table S2). Group 3 (G3) is the largest of the groups containing 142 nonclonal clades (ca. 38% of the total) including PCG (Fig. 1*A*). The same analytical approach used to identify subgroups within the entire species was applied to G3. This clustering reveals nine subclades within G3. In one of those subclades, what we have termed the Pandemic Cholera Lineage (PCL), includes the PCG along with 33 other strains, including six IRL strains from this study (Fig. 1*C*). This provides us with a hierarchical approach based on three levels to decipher the evolutionary history of toxigenic strains of *V. cholerae* (from highest to lowest): a) the G3 within all *V. cholerae* strains, b) the PCL within G3 nonclonal clades and c) the clonal *V. cholerae* toxigenic strains within the PCL.

### Restricted Distribution of O1 Lipopolysaccharide Cluster in Environmental *V. cholerae* Strains.

**Fig. 2.**
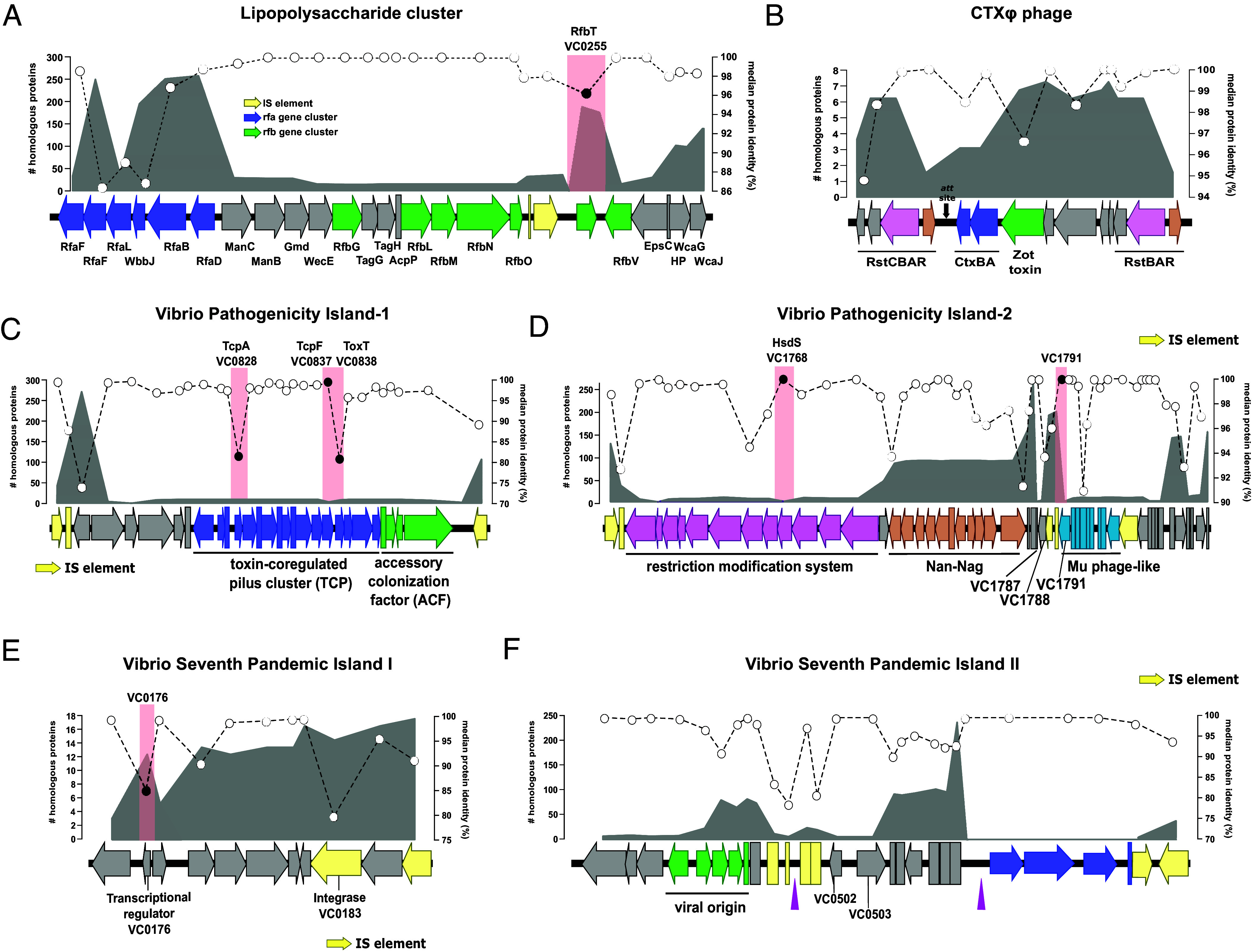
Virulence-associated genetic elements exhibit irregular distribution and modularity in *V. cholerae* species. Structures of (*A*) O1 LPS cluster, (*B*) CTXΦ phage, (*C*) VPI-1, (*D*) VPI-2, (*E*) VSP-I and (*F*) VSP-II. The area outlined in gray represents the number of homologous proteins found in the dereplicated *V. cholerae* genomes. The white circles with dotted lines indicate the median protein identity. Red rectangles on the genes indicate truncate genes or genes with high divergence that have been selected to construct isogenic mutants in which we swapped the wild-type PCG allele for an environmental allele. The black arrows in (*B*) indicate the genomic position where the cassettes have been inserted (*att* site). Pink triangles in (*F*) indicate the presence of tRNA-direct repeat genomic islands.

The LPS plays numerous roles in the pathogenesis of toxigenic *V. cholerae* (42–44) and is critical to determine the antigenic properties and classification of the bacterium (44). We examined the O1 LPS cluster to determine its distribution, evolutionary history, and allelic diversity within the *V. cholerae* species (For further information, see *SI Appendix*). The O1 gene cluster is 28 Kb long and encompasses approximately 25 ORFs with potential or demonstrated function. The *rfa* gene cluster (LPS core synthesis) is encoded at the 5′ end of the island (Fig. 2*A* blue arrows) and is widely distributed across all clades, with the notable exception of RfaF (ADP-heptose--LPS heptosyltransferase 2, VC_0235) and RfaL (O-antigen ligase, VC_0237) present in 35 nonclonal clades. Nonetheless, the genes in the *rfa* cluster exhibit the highest divergence within the LPS cluster (median identity 86 to 88%) and accumulate numerous synonymous polymorphisms similar to *Vibrio vulnificus* (45) (Fig. 2*A*). The distribution of the central region of the LPS cluster is much more restricted and is only present in 16 *V. cholerae* nonclonal clades from three groups (G1, G3, and G4); however, those that encode it share 100% identity with the region of PCG. This region contains the *rfb* cluster which is associated with antigen-O synthesis (46) (Fig. 2*A*, green arrows). Only 2 out of the 16 clades that encode this region belong to the PCL, indicating that there is no direct correlation between the phylogeny and its presence in PCG (*SI Appendix*, Table S2). The PCG encodes two transposases at the end of the *rfb* cluster, which likely increases the variability within the island and appears to be a distinguishing characteristic of the PCG strains (47, 48) (Fig. 2*A* and *SI Appendix*, Fig. S3*A*). For instance, strain N16961 encodes two additional transposases in the same region, which are inserted into the *rfb*T gene (*SI Appendix*, Fig. S3*A*). *V. cholerae* C6706 has also a truncated version of the *rfb*T gene due to a mutation that reduces the size of the gene from 861 bp to 504 bp (*SI Appendix*, Fig. S3*A*). *rfbT* encodes a methyltransferase that is responsible for seroconversion between *V. cholerae* O1 Ogawa and Inaba serotypes, suggesting a major role for these IS sequences in the emergence of PCG (47–49). Overall, we found a remarkable degree of conservation of the O1 LPS cluster, which is present in almost sixteen clades encoding a PCG-like version. It appears that the island is a) exchanged by homologous recombination leading to the whole gene cluster being conserved and b) the presence of IS elements increases the variability by truncating genes such as *rfbT*.

### Virulence-Associated Mobile Elements Exhibit Irregular Distribution and Modularity in *V. cholerae* Species.

There are several horizontally acquired MGEs that are directly associated with the emergence of toxigenic clones of *V. cholerae*. After we established the phylogeny and clustering of the *V. cholerae* species, we investigated the distribution, evolutionary dynamics, and allelic diversity of these elements within the population (For further information, see *SI Appendix*).

#### CTXΦ phage.

Inquiry into the distribution and composition of the CTXΦ phage within *V. cholerae* shows that there is no environmental strain that recapitulates all the phage genes of PCG. At most, six nonclonal clades and three groups encode 10 of the 14 genes that make up the CTXΦ phage of *V. cholerae* C6706: three from G3 (represented by strains AAS91, PivertUAT4Aug, and V060002), two from G4 (3541-04 and 3528-08), and one from G1 (458_01) (Fig. 2*B* and *SI Appendix*, Table S3). Four of these strains encode the two subunits of the cholera toxin (CtxAB). However, genes coding for the duplicated transcriptional regulator RstR (VC_1455 and VC_1464) are only found in one strain: AAS91 (Fig. 2*B*, brown shade). This strain belongs to the PCL and encodes the complete version of CTXΦ except for VC_1460 that encodes a minor coat protein pIII related to host interaction as part of the infection process (*SI Appendix*, Fig. S3*B*) (50). Instead, a transposase has been inserted in this locus in strain AAS91, followed by a APH(3’)-II family aminoglycoside O-phosphotransferase (*SI Appendix*, Fig. S3*B*). In addition, AAS91 has a third copy of the triplet (RstA-RstB-RstR) instead of the two copies present in PCG strains (*SI Appendix*, Fig. S3*B*). The second most complete version of CTXΦ in *V. cholerae* is found in strains V060002 from PCL and 3528-08 (G4). Both strains have lost the gene coding for RstC and encode a different allele of the transcriptional regulator RstR (*SI Appendix*, Fig. S3*B*).

Genomic comparison reveals that the phage variation dynamics are of the “additive” kind, where small gene cassettes are integrated into the 3′ part of the gene coding for RtxA (51). This region has previously been identified as a hotspot for genetic diversity within the PCG (16). CTXΦ variants have conserved intergenic regions that separate and mark the insertion of these gene cassettes (*att* sites, marked as black arrows). Some strains such as 3528-08 (G4) can have up to five insertions (*SI Appendix*, Fig. S3*C*). The presence/absence of these cassettes is not correlated with the phylogeny and, when present, they have high identity indicating a fast turnover of these elements (*SI Appendix*, Fig. S3*B*). In addition, using the sequence of *att* sites as reference we also found a collection of cassettes in the same order (including the one containing the two subunits of CT) in the close relative species *Vibrio mimicus*, suggesting another potential donor of CTXΦ to nontoxigenic *V. cholerae* (*SI Appendix*, Fig. S3*D*).

#### VPI-1.

The variable region of VPI-1 is located between the CDS VC_0821 (hypothetical protein) and VC_0846 (integrase) (Fig. 2*C*). The most conserved version of this region can be found in eleven clades, five of them within G3 (2 from PCL), five from G4, and one from the G7 (Fig. 2*C* and *SI Appendix*, Fig. S3*E*), including strain IRLE0081 isolated in this study. The average identity of the proteins was highly conserved compared to PCG (ca. >97%), however, we found some notable exceptions. First, among the proteins that are part of the TCP operon, we found high divergence rates in the master regulator ToxT (VC_0838) and the toxin-coregulated pilus major pilin, TcpA (VC_0828) with a median protein identity ca. 81% (Fig. 2*C*). In addition, we found that the PCG allele of the colonization factor TcpF (VC_0837) was only present in three strains (one PCL and two G3). In the rest, despite the conservation of synteny and similarity of the whole TCP operon, the identity of this protein was less than 40%, encoding a different allele of this poorly understood secreted protein (52–54) (*SI Appendix*, Fig. S3*E*). Finally, our genomic comparisons reveal that the gene coding for ToxR-activated gene A, TagA (VC_0820) was absent from the G4 genomes, while being retained in three of the five G3 genomes (*SI Appendix*, Fig. S3*E*). Overall, despite widespread conservation in the synteny and size of the clusters of genes that comprise VPI-1 in the *V. cholerae* species, the existence of unique major allelic variations in a limited number of genes might act as a bottleneck in the emergence of PCG.

#### VPI-2.

Unlike other MGEs, the differential abundance of the gene clusters within VPI-2 suggests that the island is made up of different modules (17, 31, 36, 55). Also, all but one of the IS elements of the island are widespread among several clades in diverse locations, which likely favors the transfer of the different modules. The canonical VPI-2 from O1 strains encodes three major modules, from 5′ to 3′: a) a restriction modification system (RM) (M1), b) the Nan-Nag cluster (M2), and c) a Mu-phage like region (M3) (Fig. 2*D*). At the 3′ end of VPI-2 there is also a small group of genes encoding hypothetical proteins. M2 is present in 115 nonclonal clades, whereas the distribution of M1 is more limited, being encoded by only five clades (Fig. 2*D* and *SI Appendix*, Table S2). M3 is present in twelve clades, however, there is no relationship between specific modules and species phylogeny. Like VPI-1, no clade other than PCG encode a PCG-like version of VPI-2. Furthermore, strains that contain the three individual modules sometimes encode them in different locations within the genome. For instance, strain PivertUAT4Aug (G3), the only one with the three major modules, encodes M1 and M2 within a version of VPI-2, whereas M3 is encoded in a different location of the genome (*SI Appendix*, Fig. S3*F*). In M3, the Mu-phage like module, VC_1788 and VC_1791 comprise two parts of a gene coding for the tape measure protein that have been truncated by the insertion of two transposases in the PCG, annotated as VC_1789 and VC_1790 (Fig. 2*D*). Although the synteny in the modules is conserved, and with high similarity (*SI Appendix*, Fig. S3*F*), we found a high divergence in the VC_1768 gene coding for a restriction endonuclease S subunit (HsdS) between PivertUAT4Aug and the PCG-like version of VPI-2 with a protein identity of 64% (*SI Appendix*, Fig. S4*D*). Another instance of this modular distribution is exemplified by strain 17-VB00206 (G1), which possesses the first two modules but not M3. Furthermore, the strain has lost the gene encoding the HsdS protein from M1 (*SI Appendix*, Fig. S3*F*). Overall, VPI-2 in PCG must be understood based on a) its unique modular arrangement not by the individual units that constitute it and b) the possibility that allelic variability in *hsdS* and loss of function of the VC_1791 gene play a unique role in the pandemic group. Its modules are associated with IS elements that are widely distributed among *V. cholerae* environmental strains and scattered throughout the genome. This leads to the convergence of all modules in the same island and genome being extremely low, which contributes to the rarity of PCG.

#### VSP-I.

The role of VSP-I in *V. cholerae* pathogenesis is not as clearly defined as other MGEs. VSP-I has been suggested to play an environmental role related to chemotaxis (56) and encodes a regulator involved in intestinal colonization (VC_0177) (32). We identified homologs of the eleven genes that comprise this island in eleven *V. cholerae* nonclonal clades three of them belonging to IRL strains (IRLE0062, IRLE0049, and IRLE0077) located within the same cluster (G9) (Fig. 2*E* and *SI Appendix*, Table S2). The greatest sequence divergence corresponds to its integrase (VC_0183) and an XRE family transcriptional regulator (VC_0176) encoded within the island (Fig. 2*E* and *SI Appendix*, Fig. S4*E*). Only two strains encode a PCG-like VSP-I: V060002 (G3), and VcCHNf4 (G6). Surprisingly, V060002 encodes two identical copies of VSP-I in close proximity to each other with the LPS cluster located between them (*SI Appendix*, Fig. S3*G*). This duplication of VSP-1 has also been previously reported in three toxigenic isolates of *V. cholerae* O139, where the additional copy was inserted into the smaller chromosome (57). The landscape of evolutionary possibilities such as modularity is much more limited for VSP-I than for the other MGEs due to its small size. It is also the rarest of all the islands with one transcriptional regulator, VC_0176, exhibiting high allelic diversity and very limited distribution. The rarity of the island and diversity of the regulator suggests that this is a critical bottleneck in the emergence of the Seventh Pandemic El Tor strains.

#### VSP-II.

The presence of VSP-II in clinical and environmental strains might be associated with environmental survival and fitness of the bacterium (33–35). Our analyses indicate that, similar to VPI-2, VSP-II is highly modular with several clusters of genes possessing a tRNA-Met insertion point. The direct repetition of the end of the tRNA (highlighted as purple triangles) marks the insertion points of each module, which in most cases matches the presence of IS elements (Fig. 2*F* and *SI Appendix*, Fig. S3*H*). Overall, this island serves as another example of an “additive” island with three small modules. Recent reports support our scenario that RNA-Met appear to act as a hotspot for phage-associated defense systems (58). Module 1 comprises genes VC_0490 to VC_0498, five of which are of viral origin but lack essential proteins for packaging. Module 2 encompasses VC_0502 to VC_0510, however, the lack of possible annotation of its genes precludes from assigning a specific function to this module. Nonetheless, VC_0502 and VC_0503 from this module are only present in strains from G3 (Fig. 2*F* and *SI Appendix*, Table S2). While islands are mostly conserved between *V. cholerae* PCG strains, Module 3 of VSP-II exhibits a distinct sequence. Strains from a Peruvian outbreak, such as C6706, have replaced the five genes (VC_0511 to VC_0515) with a completely different cluster of four genes (59). This variant is extremely rare and only encoded by two strains from G1 (458_01 and R18275) (*SI Appendix*, Table S2). The VSP-II Peruvian variant is illustrated in Fig. 2*F* whereas the one encoded by other PCG strains is depicted in *SI Appendix*, Fig. S3*H*. Leaving aside Module 3, the closest versions are encoded by strains N2723, MZO-3, and V060002 belonging to G3, which possess similar modularity and synteny of VSP-II from PCG with some gene losses and transposase acquisitions (*SI Appendix*, Fig. S3*H*). In the rest of the species, VSP-II only maintains some of these modules or clusters of genes, with the addition of other types of clusters being common. Finally, our analyses did not reveal any gene that exhibits high rates of divergence within the island, suggesting allelic diversity might not act as a major constraint in this element.

### Allelic Variations and Modularity Are Broadly Conserved in the PCG.

**Fig. 3.**
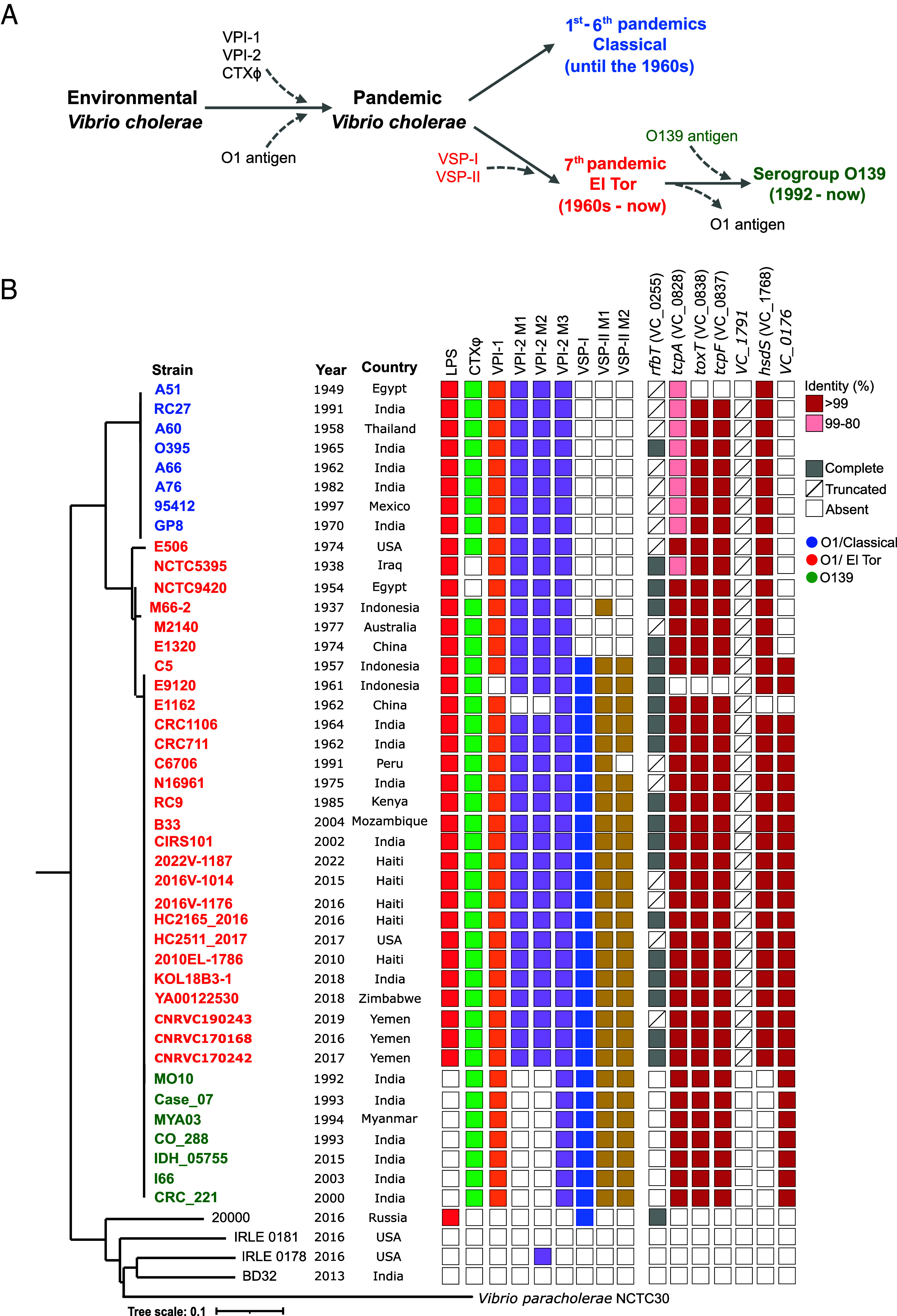
Allelic variations and modularity are broadly conserved in the PCG. (*A*) Schematic of steps involved in the acquisition of main virulence-associated genetic elements by environmental strains of *V. cholerae* to gain pandemic potential. VPI-1, *Vibrio* pathogenicity island-1; VPI-2, *Vibrio* pathogenicity island-2; CTXΦ, CTX phage; VSP-I, *Vibrio* seventh pandemic island-I; VSP-II, *Vibrio* seventh pandemic island-II. (*B*) The phylogenomic tree encompasses 42 strains of serogroups O1 and O139, in addition to the two biotypes within the O1 strains (e.g., “classical” and “El Tor”). These strains are responsible for cholera and encompass strains from prepandemic to contemporary strains. The tree was constructed using single nucleotide polymorphisms of the core genome. Non-PCG environmental isolates and *V. paracholerae* strains were used as outgroup. Strains are colored according to their origin (green O139, blue O1 classical, and red O1 El Tor) as well as the presence or absence of each genetic element and allele.

Despite belonging to the same clonal frame (e.g., ANI > 99%), different stages have occurred within the PCG leading up to the seventh and current pandemic strain (13, 60–62) (Fig. 3*A*). To determine the evolutionary history of the genetic elements analyzed above within the PCG, we conducted an analysis of the presence/absence of each element using a comprehensive curated selection of strains encompassing both serogroups O1 and O139, as well as the two biotypes within the O1 strains: classical and El Tor (Fig. 3*B*). First, we constructed a phylogenomic tree of 42 strains that encompass from the oldest genome available of the PCG to the most recent outbreaks, such as those in Haiti or Yemen (63, 64). Our selection spans a range of approximately 80 y and incorporates strains both prepandemic and from the seventh pandemic (Fig. 3*B*). Environmental isolates and *Vibrio paracholerae* strains were used as outgroups (Fig. 3*B*) (65, 66). As expected, VSP islands are not present in classical isolates, and the O139 isolates possess a different LPS island. The modules 1 and 2 of VPI-2 are also absent in O139 isolates. The early prepandemic strains of the El Tor biotype, NCTC8457 and NCTC5395, do not encode the CTXΦ phage or the islands associated with the seventh pandemic (VSP-I and VSP-II), which appear complete in strain C5 (1957) (63) (Fig. 3*B*). Nonetheless, all strains from 1970s onward possess the complete set of genetic elements with the only exception, as mentioned above, being strains from the Peruvian outbreak, where module 2 of VSP-II is absent (Fig. 3*B*). Overall, besides specific exceptions, modularity and gene content in those elements is broadly conserved in the PCG.

As highlighted above, we identified a set of genes encoded within those elements that are dispersed in the phylogeny of the species and possess unique variations in the PCG that show high divergence in relation to the rest of *V. cholerae* strains (Fig. 2). Specifically, those genes encode: 1) RfbT (VC_0255), which belongs to a FkbM family methyltransferase located in the LPS cluster and is associated with Inaba-to-Ogawa serotype conversion (49). 2) The main pilin subunit of the TCP, TcpA (VC_0828), which is encoded in VPI-1 (29, 67). 3) The master virulence regulator ToxT (VC_0838), also encoded in VPI-1 (22, 29, 68). 4) The secreted factor of unknown function TcpF (VC_0837) encoded within VPI-1 that is essential for intestinal colonization (52, 53). 5) The tape measure protein VC_1791 in the Mu phage-like module encoded within VPI-2 (31). 6) The restriction endonuclease S subunit encoded in VPI-2 HsdS (VC_1768) (31). 7) VC_0176, a transcriptional regulator of the XRE family encoded within the VSP-I island (17). Subsequently, we investigated the distribution and similarity of these alleles within the PCG. Within the classical strains, the *rfbT* gene is truncated only in O395 whereas it is truncated in 70% of the El Tor strains (in 19 out of 27) (Fig. 3*B*). Similar to the *rfbT* gene, VC_1791 is also truncated, however, this truncated version is present in all genomes except the classical A51 strain. The other five genes, except for *tcpA*, possess alleles in the PCG that share >99% identity with the one encoded by C6706 (Fig. 3*B*). The *tcpA* allele of the classical biotype shares 83% identity to the El Tor allele, while the rest of the El Tor alleles are identical (Fig. 3*B*). Our results indicate that these distinct allelic variations are largely conserved in the PCG.

### Limited Allelic Diversity within PCG-Associated Genes.

**Fig. 4.**
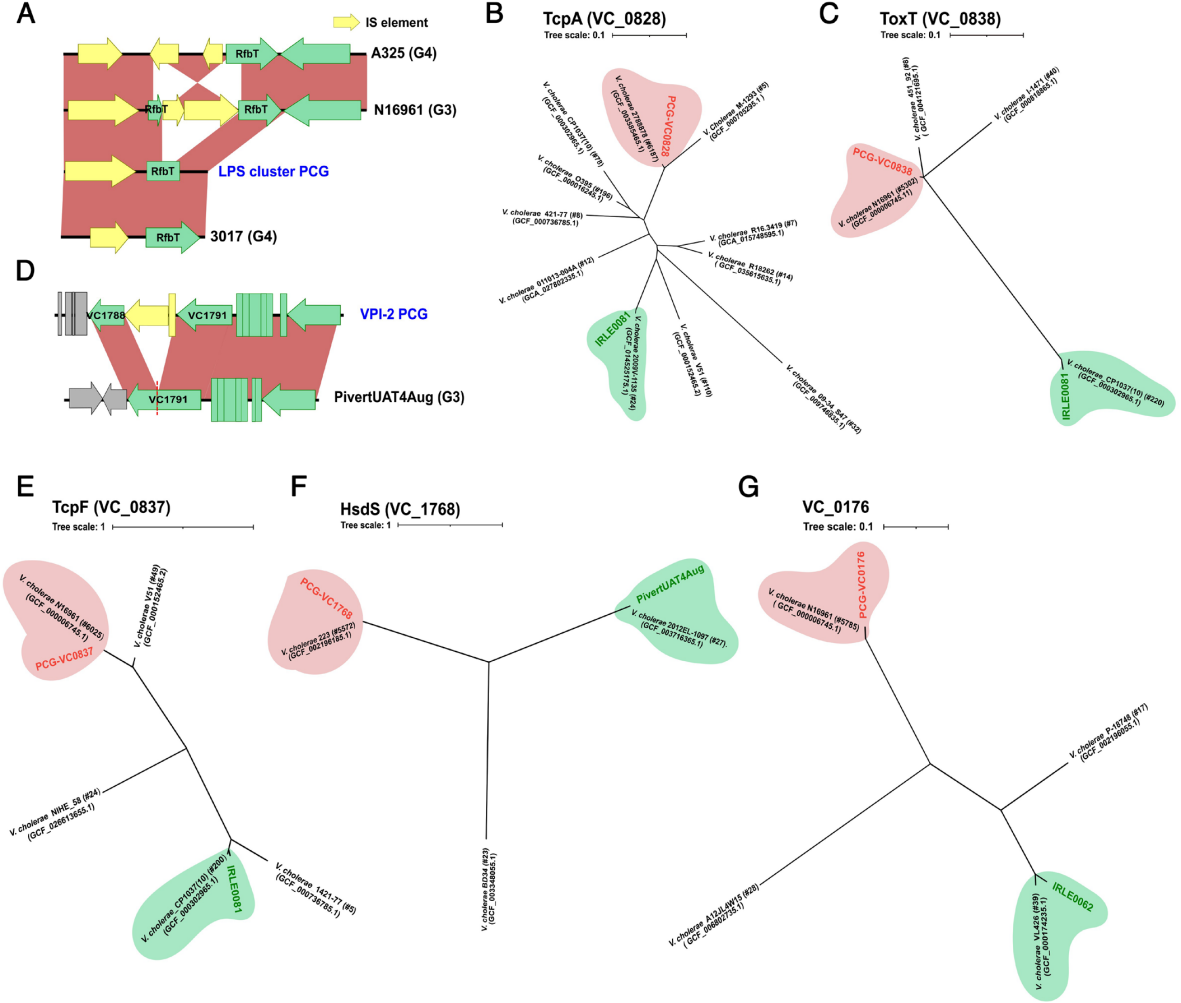
Limited allelic diversity within PCG-associated genes. (*A*) Schematic representation of the variable zone of the LPS containing the different variations of the *rfb*T gene. Maximum likelihood phylogenetic tree of (*B*) *tcp*A (VC_0828) and (*C*) *tox*T (VC_0838). (*D*) Schematic representation of the IS elements inserted in the PCG VC_1791 allele. IS elements are highlighted in yellow. Maximum likelihood phylogenetic tree of (*E*) *tcp*F (VC_0837), (*F*) *hsd*S (VC_1768) and (*G*) VC_0176 gene diversity in the *V. cholerae* species. The numbers next to the tags indicate the number of sequences clustered at 99%. The location of the PCG allele is indicated in red, while the environmental allele selected for allelic exchange is highlighted in green.

The set of alleles encoded within the elements described above are conserved within the PCG and show high divergence in relation to other *V. cholerae* strains (Figs. 2 and 3*B*). We hypothesized that they might act as bottlenecks for the emergence of toxigenic clones of *V. cholerae* and shed light on the evolution of PCG. In order to initially test this, first we retrieved all the genetic diversity within the species for each allelic variant from the GenBank database (ca. 6000 sequences per allele) and performed phylogenetic analyses to determine the true allelic diversity of those genes (Fig. 4). Despite analyzing all available genome and gene sequences in the *V. cholerae* species, we found pervasive clonality and very limited diversity. Our analyses helped us select potential environmental variants that show both a) high distance from the wild-type PCG allele and b) are abundant in the environmental population (Fig. 4). Based on these analyses, we constructed isogenic mutants where we exchanged the PCG allele (Fig. 4 red circles) for one of the environmental ones that met those criteria (Fig. 4 green circles) (*SI Appendix*, Table S4). The C6706 allele of *rfbT* has a shorter version of the gene due to a mutation that inserts a stop codon (Fig. 4*A*). In the isogenic mutant, the truncated gene was replaced by the full version of the gene found in *V. cholerae* A325 (Fig. 4*A*). For allelic exchange of *tcpA,* since it is the only gene that encodes several alleles, we created individual isogenic mutants in the background of *V. cholerae* C6706 swapping the PCG allele for the one encoded by *V. cholerae* isolate IRLE0081, which has a protein identity of 80% and meets both criteria described above (Fig. 4*B* and *SI Appendix*, Fig. S4*A*). The topology of the phylogenetic tree of the master virulence regulator ToxT shows two distinct branches (Fig. 4*C*). We also selected the allele encoded by *V. cholerae* isolate IRLE0081, which shows 79% identity compared with the PCG variant (Fig. 4*C* and *SI Appendix*, Fig. S4*B*). The PCG allele of VC_1791 is truncated by the insertion of two transposases and was replaced by the allele of *V. cholerae* strain PivertUAT4Aug, which has the complete version of the gene (Fig. 4*D*). The phylogeny of the secreted factor TcpF shows the highest divergence within the group. For this factor we generated two mutants: a) one where we exchanged the PCG allele for that encoded by the *V. cholerae* isolate IRL0081, which clustered with 200 other sequences and shows 33% identity (*SI Appendix*, Fig. S4*C*), and b) an allele within a third branch represented by the *V. cholerae* strain NIHE_58 with 31.9% identity (Fig. 4*E*). The identity between the two non-PCG alleles is 31.7%, indicating the presence of three paralogs within TcpF. According to its phylogeny, *hsdS* has three major alleles, however, their distances are not as marked as that of *tcpF* (Fig. 4*F*). We exchanged the PCG *hsdS* allele for the one encoded by the *V. cholerae* strain PivertUAT4Aug, which has a protein identity of 64% (*SI Appendix*, Fig. S4*D*). Similarly, based on the phylogeny, VC_0176 has a limited number of alleles with the most distant and abundant being the one encoded by *V. cholerae* IRL0062 (85% identity), which we selected to construct isogenic mutants (Fig. 4*G* and *SI Appendix*, Fig. S4*E*). Overall, our data indicate that, with the exception of *tcpA*, there is limited allelic diversity but marked divergences within the identified PCG-associated genes, strengthening the possibility of them playing a role in the emergence of pandemic cholera.

### PCG-Associated Allelic Variations Confer a Competitive Advantage during Intestinal Colonization.

**Fig. 5.**
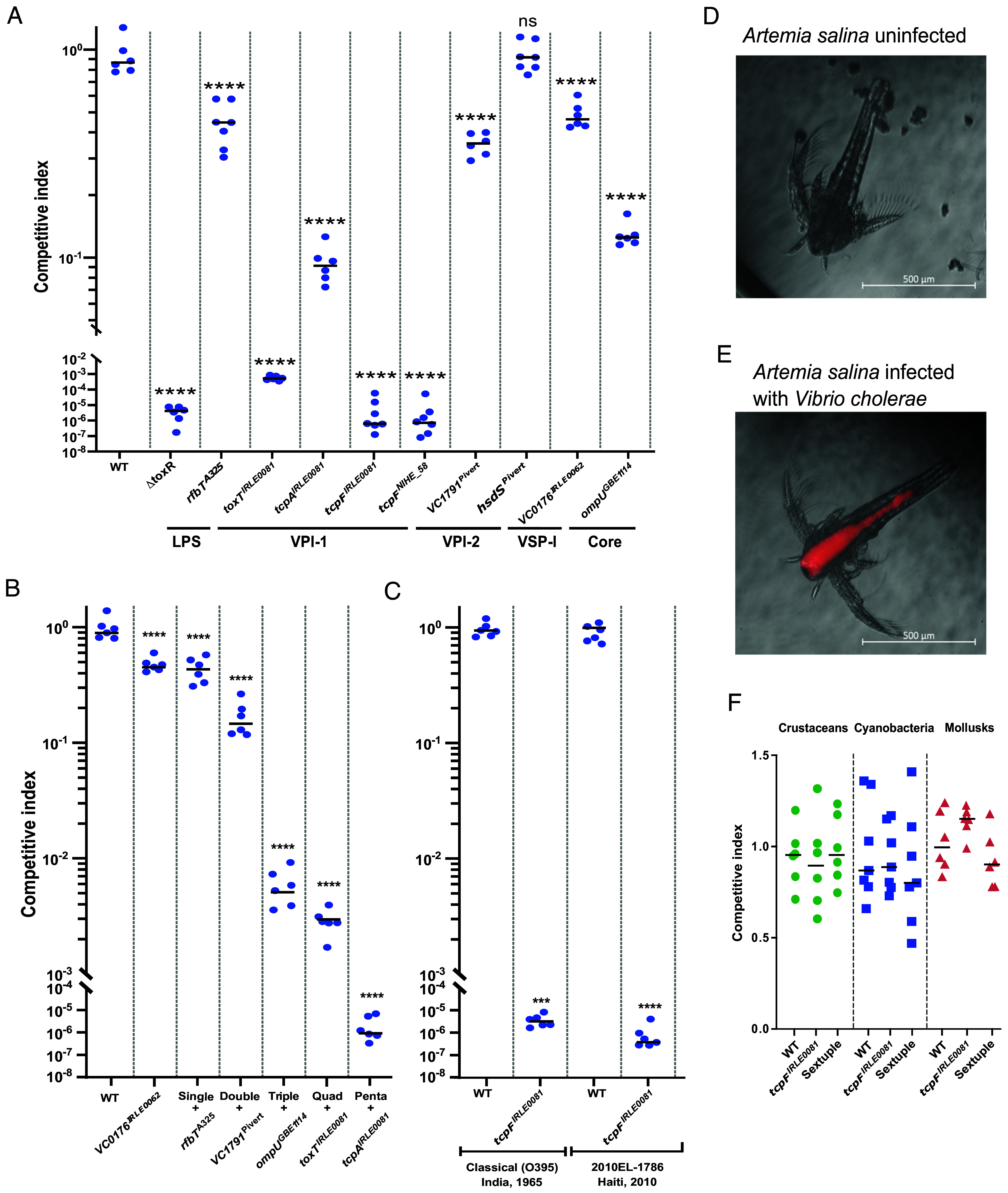
Allelic variations act as fitness bottleneck during intestinal colonization. Isogenic *V. cholerae* mutant strains were orogastrically inoculated in 3- to 5-d-old CD-1 mice to perform competition assays. Strains were monitored for colonization of the small intestine. The relative colonization data are represented as competitive indices (CI) in comparison to a coinfected *V. cholerae* C6706 ∆*lacZ* strain or a ∆*lacZ* mutant constructed in background of the indicated strain (*V. cholerae* O395 or 2010EL-1786). (*A*) Isogenic strains encoding individual non-PCG alleles exhibit different CI during intestinal colonization. (*B*) Combination mutants of the non-PCG alleles demonstrate an additive decrease in colonization efficiency. (*C*) Allelic variations in *tcpF* show conserved role during intestinal colonization of different *V. cholerae* PCG isolates: classical O395 and El Tor 2010EL-1786, n = 6 per strain. *P*-values indicate significance compared to the WT strain, unless otherwise specified, and are calculated by two-way ANOVA in Šidák’s multiple comparisons test; *** <0.0001, **** < 0.00001. (*D*) Image of *A. salina* uninfected with *V. cholerae*. (*E*) RFP-tagged *V. cholerae* C6706 cells are ingested by *A. salina* colonizing and proliferating to fill the gut space. (*F*) CI of *tcpF*^IRL0081^ and the sextuple mutant in three model environmental reservoirs: Crustaceans (*A. salina*), cyanobacteria (*M. aeruginosa*), and mollusks (gill homogenates of the Eastern oyster, *C. virginica*). The mutants do not exhibit any significant differences in CI during colonization of these reservoirs, n = 6. *P*-values indicate significance compared to the WT strain and are calculated by two-way ANOVA.

Subsequently, we performed competitive assays using the infant mouse model of infection to determine whether the PCG-associated allelic variations of the genes above confer a fitness advantage to toxigenic *V. cholerae*. Our results indicate that the PCG allele of *tcpF* is essential during the infectious process, with the mutant strain *tcpF^IRLE0081^*exhibiting total loss of intestinal colonization, like the ∆*toxR* strain used as negative control, which is avirulent (Fig. 5*A*). To further examine the potential essentiality of the PCG allele of TcpF, we tested the strain encoding the other *tcpF* paralog, *tcpF^NIHE_58^*(Figs. 4*E* and 5*A*). Our results indicate that *tcpF^NIHE_58^*cannot successfully colonize the intestine either (Fig. 5*A*). On the other hand, the allelic specificity of HsdS does not appear to play a direct role in the emergence of PCG as the competitive index (CI) of the *hsdS^PIVERT^* mutant is close to 1 (Fig. 5*A*). The mutant strains for the other 5 genes exhibit a varying degree of fitness loss during intestinal colonization with *tcpA^IRLE0081^* showing the largest decrease (~3-logs), followed by *toxT^IRLE0081^* (~1-log), *VC1791^PIVERT^* (~1-log), *rfbT^A325^* (~sevenfold), and *VC0176^IRLE0062^* (~fivefold) (Fig. 5*A*).

### Allelic Variations Act as Nonlinear Fitness Bottleneck during Intestinal Colonization.

We recently determined that allelic variations in core genes, in the form of virulence adaptive polymorphisms (VAPs), confer critical preadaptations for the emergence of pathogenic traits (19). For instance, toxigenic strains encoding environmental alleles of the major outer membrane porin OmpU from the strain exhibit bile sensitivity and reduced resistance to host antimicrobials (19, 69). Furthermore, those strains (e.g., *ompU^GBE1114^*) show a colonization defect in the infant mouse model (~1-log) (Fig. 5*A*) (19). To unveil the potential relationship among these allelic variations (MGEs and core genome), we constructed a set of mutant strains combining these alleles (Fig. 5*B* and *SI Appendix*, Table S4). We excluded those that showed no defect in colonization (*hsdS^PIVERT^*) or led to complete loss of colonization (*tcpF^IRLE0081^* or *tcpF^NIHE_58^*). We constructed the mutants on the background of *VC0176^IRLE0062^*, as it was the one that showed the lowest effect in the CI (Fig. 5*A*) and exchanged the alleles in ascending order based on their effect on the CI. Specifically, first we constructed a double mutant by introducing *rfbT^A325^*that exhibits a ~fivefold decrease in its CI. Next, in the double mutant background, we constructed a triple mutant by introducing *VC1791^PIVERT^* (~ninefold). Subsequently, we constructed a quadruple mutant by introducing *ompU^GBE1114^* (~2.2-log) and a quintuple by introducing *toxT^IRL0081^*in the previous background (~2.9-log). Finally, we generated a sextuple mutant encoding the previous five environmental alleles and *tcpA^IRLE0081^* which is fully defective for intestinal colonization. Overall, there is a variable and nonlinear effect between the alleles, with the largest effect being that of the introduction of *ompU^GBE1114^* and *tcpA^IRLE0081^* (Fig. 5*B*). Phenotypic analyses indicate that the complete loss of colonization exhibited by *tcpF*^IRLE0081^ and the sextuple mutant is not due to a growth defect, loss of motility, or changes in their ability to form biofilm (*SI Appendix*, Fig. S5). Our results demonstrate that allelic variations in MGEs, LPS cluster, and core genome act as an additive yet stringent bottleneck that explicates the confined nature of toxigenic *V. cholerae* isolates and the PCG.

### Allelic Variations Show Conserved Role among *V. cholerae* PCG Isolates.

Next, we examined whether our findings in *V. cholerae* C6706 could be extrapolated to other PCG strains. To determine the potentially conserved function of these alleles, we constructed mutants of *tcpF* in two backgrounds (*SI Appendix*, Table S4), since that gene showed the most marked phenotype and is essential for colonization in C6706. We exchanged the non-PCG *tcpF* allele *tcpF^IRLE0081^* in the background of *V. cholerae* O395, a classical strain from 1965 that belongs to the biotype that caused the first six pandemics, and *V. cholerae* O1 El Tor 2010EL-1786, a Haitian strain from the ongoing Haitian outbreak (63, 64). As shown in Fig. 5*C*, similar to *V. cholerae* C6706, the *tcpF* mutant in neither of the two backgrounds can successfully colonize the intestine, corroborating the role of these alleles in the emergence of *V. cholerae* PCG. Our findings further strengthen a scenario where specific PCG-associated alleles are essential for the emergence of toxigenic *V. cholerae*.

### PCG Alleles Do Not Influence Colonization of Model Environmental Reservoirs.

Colonization of new niches or hosts is often associated with fitness trade-offs that make those adaptations beneficial within one context while detrimental in another (70, 71). To determine potential environmental fitness trade-offs associated with the PCG alleles, we examined the colonization dynamics of the two isogenic mutant strains that exhibit complete loss of intestinal colonization, *tcpF*^IRL0081^ and the sextuple, during colonization of three model environmental reservoirs: crustaceans, cyanobacteria, and mollusks.

a) Crustaceans. We developed a model of infection using the brine shrimp *Artemia salina* to examine the colonization of crustaceans by *V. cholerae* and conduct competition assays, analogous to intestinal colonization studies in mice (Fig. 5 *D* and *E* and *SI Appendix*, Fig. S6). First, we examined the colonization dynamics of *V. cholerae* C6706 at different concentrations and time points (*SI Appendix*, Fig. S6*A*). We determined that infection with 10^7^ CFU/mL for 48 h leads to consistent colonization of *A. salina* nauplii (*SI Appendix*, Fig. S6). Surprisingly, even though we expected *V. cholerae* to colonize the chitinaceous surface of *A. salina*, the bacterium primarily colonizes the gut space of the nauplii (Fig. 5*E*). Next, we performed coinfection studies using a similar approach as those in the infant mouse. Our competition assays demonstrate that mutant strains encoding *tcpF*^IRL0081^ or the sextuple mutant colonize *A. salina* at similar levels as the WT strain with a CI ~1 (Fig. 5*F*).
b) Cyanobacteria. We used *Microcystis aeruginosa* as a model system to investigate the interactions of *V. cholerae* with cyanobacteria as the bacterium has been found associated with this species between cholera outbreaks in endemic areas (72, 73). Competition assays demonstrate that, like *A. salina*, both mutants colonize the mucilaginous sheath of *M. aeruginosa* comparably to the WT strain (Fig. 5*F*).
c) Mollusks. To examine the interaction of *V. cholerae* with mollusks, we extracted the mucus-containing gills of Eastern oysters (*Crassostrea virginica*) and performed competition assays using their homogenates (*Materials and Methods*). Similarly, the sextuple mutant and *tcpF*^IRL0081^ exhibit a CI in oyster gill homogenates analogous to the WT toxigenic *V. cholerae* isolates (Fig. 5*F*). Overall, our results indicate that no significant fitness advantage or trade-off is associated with the acquisition of PCG alleles in these model systems.

## Conclusions

**Fig. 6.**
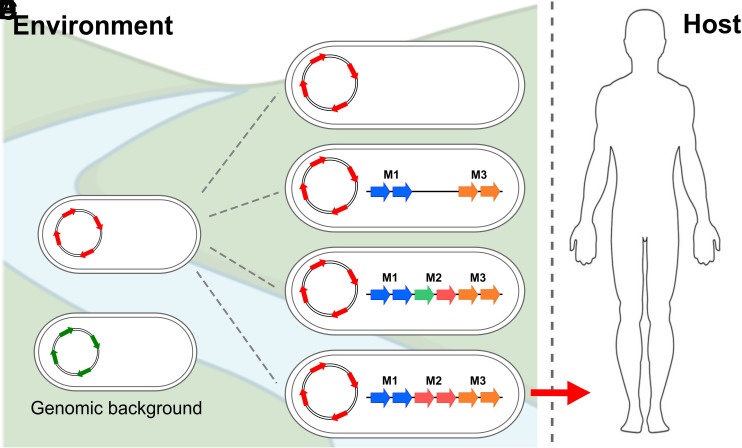
Model of emergence of the pandemic cholera group in *V. cholerae*. The evolutionary history of PCG suggests that the emergence of this group has not been a linear process and contains a series of major bottlenecks that explains its rarity. (*A*) A specific genomic background containing preadaptations to virulence in the core genome (red arrows) is essential for the emergence of virulence traits in *V. cholerae*. Green arrows: Strains not encoding these preadaptations. (*B*) The acquisition of certain gene clusters is critical (e.g., CTXΦ, VPI-1, etc.) together with (*C*) the presence of specific modules within these gene clusters and (*D*) certain unique allelic variations within them. (*E*) The correct combination of all the elements of this complex mosaic provides the foundation for the emergence of pathogenic clones within an environmental population of *V. cholerae* (red arrow).

In this study, we took a systematic approach toward unraveling the evolutionary history of the enigmatic PCG, which, to date, remains the only clade within the *V. cholerae* species capable of causing epidemics and pandemics of the severe diarrheal disease cholera. Phylogenomic analyses of the species reveal a cluster-based evolution of *V. cholerae* with several hierarchical levels leading to the emergence of the PCG. Our investigations on the abundance and distribution of the virulence-associated genes and MGEs suggest the existence of several layers in the evolutionary dynamics and emergence of *V. cholerae* (Fig. 6).

Our analyses indicate that a) strains from *V. cholerae* PCG have acquired the gene clusters encoding the major virulence genes in a modular fashion, b) those MGEs are more widespread than previously thought within the *V. cholerae* species, c) a select set of highly divergent genes dispersed in the phylogeny of the species encode unique allelic variations in PCG that act as stringent bottlenecks for emergence. Pangenome analyses of the *V. cholerae* species and historical reconstruction of the evolution of PCG strains reveal that, besides the acquisition and modularity of the main MGEs, the absence or presence of other genes does not appear to play a major role in the emergence and evolution of the pandemic group (*SI Appendix*, Fig. S7). MGEs are additive genomic islands composed of several modules bordered by IS elements. These modules have a rapid turnover and remain associated with each clade for a short time. In addition, these modules have the potential to be inserted into other regions of the genome. This mosaic-like degree of molecular sophistication explains the absence of a complete set of PCG-like elements in any other *V. cholerae* clade. Indicating that, in order to analyze the evolutionary history of these mobile elements, it is necessary to examine the specific modules and individual alleles of some genes within them rather than investigating presence or absence of the elements (Fig. 6).

To examine whether these intramodular allelic variations act as an evolutionary constraint that govern the emergence of toxigenic *V. cholerae*, we constructed isogenic mutant strains where we exchanged the PCG-associated allele for an environmental one and investigated their fitness during intestinal colonization. We determined that the PCG-associated alleles lead to a competitive advantage during intestinal colonization. Surprisingly, we uncovered a differential effect between alleles of genes encoded within MGEs, the LPS, and core genome. Specifically, we found that some PCG-associated alleles are essential for successful colonization, such as *tcpF*, whereas others provide nonlinear competitive advantages during infection that ultimately act as a critical bottleneck that clarifies the isolated emergence of PCG. The infant mouse model system has been critical in identifying essential virulence factors for cholera in humans and was also used in this study to examine the bottlenecks for colonization associated with the emergence of the PCG (24, 28, 74, 75). Future research will clarify the specific roles, relative importance, and potential interactions of these alleles in shaping human infection dynamics and disease dissemination across endemic and nonendemic regions. Elucidating this will open novel research avenues and provide essential insights into the factors and network of genes associated with the emergence of virulence traits in bacteria. Finally, it appears that none of the three environmental reservoirs we examined in this study exert the ecological pressures associated with the selection of the PCG alleles as the mutants exhibit a similar CI as the wild-type strain. Given that these variations originate from the environment it is possible that a combination of abiotic and biotic factors might select for the PCG alleles in the aquatic ecosystem. Uncovering the environmental drivers fostering the selection of factors associated with pathogen emergence will provide critical insights toward the development of surveillance networks. Overall, our investigations provide unique insights and perspectives to elucidate the barriers associated with the emergence of human pathogens and can be extrapolated to the study of niche colonization and other complex phenomena.

## Materials and Methods

### Strains and Growth Conditions.

For further information on materials and methods see Supplementary text. *V. cholerae* strains were routinely grown on Luria Bertani (LB) agar at 37 °C for approximately 16 h, unless otherwise stated. For routine liquid cultures, isolated colonies selected from agar plates were grown aerobically at 37 °C in LB broth for ~16 h. Tryptone broth (tryptone 10 g/L, NaCl 5 g/L), artificial sea water, and M9 minimal media (Fisher) supplemented with 0.1% glycerol were used for biofilm formation, studies using *Artemia* and oyster gill homogenates, respectively. Growth medium was supplemented with the following antibiotics and reagents, as needed: polymyxin B 50 U/mL, kanamycin 45 µg/mL, streptomycin 1,000 µg/mL, gentamycin 15 µg/mL, X-gal 40 µg/mL. Isolation of *V. cholerae* from water samples was performed using a modified protocol from Huq et al. (76). Genomic DNA of each clone was submitted to Microbial Genome Sequencing Center (MiGS) for WGS using the Illumina NextSeq 2000 platform.

### Genome Assembly, Gene Prediction, and Annotation.

Reads were trimmed using Trimmomatic v0.36 (77) and assembled de novo with SPAdes version 3.11.1 (78). Coding DNA sequences (CDS) from the assembled contigs were predicted using Prodigal version 2.6.3 (79). Predicted proteins were annotated against the nonredundant protein sequences from the NCBI database using DIAMOND (80).

### Recovery and Phylogeny of *V. cholerae* Genomes.

A total of 1,804 *V. cholerae* genomes were downloaded from RefSeq (accessed April 2024) and subjected to a dereplication step with dRep (81) at 99% nucleotide identity. Single nucleotide polymorphisms among *V. cholerae* dereplicated genomes and the PCG reference genome *V. cholerae* C6706 (GCF_015482825.1) were calculated with PhaME (82). Then, a maximum likelihood phylogenetic tree of SNPs was constructed using iqtree v 1.6.12 (83). To group the dereplicated genomes, the SNP alignment was converted into a similarity matrix using the package bio3d (84) in R and represented in a principal component analysis (PCA) using the FactoMineR package (85).

### Construction of Mutant Strains.

*V. cholerae* constructs were all generated in the El Tor strain C6706 background (*SI Appendix*, Table S4) following previously published methods (19). Isogenic *tcpF* mutants described in Fig. 5*C* were also constructed in the background of *V. cholerae* O395 and *V. cholerae* 2010EL-1786. Briefly, the environmental alleles of interest were synthesized commercially (Gene Universal and Twist Biosciences). The synthesized products were cloned into the *V. cholerae* suicide vector pKAS154, electroporated into *Escherichia coli* S17λ-*pir*. Following sequence verification, allelic exchange was carried out in *V. cholerae* C6706 using the appropriate antibiotic selection, as described (86). Fluorescent tagging of the strains was performed using the mini-Tn7 system that integrates at the *att* site between the *glmS* (VC_0487) and VC_0488 genes in *V. cholerae* as previously (87).

### Infant Mouse Colonization Assays.

All experiments involving animals were reviewed and approved by the Institutional Animal Care and Use Committee of the University of Central Florida (IPROTO202300049). Competition assays were performed in 3- to- 5-d-old infant mice as previously described (19, 88, 89). The test (*lacZ*^+^) and control (*lacZ*^-^) strains were mixed 1:1 and inoculated orogastrically. The relative colonization efficiencies are presented as the competitive index (CI), which is the ratio of the intestinal *lacZ*^+^ CFU (blue colonies) to *lacZ*^-^ CFU (white colonies) normalized to the input CFU ratio and in vitro competition CFU ratio.

### Colonization of Environmental Hosts.

Bacterial inocula for infections of the environmental hosts were prepared essentially as described for the mice colonization assays. a) *Crustaceans*. Eggs of *A. salina* were hatched in artificial sea water supplemented with 2% sodium chloride (ASW). Infections were performed in 200 µL ASW containing 20-30 individual crustaceans and the 10^7^ CFU/mL of bacteria. After incubation samples were washed twice in ASW and either homogenized for determining CIs or used directly for microscopy. b) *Cyanobacteria*. *M. aeruginosa* (UTEX LB 2385) were incubated in flasks containing BG-11 media (Gibco) under a 12-h light–dark cycle with aeration for 14 d. *M. aeruginosa* cells were concentrated and resuspended in BG-11 and cocultured with 10^7^ CFU/mL *V. cholerae* cells. Samples were harvested and plated for CFU count and CI determination. c) *Mollusks*. Mucus-containing gills of the Eastern oyster *C. virginica* were harvested, homogenized, and autoclaved (73). M9 minimal medium was supplemented with 0.1% sterile gill homogenate and inoculated with 10^7^ CFU/mL of bacterial inoculum. Samples were incubated and plated for CFU count and CI determination.

## Supplementary Material

Appendix 01 (PDF)

## Data Availability

The genomes have been deposited under BioProject PRJNA1035537 (40).
