# Supplementary material for: Allelic variations and gene cluster modularity act as nonlinear bottlenecks for cholera emergence
Source: Proc Natl Acad Sci U S A. 2025 May 28;122(22):e2417915122. doi: 10.1073/pnas.2417915122 (PMC12146696; doi:10.1073/pnas.2417915122)
Supplement: Supplementary file 1 — Appendix 01 (PDF) [file pnas.2417915122.sapp.pdf]

SUPPORTING INFORMATION APPENDIX

Allelic variations and gene cluster modularity act as non-linear bottlenecks for cholera emergence

Mario López-Pérez[a,b#], Deepak Balasubramanian[a,c#], Alicia Campos-Lopez[a], Cole Crist[a], Trudy-Ann Grant[a], Jose M. Haro-Moreno[a,b], Asier Zaragoza-Solas[a,b] and Salvador Almagro-Moreno[a,c*]

[a]Burnett School of Biomedical Sciences, College of Medicine, University of Central Florida, Orlando, Florida, USA; [b]Microbial Genomics and Evolution Group, División de Microbiología, Universidad Miguel Hernández, Alicante, Spain. [c]Department of Host-Microbe Interactions, St. Jude Children's Research Hospital, Memphis, Tennessee, USA.

# Contributed equally

*Address correspondence to: Salvador Almagro-Moreno, samoreno@stjude.org

Running title: Evolutionary bottlenecks of pathogen emergence

RESULTS AND DISCUSSION

Isolation of environmental *Vibrio cholerae* from the Indian River Lagoon. *V. cholerae* is a natural inhabitant of aquatic ecosystems where it can be found as free-living or attached to abiotic or biotic surfaces (1, 2). We recently established two sampling sites along the eastern coast of Florida where we isolated pathogenic strains of *Vibrio vulnificus*(3). We sampled those two sites, Feller's House (FH) and Shepard Park (SP), to investigate the potential presence of *V. cholerae* strains (**Fig. S1A**). Using the isolation, enrichment and selection methods described below, we identified a total of 178 potential *V. cholerae* isolates from the different fractions at both locations during our sampling events (42 FH and 136 SP). To confirm those isolates, we performed a PCR screening for the species-specific gene *ompW* (4). Only 65 isolates were confirmed as *bona fide V. cholerae*, all of them isolated from SP (**Fig. S1A**). *V. cholerae* isolates from SP were collected from all the fractions that we examined (sediment, cyanobacteria, particle-associated and water) except for oysters. Subsequently, we performed PCR-based fingerprinting of the strains to reduce redundancies and minimize clonality. We used a combination of PCR-based fingerprinting analyses, VCR-PCR and ERIC-PCR (5). Based on fingerprinting patterns and cluster analysis performed using BIONUMERICS v8.0 we generated a dendrogram to group these observed patterns. Using this resulting dataset, we selected 35 isolates with unique fingerprinting patterns for whole genome sequencing (**Fig. S1B and Table S1**).

Hierarchical classification and clustering of the *V. cholerae* species. *V. cholerae* is a natural inhabitant of aquatic ecosystems where it can be found as free-living or attached to abiotic or biotic surfaces (1, 2). We isolated and sequenced the genomes of 35 *V. cholerae* strains from a large estuary in eastern Florida from a diverse set of fractions: 6 from sediment, 10 from cyanobacteria, 9 from the free-living water fraction and 10 from the particle-associated one (**Supporting text, Table S1**). We examined the relationship of these strains with *V. cholerae* PCG by analyzing the average nucleotide

identity (ANI) in a pairwise comparison against *V. cholerae* C6706, a reference O1 strain from the El Tor biotype (**Fig. S1C**) (6). The minimum values obtained were ca. 97.5, always within the established cut-off for strains of the same species. However, up to nine environmental strains showed a greater divergence with the PCG (ANI >98.2%) than with the rest of the strains (**Fig. S1C**). Interestingly, phylogenomic analysis based on whole-genome single nucleotide polymorphism (SNP) show that the majority of the IRL strains form two major clusters, one of them including the toxigenic strain of *V. cholerae* (**Fig. S1D**). This evolutionary relationship shares similarity with that of *V. vulnificus*, in which the pathogenic strains capable of causing septicemia in humans belong to one clearly defined cluster (7). This prompted us to further characterize the *V. cholerae* species to investigate whether the evolution of the toxigenic strains follow a similar cluster-based pattern, as this would shed important light on the emergence and evolution of the pathogen.

First, we collected the *V. cholerae* genomes available in public databases (based on NCBI RefSeq classification accessed in April 2024; see Materials and Methods) together with our environmental isolates. Naturally, there is a significant bias in the databases towards *V. cholerae* O1 and O139 strains, which are mostly clonal. Therefore, in order to obtain an unbiased phylogeny, we dereplicated all these genomes to an identity of ANI >99%. From a total of 1,840 initial genomes, we obtained 378 non-clonal reference *V. cholerae* clades (**Table S2**). To maximize the consistency of our analyses, we built both a SNP-based phylogeny and genome clustering using ANI to determine the groups that constitute the *V. cholerae* species. The application of a divergence threshold of less than 97% in ANI results in a single cluster indicating that the boundary of intra-population sequence diversity in *V. cholerae* species is 97%. Next, we used a total of 486,015 SNPs from a core genome size of 2.34 Mb to build the species phylogeny using non-clonal reference clades and IRL strains (**Fig. 1A**). The detailed phylogenetic tree and metadata of the final genome dataset are shown in Figure S2 and Table S2. Subsequently, we clustered the genomes within the phylogenetic tree according to a

similarity matrix using the SNP alignment. Unsupervised hierarchical clustering split the data into eleven well-defined group (**Fig. 1B** and **Table S2**). All IRL isolates are grouped together with previously isolated strains except for strains IRLE0050 and IRLE0074 which formed a separate group (G8) (**Table S2**). Group 3 (G3) is the largest of the groups containing 142 non-clonal clades (*ca*. 38% of the total) including PCG (**Fig. 1A**). We did not find a pattern that associate the phylogeny of the IRL isolates and their fraction of isolation. The same analytical approach used to identify groups within the entire species was applied to G3. This new clustering reveals nine subgroups within G3. In one of those subclades, what we have term the Pandemic Cholera Lineage (PCL), the PCG branches together with 33 other strains, including six IRL strains from this study (**Fig. 1C**). This provides us with a novel hierarchical approach based on three levels to decipher the evolutionary history of toxigenic strains of *V. cholerae* (from highest to lowest): a) the G3 within all *V. cholerae* strains, b) the PCL within G3 non-clonal clades and c) the clonal *V. cholerae* toxigenic strains within the PCL lineage.

Restricted distribution of O1 lipopolysaccharide cluster in environmental *V. cholerae* strains. The LPS plays numerous roles in the pathogenesis of toxigenic *V. cholerae* (e.g., endotoxic activities, immunological responses, etc. (8–10)) and is critical to determine the antigenic properties and classification of the bacterium (10). Specifically, given that only O1 strains, together with O139, can cause pandemic cholera, we examined its LPS cluster to determine its distribution, evolutionary history, and allelic diversity within the *V. cholerae* species. The O1 gene cluster is 28 Kb long and encompasses approximately 25 ORFs with potential or demonstrated function (some are truncated preventing an exact number). The *rfa* gene cluster (LPS core synthesis) is encoded at the 5' end of the island (**Fig. 2A** blue arrows) and is widely distributed across all clades, with the notable exception of RfaF (ADP-heptose--LPS heptosyltransferase 2, VC_0235) and RfaL (O-antigen ligase, VC_0237) present in ca. 35 non-clonal clades. Nonetheless, the genes in the *rfa* cluster exhibit the highest divergence within the LPS

cluster (median identity 86-88%) and accumulate numerous synonymous polymorphisms (**Fig. 2A**). This indicates that, like in *V. vulnificus*, the *rfa* cluster appears to be a preferred site to act as hot spots for the recombination of this island due to the conservation and synteny of these genes (7). The distribution of the central region of the LPS cluster is much more restricted and is only present in 16 *V. cholerae* non-clonal clades from three clusters (G1, G3 and G4), however, those that encode it share 100% identity with the region of PCG. This region contains the *rfb* cluster which is associated with antigen-O synthesis(11) (**Fig. 2A,** green arrows). Interestingly, only 2 out the 16 clades that encode this region belong to the PCL, indicating that there is no direct correlation between the phylogeny and its presence in PCG (**Table S2**). The PCG encodes two transposases at the end of the *rfb* cluster, which likely increases the variability within the island (**Fig. 2A**). These IS elements are widely distributed among strains of *V. cholerae*, which provides insights into how small groups of genes can be introduced into the island. This is one of the distinguishing characteristics of the PCG strains (12, 13). For instance, strain N16961 encodes two additional transposases in the same region, which are inserted into the *rfb*T gene (**Fig. S3A**). This results in a truncated, non-functional version of the gene. C6706 also has a truncated version of the same gene. However, this is due to a mutation that reduces the size of the gene from 861 bp (like the VC22 version) to 504 bp (**Fig. S3A**). *rfbT* encodes a methyltransferase that is responsible for seroconversion between *V. cholerae* O1 Ogawa and Inaba serotypes, suggesting a major role for these IS sequences in the emergence of PCG (12–14). Furthermore, several strains from G4 encode the same version of the hypervariable region as PCG (identity 99%) but the *rfa* cluster is different (**Fig. S3A**). The first three genes of the island encode different alleles of *rfaF*, *rfaG* and *rfaL*, and the identity of the remaining three is 95% (**Fig. S3A**). The *rfa* cluster pattern of these strains is similar to that of strain A325 (G4) (**Table S2**). In addition, in this strain, the two transposases that truncate the *rfb*T gene in the PCG are inverted maintaining an intact version of the gene (**Fig. 2C**). Overall, we found a remarkable degree of conservation of the LPS cluster, which is present in

almost sixteen clades encoding a PCG-like version. This is particularly striking as LPS hypervariability is a universal bacterial characteristic, suggesting some strong evolutionary pressure maintaining this version of the island widespread in the population. It appears that the island is a) exchanged by homologous recombination leading to the whole gene cluster being conserved and b) the presence of IS elements increases the variability by truncating genes such as *rfbT*.

Virulence-associated mobile elements exhibit irregular distribution and modularity in *V. cholerae* species. There are several horizontally acquired MGEs that are directly associated with the emergence of toxigenic clones of *V. cholerae*. After we established the phylogeny and clustering of the *V. cholerae* species, we investigated the distribution, evolutionary dynamics, and allelic diversity of these elements within the population.

1. CTXΦ phage. The genes for cholera toxin (CT) are encoded within the CTXΦ phage(15). The production of CT in the intestine is responsible for the severity of the profuse diarrhea associated with cholera. CT leads to the secretion of electrolytes and nutrients such as fatty acids into the lumen of the small intestine (16–18). Due to the lysogenic nature of CTXΦ, the insertion and deletion dynamics of this phage enables recombination of genes, which leads to diversity within the toxigenic strains ultimately serving as an opportunity to increase their pathogenic potential (15, 19, 20). Inquiry into the distribution and composition of this element within *V. cholerae* shows that there is no environmental strain that recapitulates all the phage genes of PCG. At most, six non-clonal clades and three clusters encode 10 of the 14 genes that make up the CTXΦ phage of *V. cholerae* C6706: three from G3 (represented by strains AAS91, PivertUAT4Aug and V060002) and two from G4 (3541-04 and 3528-08) and one from G1 (458_01) (**Fig. 2B** and **Table S3**). Four of these strains encode the two subunits of the cholera toxin (CtxAB). However, genes coding for the duplicated transcriptional regulator RstR (VC_1455 and VC_1464) are only found in one strain: AAS91 (**Fig. 2B**,

brown shade). This strain belongs to the PCL and encodes the complete version of CTXΦ except for VC_1460 that encodes a minor coat protein pIII related to host interaction as part of the infection process (**Fig. S3B**) (21). Instead, a transposase has been inserted in this locus in strain AAS91, followed by a APH(3')-II family aminoglycoside O-phosphotransferase (**Fig. S3B**). In addition, AAS91 has a third copy of the triplet (RstA-RstB-RstR) instead of the two copies present in PCG strains (**Fig. S3B**). The second most complete version of CTXΦ in *V. cholerae* is found in strains V060002 from PCL and 3528-08 (G4). Both strains have lost the gene coding for RstC and encode a different allele of the transcriptional regulator RstR (**Fig. S3B**).

Genomic comparison of *V. cholerae* strains reveal that the phage variation dynamics are of the "additive" kind, where small gene cassettes are integrated into the 3' part of the gene coding for RtxA (22). All the CTXΦ variants have conserved intergenic regions that separate and mark the insertion of these gene cassettes (*att* sites, marked as black arrows). These types of insertions have been reported previously in *V. cholerae* O139 (23). Interestingly, some strains such as 3528-08 (G4) can have up to five insertions (**Fig. S3C**). The first two of the five cassettes are identical, and each cassette has a copy of the two subunits of CT. The other three have lost the toxin genes and those coding for RstB, RstR and RstA, and acquired a replication endonuclease (**Fig. S3C**). The presence/absence of these cassettes is not correlated with the phylogeny and, when present, they have high identity indicating a fast turnover of these elements (**Fig. S3B**). To date, the potential complex role of the accumulation and exchange of these cassettes in the evolution of toxigenic strains remains to be fully elucidated. Furthermore, these cassettes can be integrated into other genome locations, as in the case of the strain PivertUAT4Aug (G3) (**Fig. S3B**). In addition, using the sequence of *att* sites as reference we also found a collection of cassettes in the same order (including the one containing the two subunits of CT) in the close relative species *Vibrio mimicus*, suggesting another potential donor of CTXΦ to non-toxigenic *V. cholerae* (**Fig S3D**).

2. Vibrio Pathogenicity Island-1 (VPI-1). The toxin coregulated pilus (TCP), an essential colonization factor, is encoded within VPI-1 (24, 25). TCP mediates microcolony formation, which is crucial for intestinal colonization, and acts as the receptor of CTXΦ (26). VPI-1 also encodes two critical virulence regulators: ToxT and TcpP (25, 26). VPI-1 can be transferred, via generalized transduction, between strains of *V. cholerae* and can also form circular intermediates (27, 28). The variable region of the island is located between the CDS VC_0821 (hypothetical protein) and VC_0846 (integrase) (**Fig. 2C**). The most conserved version of this region can be found in eleven clades, five of them within G3 (2 from PCL), five from G4 and one from the G7 (**Fig. 2C** and **Fig. S3E**), including strain IRLE0081 isolated in this study. The average identity of the proteins was highly conserved compared to PCG (*ca*. >97%), however, we found some notable exceptions. First, among the proteins that are part of the TCP operon, we found high divergence rates in the master regulator ToxT (VC_0838) and the toxin-coregulated pilus major pilin, TcpA (VC_0828) with a median protein identity *ca*. 81% (**Fig. 2C**). In addition, we found that the PCG allele of the colonization factor TcpF (VC_0837) was only present in three strains (one PCL and two G3). In the rest, despite the conservation of synteny and similarity of the whole TCP operon, the identity of this protein was less than 40%, encoding a different allele of this poorly understood secreted protein (29–31) (**Fig. S3E**). Finally, our genomic comparisons reveal that the gene coding for ToxR-activated gene A, TagA (VC_0820) was absent from the G4 genomes, while being retained in three of the five G3 genomes (**Fig. S3E**). Overall, despite widespread conservation in the synteny and size of the clusters of genes that comprise VPI-1 in the *V. cholerae* species, the existence of unique major allelic variations in a limited number of genes might act as a bottleneck in the emergence of PCG.

3. Vibrio Pathogenicity Island-2 (VPI-2). VPI-2 is a large PAI that encodes the genes for sialic acid utilization (32). The capacity to utilize sialic acid as a carbon and

energy source confers *V. cholerae* a competitive advantage in the mucus-rich environment of the gut, where sialic acid availability is extensive (33). Sialic acid catabolism also mediates a chemotactic response towards mucin and several environmental reservoirs of *V. cholerae* (34). VPI-2 is excisable and form a circular intermediate (28, 35). Interestingly, during the excision event, there is crosstalk between VPI-2 and VPI-1 (36). Unlike other MGEs, the differential abundance of the gene clusters within VPI-2 suggests that the island is made up of different modules. Also, all but one of the IS elements of the island are widespread among several clades in diverse locations, which favours the transfer of the different modules. The canonical VPI-2 from O1 strains encodes three major modules, from 5' to 3': a) a restriction modification system (RM), b) the Nan-Nag cluster, and c) a Mu-phage like region (**Fig. 2D**). At the 3' end of VPI-2 there is also a small group of genes encoding hypothetical proteins. The Nan-Nag cluster is present in 115 non-clonal clades, whereas the distribution of the RM system is more limited, being encoded by only five clades (**Fig. 2D** and **Table S2**). The Mu-phage like module is present in twelve clades, however, there is no relationship between specific modules and species phylogeny. Like VPI-1, no clade other than PCG encode a PCG-like version of VPI-2. Furthermore, strains that contain the three individual modules sometimes encode them in different locations within the genome. For instance, strain PivertUAT4Aug (G3), the only one with the three major modules, encode the first two modules (Nan-Nag and RM) within a version of VPI-2, whereas the Mu-phage region is encoded in a different location of the genome (**Fig. S3F**). In the Mu-phage like module VC_1788 and VC_1791 comprise two parts of a gene coding for the tape measure protein that have been truncated by the insertion of two transposases in the PCG, annotated as VC_1789 and VC_1790 (**Fig. 2D**). Although the synteny in the modules was conserved, and with high similarity (**Fig. S3F**), we found a high divergence in the VC_1768 gene coding for a restriction endonuclease S subunit (HsdS) between PivertUAT4Aug and the PCG-like version of VPI-2 with a protein identity of 64% (**Fig. S4D**). Another instance of this modular distribution is exemplified by strain 17-VB00206

(G1), which possesses the first two modules but not the Mu-phage. Furthermore, the strain has lost the gene encoding the HsdS proteins from the RM cluster involved in DNA sequence recognition (**Fig. S3F**). Strain 3541-04 (G4) encodes a different cluster of genes at the insertion site of the Nan-Nag cluster and RM system, with the Nan-Nag cluster being inserted in another chromosomal region (**Fig. S3F**). Overall, VPI-2 in PCG must be understood based on its unique modular arrangement not by the individual units that constitute it and the possibility of allelic variability in *hsdS* playing a unique role in the pandemic group. Its four modules are associated with IS elements that are widely distributed among *V. cholerae* environmental strains and scattered throughout the genome. This leads to the convergence of all modules in the same genome being extremely low which contributes to the rarity of PCG.

4. Vibrio Seventh Pandemic Island I (VSP-I). The role of VSP-I in *V. cholerae* pathogenesis is not as clearly defined as other MGEs. VSP-I has been suggested to play an environmental role related to chemotaxis (37) and encodes a regulator involved in intestinal colonization (VC_0177) (38). We found homologs of the eleven genes that comprise this island in eleven *V. cholerae* clades (**Fig. 2E** and **Table S2**). Although three of them are found in strains isolated from this study, IRLE0062, IRLE0049, and IRLE0077, that belonged to the same clade (G9). The greatest sequence divergence corresponds to its integrase (VC_0183) and an XRE family transcriptional regulator (VC_0176) (**Fig. S4E**) encoded within the island (**Fig. 2E**). Interestingly, only two strains encode a PCG-like VSP-I, V060002 (G3), and VcCHNf4 (G6). Surprisingly, V060002 encodes two identical copies of VSP-I in close proximity to each other with the LPS cluster located between them (**Fig. S3G**). The landscape of evolutionary possibilities such as modularity is much more limited for VSP-I than for the other MGEs due to its small size. It is also the rarest of all the islands with one transcriptional regulator, VC_0176, exhibiting high allelic diversity and very limited distribution. The rarity of the

island and diversity of the regulator suggests that this is a critical bottleneck in the emergence of the Seventh Pandemic El Tor strains.

5. *Vibrio Seventh Pandemic Island II (VSP-II).* The presence of VSP-II in clinical and environmental strains might be associated with environmental survival and fitness of the bacterium (39–41). A group of *V. cholerae* strains that caused an outbreak in Florida associated with oyster consumption encoded a novel bacteriocin and a pyocin within the VSP-II element, proteins typically associated with bacterial competition(41). Our analyses indicate that, similar to VPI-2, VSP-II is highly modular with several clusters of genes that have a tRNA-Met insertion point. The direct repetition of the end of the tRNA (highlighted as purple triangles) marks the insertion points of each module, which in most cases match the presence of IS elements (**Fig. 2F and S3H**). Overall, this island serves as another example of an "additive" island with three small modules. Module 1 comprises genes VC_0490 to VC_0498, five of which are of viral origin but lack essential proteins for packaging, indicating that it is a defective prophage. Module 2 encompasses VC_0502 to VC_0510, however, the lack of possible annotation of its genes precludes from assigning a specific function to this module. Nonetheless, VC_0502 and VC_0503 from this module are only present in strains from G3 (**Fig. 2F** and **Table S2**). While islands are mostly conserved between *V. cholerae* PCG strains, Module 3 of VSP-II exhibits a distinct sequence. The Peruvian strain has replaced the five genes (VC_0511 to VC_0515) with a completely different cluster of four genes(42). The C6706 variant was only located in two strains from G1 (458_01 and R18275) (**Table S2**). The VSP-II variant of strain C6706 is illustrated in Figure 2F whereas the one encoded by other non-Peruvian PCG strains is depicted in Figure S3H. Leaving aside Module 3, the closest versions are encoded by strains N2723, MZO-3, and V060002 belonging to G3, which are the only ones that possess a similar PCG-like modularity and synteny of VSP-II (**Fig. S3H**). N2723 has lost genes coding for two hypothetical proteins (VC_0496 and VC_0509) and a transcriptional regulator (VC_0497) and VSP-II from strain MZO-3 lost

a transposase (VC_0501) in Module 1 and gained another in Module 3 (**Fig. S3H**). In the rest of *V. cholerae* strains, VSP-II only maintains some of these modules or clusters of genes, with the addition of other types of clusters being common. Finally, our analyses did not reveal any gene that exhibits high rates of divergence within the island, suggesting allelic diversity might not act as a major constraint in this element.

Pangenome dynamics of *V. cholerae* and evolutionary trajectories of PCL. Our results indicate that modular acquisition of MGEs and allelic variations within them are critical processes for PCG emergence. To uncover other potential genes that might play a role in this phenomenon we implemented a pangenomic approach. First, we investigated the unique genes encoded by PCG and those that the group inherited from its closer ancestors. We considered the three major hierarchical levels in which the toxigenic strains of *V. cholerae* are included: group (G3), pandemic cholera lineage (PCL), and the pandemic cholera group (PCG) (**Fig. 1A**). We analyzed the sequences defining each of these groups at the pangenome scale, using the remaining set of clades as outgroups to remove the background of other environmental *V. cholerae* sequences. The Venn diagram in **Figure S7A** shows the total unique and shared gene family clusters among the pangenomes of the different groups analyzed. The total number of shared clusters of the *V. cholerae* species is 3,222 (core genome). The PCG encodes 109 unique gene families, 28% of which (31/109) are located in the integron of chromosome II. Among the remaining specific clusters that are not encoded within the integron, some of them are localized in MGEs (**Table S3**). From the three major hierarchical levels, PCG inherited 17 genes from the PCL, of which seven are also concentrated in the integron and two are present in PAIs: the transcriptional repressor RstR of CTXΦ (VC_1464) and the transposase OrfAB, subunit Afrom VPI-2 (VC_1790) (**Table S3**). Finally, PCG inherited 58 unique gene families from G3 (**Fig. S7A**). Among these 58 genes, 11 genes are concentrated in the integron (**Table S3**).

Next, in order to understand the evolution of the PCG from the root of the PCL, we analyzed the acquisition history of the MGEs and their modules throughout the lineage (**Fig. S7B**). Our analysis indicates that only the PCG representative (V060002) has the same MGE combination as C6706, with minor exceptions such as the lack of three genes in CTX. The closest PCG relative (AAS91) is the only one that maintains LPS, CTX and VPI-1 (**Fig. S7B**). In the remainder of the PCL representatives, only module 2 of VPI-2, the Nan-Nag cluster, was located in one-third of the strains (**Fig. S7B**). Our results demonstrate that the acquisition of mobile elements in the *V. cholerae* species leading to the emergence of toxigenic clones has been a mosaic-like process rather than a progressive evolutionary one.

MATERIALS AND METHODS

Sampling sites. We collected samples at two environmentally distinctive locations along the IRL (Eastern Florida, USA) in three sampling events. For each sampling campaign, we obtained samples from several fractions: three associated with the water bodies (water filtered through 20µm, 5µm, and 0.22µm) and one from the sediment. One of the sampling locations, Fellers House Field Station (N28˚54'25.315"; W80˚49'15.017"), is located within the federally protected Canaveral National Seashore. The second sampling site, Shepard Park, is located in the Port St. Lucie area (N27˚11'48.864"; W80˚15'33.172") that due to urbanization and agricultural expansion, experiences nutrient over-enrichment leading to excessive macroalgal bloom (**Fig. S1A**) (3).

Isolation of *V. cholerae.* a). _Water._ Isolation of *V. cholerae* from water samples collected at the sampling locations was performed using a modified protocol from Huq *et al* (43). Briefly, water samples (~3L) were collected in triplicate from each site and stored cold until arrival at the laboratory. To investigate the prevalence of *V. cholerae* in various fractions of the aquatic column and to separate particle-associated and free-living bacteria, 500 mL of collected water was filtered successively through 20µm, 5µm and 0.2µm filters (Sterlitech) using a vacuum filtration system. The respective membrane filters harboring potential *vibrio* spp. were then suspended in 25ml phosphate buffered saline (PBS) and vortexed vigorously to release the contents. Each resuspended sample was then inoculated (1:10 v/v) in alkaline peptone water (APW) and incubated at 37°C for 12-14 hrs. This was repeated for each replicate. Enriched cultures were subjected to growth on a series of selective agar specific for the isolation of *V. cholerae.* First, enriched cultures were serially diluted 10-fold and plated on CHROMagar Vibrio (CaV; CHROMagar), a chromogenic media on which *V. cholerae* forms turquoise blue colonies. Subsequently, turquoise blue colonies from CaV were transferred onto Thiosulfate Citrate Bile Salts Sucrose (TCBS; Sigma) agar plates. Colonies that appeared turquoise blue on CaV followed by yellow on TCBS were preliminarily identified to be *V. cholerae*.

b) _Sediment_. Isolation of *V. cholerae* was done using established methods (3). Briefly, sediment samples were collected in triplicates from each sampling site using universal corer. 25 g of the sediment samples from each replicate was weighed and suspended in 25 ml of PBS (Gibco). Samples were homogenized, enriched, and studied for the presence of *V. cholerae* as described above. **c)** _Cyanobacteria_. Cyanobacteria floccules were pelleted, supernatant removed and fresh APW added to homogenize the sample. Samples were enriched and studied for the presence of *V. cholerae* as described above.

Strains and growth conditions. *V. cholerae* strains were routinely grown on Luria Bertani (LB) agar at 37 ºC for approximately 16hrs, unless otherwise stated. For routine liquid cultures, isolated colonies selected from agar plates were grown aerobically at 37ºC in LB broth for ~16hrs. Tryptone broth (tryptone 10 g/L, NaCl 5 g/L), artificial sea water and M9 minimal media (Fisher) supplemented with 0.1% glycerol were used for biofilm formation, studies using *Artemia* and oyster gill homogenates, respectively. Growth media was supplemented with the following antibiotics and reagents, as needed: polymyxin B 50 U/mL, kanamycin 45 µg/mL, streptomycin 1000 µg/mL, gentamycin 15 µg/mL, X-gal 40 µg/mL.

Clonal analysis of *V. cholerae* isolates. To confirm isolates of *V. cholerae,* presence of the species-specific gene *ompW* was determined by PCR(4). Genomic DNA from the isolates was extracted using the Gentra Puregene Yeast/Bact Kit (Qiagen) and used as template for PCRs. To identify the clonal populations of *V.* cholerae in the confirmed dataset, DNA fingerprinting patterns were examined using a combination of *Vibrio cholerae* Repeats-PCR (VCR-PCR) and Enterobacterial Repetitive Intergenic Consensus Sequence (ERIC)-PCR as described by Teh *et al* (5). Genomic DNA was used to perform VCR- and ERIC-PCR and resulting PCR products were electrophoresed and imaged using UVP ChemStudio (AnalytikJena) to observe fingerprinting patterns. Cluster analysis of DNA patterns was performed using BioNumerics v8.0 (Applied Maths,

Inc.) and dendrogram generated based on the Unweighted Pair Group Method with the Arithmetic Mean (UPGMA) method (5, 44, 45) (**Fig. S1B).** Based on resulting DNA fingerprinting patterns, a total of 35 independent clones were identified and selected for whole genome sequencing (WGS) (**Table S1**). Genomic DNA of each clone was submitted to Microbial Genome Sequencing Center (MiGS) for WGS using the Illumina NextSeq 2000 platform.

Genome assembly, gene prediction, and annotation. Reads were trimmed using Trimmomatic v0.36 (46) and assembled de novo with SPAdes version 3.11.1 (47). Coding DNA sequences (CDS) from the assembled contigs were predicted using Prodigal version 2.6.3 using *-a output.proteins -d output.genes -c -p meta* parameters (48). Then, tRNAs were obtained using tRNAscan-SE version 1.4 (49) together with ssu-align version 0.1.1, and rRNA genes using meta-rna (50). Predicted proteins were compared against the National Center for Biotechnology Information nonredundant database (NCBI nr) using DIAMOND (51). In addition, for functional annotation we used HMMscan version 3.1b2(52) for the comparison against COG v2003 (update 2014) (53) and TIGFRAM v15.0 (September 2014) (54) databases.

Recovery of *V. cholerae* genomes. A total of 1,804 *V. cholerae* genomes were downloaded from RefSeq (accessed April 2024) and subjected to a dereplication step with dRep (55) at 99 % nucleotide identity. Briefly, we force dRep to sort and select as seeds for the clustering genomes with better score. For instance, chromosomes in a single contig, followed by higher N50 and L50 values, and lastly by the completeness and contamination values calculated with checkM (56). This dereplication step resulted in 378 dereplicated reference *V. cholerae* genomes.

Phylogeny of *V. cholerae* dereplicated genomes. Single nucleotide polymorphisms (SNPs) among *V. cholerae* dereplicated genomes and the PCG reference genome *V.*

cholerae O1 El Tor strain C6706 (RefSeq accession number GCF_015482825.1) were calculated with PhaME (57) with default options. Then, a maximum likelihood phylogenetic tree of SNPs was constructed using iqtree v 1.6.12 (58) using the ultrafast bootstrap approach (5000 replicates) and the best fitted model GTR+F+R4. To group the dereplicated genomes, the SNP alignment was converted into a similarity matrix using the package bio3d (59) in R and represented in a principal component analysis (PCA) using the FactoMineR package (60). Groups were identified using the K-means approach, which fixes the number of groups (k), according to the user criterium. We followed an unsupervised approach on which we iterate from k=1 until k=100 and selecting the best k number (k=15) considering that the total within-cluster sum of squares between the data points and their corresponding centroids (Euclidean distance) decreased the lowest after this cluster and reached a minimum.

Abundance and distribution of pathogenicity-related islands. To determine virulence-associated mobile genetic elements and LPS cluster distribution and degree of prevalence of proteins within the studied *V. cholerae* genomes, we extracted these genomic regions from PCG reference genome (*V. cholerae* El Tor strain C6706) based on the manual annotation found in the NCBI Genbank. Then, proteins were extracted and used as a reference in a BLASTP search against the proteins from the rest of the *V. cholerae* species genomes (61, 62). We kept all proteins that matched against the pathogenic protein database with at least 70% identity and the alignment covered at least 70% of both proteins.

Construction of mutant strains. *V. cholerae* constructs were all generated in the El Tor strain C6706 background following previously published methods (63). Isogenic *tcpF* mutants described in Figure 5C were also constructed in the background of *V. cholerae* O395 and *V. cholerae* 2010EL-1786. Briefly, the environmental alleles of interest were synthesized commercially (Gene Universal and Twist Biosciences) including 500 bp

flanking regions from *V. cholerae* C6706 to facilitate recombination. The synthesized products were digested and ligated into the *V. cholerae* suicide vector pKAS154, electroporated into *Escherichia coli* S17λ-*pir* and selected on kanamycin plates. Following sequence verification, allelic exchange was carried out in *V. cholerae* C6706 using the appropriate antibiotic selection, as described (64). Positive clones were first screened by colony PCR using flanking primers and confirmed by sequencing the exchange locus from purified genomic DNA. Fluorescent tagging of the strains was performed using the mini-Tn7 system that integrates at the *att* site between the *glmS* (*VC0487*) and *VC0488* genes in *V. cholerae* (65). Briefly, triparental mating was performed using LB-grown overnight cultures of the donor *E. coli* S17λ-*pir* cells harboring pUC18T-mini-Tn7T-GFP or RFP expressed from a strong constitutive promoter, with the recipient *V. cholerae* cells and helper *E. coli* encoding the transposase gene on a suicide vector. The mating mixture were selected twice on LB-Pb-Gm plates and followed by two rounds of selection on LB-Sm plates, and subsequently confirmed by sequencing the Tn7 insertion site and fluorescence microscopy.

Infant mouse colonization assays. All experiments involving animals were reviewed and approved by the Institutional Animal Care and Use Committee of the University of Central Florida (IPROTO202300049). Intestinal colonization competition assays in three-to-five-day-old infant mice were performed with LB-grown *lacZ*$^+$ cells of the WT and allelic exchange mutants competed against an isogenic WT strain harboring a *lacZ* deletion, essentially as described (63, 66, 67). The colonization efficiencies of the different strains in the small intestine harvested post infection are represented as competitive indices. Briefly, strains were inoculated from fresh LB plates into LB-Sm broth, incubated for 12 hours at 37 ˚C with aeration, and diluted 1:1000 fold in 1x PBS (pH 7.4, Gibco). The test (*lacZ*$^+$) and control (*lacZ*$^-$) strains were mixed 1:1 to obtain ~10^6 CFU/mL. To monitor successful inoculation, Evan's blue dye (0.01% final, Sigma) was added to the cell mixture. Fifty microliters of the bacterial mixture, corresponding to ~10^5 CFUs, were then

used to intragastrically inoculate 3-5-day-old infant mice and maintained at 30 ˚C post-infection. The exact input CFU numbers were determined by plating dilutions of the inoculum on LB-Sm-X-Gal plates. Approximately 22 hours post infection, the mice were euthanized, their small intestines harvested and transferred into 4 mL LB-10% glycerol stored on ice. The samples were then processed for CFU determination by homogenizing the tissues, serially diluting in 1x PBS (pH 7.4) and plating appropriate dilutions on LB-Sm-X-Gal plates. *In vitro* competition experiments were performed in parallel by inoculating 50 µL of the input CFU in LB-Sm broth and incubating for 22 hours at 37 ˚C with aeration and enumerating CFUs as above. Results are presented as the competitive index (CI), which is the ratio of the intestinal *lacZ*$^{+}$ CFU (blue colonies) to *lacZ*$^{-}$ CFU (white colonies) normalized to the input CFU ratio and *in vitro* competition CFU ratio.

Colonization of environmental hosts. Bacterial inocula for infections of the environmental hosts were prepared essentially as described for the mice colonization assays but used at different dilutions and without the Evan's blue dye. Coinfections were performed with GFP-tagged *lacZ*-positive test strains competing against RFP-tagged wild type strain harboring a *lacZ* deletion. **a)** _Crustaceans_. We modified previously established protocols as follows (68), eggs of *A. salina* were hatched in artificial sea water supplemented with 2% sodium chloride (ASW), incubated at room temperature and aeration under a 12-hour light-dark cycle for 48 hours. Infections were performed in 200 µL ASW containing 20-30 individual crustaceans and the 10^{7} CFU/mL of bacteria. After 48 hours incubation at room temperature, samples were washed twice in ASW to remove unattached bacteria and either homogenized for determining CIs (as above) or used directly for microscopy. **b)** _Cyanobacteria_. We modified previously established protocols as follows (69), *Microcystis aeruginosa* (UTEX LB 2385) were incubated in flasks containing BG-11 media (Gibco) under a 12-hour light-dark cycle with aeration (100 rpm) for about 14 days. *M. aeruginosa* cells were concentrated by centrifugation at

7500 rpm for 10 minutes, resuspended in BG-11 and co-cultured with 10^7 CFU/mL *V. cholerae* cells at a final OD700 of 0.3-0.4 in a 200 µL reaction volume. Samples were harvested at 24 hours and CIs were determined on LB-Sm-X-Gal plates, as above. **c)** *Mollusks*. Mucus-containing gills of the Eastern oyster *Crassostrea virginica* were harvested in 1x PBS pH 7.4 (1 g/mL), homogenized and autoclaved (34). M9 minimal media was supplemented with 0.1% sterile gill homogenate and inoculated with 50 µL of 10^7 CFU/mL of bacterial inoculum. Subsequently, cultures were incubated for 24 hours at 37 ˚C under aerated conditions. CIs were determined by plating appropriate dilutions on LB-Sm-X-Gal plates.

Phenotypic assays. **a)** Growth on LB. Overnight cultures were diluted to ~10^5 CFU/ml in fresh LB and 200 µL aliquots in flat-bottom 96-well plates (CytoOne) were monitored at OD595 nm, 37 ˚C for 24 hours in a Tecan Sunrise plate reader. **b)** Biofilm formation. These assays were performed as previously described with minor modifications(70). Briefly, overnight cultures in tryptone broth were diluted 1:500 in fresh broth and 200 µL aliquots in 96-well polystyrene plates (CytoOne) were incubated at 37 ˚C for 24 hours. After washing to remove unattached cells, biofilm cells were stained with 0.1% crystal violet and quantified by measuring OD595 after elution with 50% glacial acetic acid. **c)** Motility. These assays were performed in soft agar plates (LB-0.3% agar) as previously described (34). Diameter was measured for 12 hours every two hours.

Pangenome analysis. To evaluate the pangenome of *V. cholerae*, in a first approach dereplicated sequences were grouped in clusters at 98% identity using cd-hit (71). Then, pangenomes were calculated for each cluster individually using PPanGGOLiN (72) with default parameters. Ortholog proteins among the three groups (PCG, PCL, G3) and the rest of the groups were calculated using orthoMCL (73) (> 30 % amino acid identity, > 70 % alignment). A Venn diagram showing proteins shared and not shared among

groups was performed in R using the ggVennDiagram package (https://github.com/gaospecial/ggVennDiagram).

SUPPLEMENTARY FIGURES

Figure S1. **(A)** Map of Florida indicating the locations where the environmental *V. cholerae* strains were isolated (Shepard Park and Feller´s House). **(B)** Dendrogram representing the relatedness of *V. cholerae* isolates. The dendrogram is based on PCR fingerprints generated by VCR-PCR. Groups of similarity were established using the UPGMA method in BioNumerics v8.0. The red circles indicate clonal isolates that were selected for whole genome sequencing. **(C)** Pairwise comparison among the *V. cholerae* genomes. The pairwise comparison was obtained using the average nucleotide identity (ANI) from strains isolated in this study and the Pandemic Cholera Group (*V. cholerae* El Tor C6706). **(D)** Whole-genome SNP-based phylogenetic tree showing the relationships among the *V. cholerae* genomes obtained in this study and a representative strain from the Pandemic Cholera Group (highlighted with a red arrow).

Figure S2. Phylogenomic tree of dereplicated *V. cholerae* strains available in public databases and strains from this study. The tree was reconstructed from single nucleotide polymorphisms of the core genome. The branches of the members belonging to the same groupshave identical colour. The name of each branch indicates the reference genome of each non-clonal clade within that group. The position of the PCG is marked with a red arrow. Grey circles located at the branch nodes represent the bootstrap values.

Figure S3. **Genomic comparison of pathogenicity-related islands**. **(A)** Genomic comparison of PCG-like LPS clusters identified in *V. cholerae* environmental genomes. **(B)** Genomic comparison of similar CTXΦ phage found in dereplicated *V. cholerae* genomes. **(C)** Schematic representation CTXΦ phage found in *V. cholerae* 3528-08, both the numbers and the rectangles mark the different clusters acquired. Clusters of genes with a known function follow the same color pattern. **(D)** Comparison between the CTXΦ phage encoded by Pandemic Cholera Group strains (*V. cholerae* C6706) and *Vibrio mimicus* strain 2011V-1073. **(E)** Genomic comparison of homologous VPI-1 in

dereplicated *V. cholerae* genomes denote a reduced number of allelic variations in some PCG genes. **(F)** Genomic comparison of VPI-2 variants encoded in dereplicated *V. cholerae* genomes highlight pervasive modularity of the island. Clusters of genes with a known function follow the same color pattern. **(G)** Genomic comparison of homologous VSP-I across *V. cholerae* genomes. **(H)** Genomic comparison of VSP-II variants encoded in dereplicated *V. cholerae* genomes highlight pervasive modularity of the island. IS elements are highlighted in yellow.

Figure S4. Sequence alignment of the protein sequences of **(A)** TcpA **(B)** ToxT, **(C)** TcpF **(D)** HsdS and **(E)** VC_0176 from PCG and environmental strain.

Figure S5. Phenotypes of the *tcpF*^{IRL0081} and sextuple mutants. Isogenic *V. cholerae* C6706 strains encoding either the *tcpF*^{IRL0081} allele or the sextuple alleles were monitored for differences in **(A)** growth patterns in LB broth, **(B)** motility in 0.3% agar and **(C)** biofilm formation. n=6. *p*-values were determined using one-way ANOVA.

Figure S6. Establishment of the Artemia model of *V. cholerae* colonization. (A) CFU/ml of *V. cholerae* C6706 recovered post-infection at different time points and inoculum concentrations normalized to the input. Infection threshold (orange line) was determined by visualizing colonization using fluorescent microscopy. **(B)** RFP-tagged *V. cholerae* C6706 cannot colonize *A. salina* with an inoculum of 10^6 CFU/ml after 48 hours and fluorescence cannot be detected. **(C)** Inocula of 10^7 CFU/ml lead to consistent colonization of *A. salina* by RFP-tagged *V. cholerae* C6706 after 48 hours and red fluorescence can be detected.

Figure S7. **Pangenome dynamics and evolutionary trajectories of *V. cholerae* PCG. (A)** *Pangenome analysis*. The Venn diagram shows the numbers of unique and shared gene families between the pandemic cholera group (purple), pandemic cholera lineage

(blue), Group3 (light blue) and the rest of clades (grey). **(B)** *PCL evolution*. Phylogenomic tree of the pandemic cholera lineage. The colored circles represent the specific element as well as the percentage of completeness in each of the clades in reference to PCG. LPS, O1 lipopolysaccharide cluster; VPI-1, Vibrio pathogenicity island-1; VPI-2, Vibrio pathogenicity island-2; VSP-1, Vibrio seventh pandemic island-1; VSP-2, Vibrio seventh pandemic island-2.

SUPPLEMENTARY TABLES

Table S1. List of *Vibrio cholerae* collected in this study, together with genomic features and metadata.

Table S2. List of dereplicated (ANI>99%) *Vibrio cholerae* reference strains from the NCBI and presence of pathogenicity-related islands.

Table S3. Unique genes found in the PCG and acquired from PCL and G3.

Table S4. List of strains used in this study.

REFERENCES

1. S. Almagro-Moreno, R. K. Taylor, Cholera: Environmental Reservoirs and Impact on Disease Transmission. *Microbiology Spectrum* **1**, 149–165 (2013).

2. C. Lutz, M. Erken, P. Noorian, S. Sun, D. McDougald, Environmental reservoirs and mechanisms of persistence of Vibrio cholerae. *Frontiers in Microbiology* **4**, 375 (2013).

3. M. López-Pérez, *et al.*, Ecological diversification reveals routes of pathogen emergence in endemic Vibrio vulnificus populations. *Proc National Acad Sci* **118**, e2103470118 (2021).

4. B. Nandi, *et al.*, Rapid Method for Species-Specific Identification of Vibrio cholerae Using Primers Targeted to the Gene of Outer Membrane Protein OmpW. *J Clin Microbiol* **38**, 4145–4151 (2000).

5. C. S. J. Teh, K. L. Thong, R. Osawa, K. H. Chua, Comparative PCR-based fingerprinting of Vibrio cholerae isolated in Malaysia. *J Gen Appl Microbiol* **57**, 19–26 (2011).

6. J. F. Heidelberg, *et al.*, DNA sequence of both chromosomes of the cholera pathogen Vibrio cholerae. *Nature* **406**, 477–483 (2000).

7. M. López-Pérez, *et al.*, Evolutionary model of cluster divergence of the emergent marine pathogen Vibrio vulnificus: From genotype to ecotype. *mBio* **10**, 697 (2019).

8. S. N. Chatterjee, K. Chaudhuri, Lipopolysaccharides of Vibrio cholerae I. Physical and chemical characterization. *Biochimica Et Biophysica Acta Bba - Mol Basis Dis* **1639**, 65–79 (2003).

9. S. N. Chatterjee, K. Chaudhuri, Lipopolysaccharides of Vibrio cholerae II. Genetics of biosynthesis. *Biochimica Et Biophysica Acta Bba - Mol Basis Dis* **1690**, 93–109 (2004).

10. S. N. Chatterjee, K. Chaudhuri, Lipopolysaccharides of Vibrio cholerae: III. Biological functions. *Biochimica Et Biophysica Acta Bba - Mol Basis Dis* **1762**, 1–16 (2006).

11. C. A. Schnaitman, J. D. Klena, Genetics of lipopolysaccharide biosynthesis in enteric bacteria. *Microbiol Rev* **57**, 655–682 (1993).

12. M. J. Dorman, *et al.*, Genomics of the Argentinian cholera epidemic elucidate the contrasting dynamics of epidemic and endemic Vibrio cholerae. *Nat. Commun.* **11**, 4918 (2020).

13. T. K. Paisie, *et al.*, Molecular Basis of the Toxigenic Vibrio cholerae O1 Serotype Switch from Ogawa to Inaba in Haiti. *Microbiol. Spectr.* **11**, e03624-22 (2022).

14. W. Liang, *et al.*, Sequence polymorphisms of rfbT among the Vibrio cholerae O1 strains in the Ogawa and Inaba serotype shifts. *Bmc Microbiol* **13**, 173–173 (2013).

15. M. K. Waldor, J. J. Mekalanos, Lysogenic conversion by a filamentous phage encoding cholera toxin. *Science* **272**, 1910–1914 (1996).

16. J. B. Harris, R. C. LaRocque, F. Qadri, E. T. Ryan, S. B. Calderwood, Cholera. *Lancet* **379**, 2466–2476 (2012).

17. S. Kanungo, A. S. Azman, T. Ramamurthy, J. Deen, S. Dutta, Cholera. *Lancet* **399**, 1429–1440 (2022).

18. F. Rivera-Chávez, J. J. Mekalanos, Cholera toxin promotes pathogen acquisition of host-derived nutrients. *Nature* **572**, 244–248 (2019).

19. S. M. Faruque, J. J. Mekalanos, Phage-bacterial interactions in the evolution of toxigenic Vibrio cholerae. *Virulence* **3**, 556–565 (2014).

20. A. K. Mukhopadhyay, S. Chakraborty, Y. Takeda, G. B. Nair, D. E. Berg, Characterization of VPI pathogenicity island and CTXphi prophage in environmental strains of Vibrio cholerae. *The Journal of Bacteriology* **183**, 4737–4746 (2001).

21. L. Houot, *et al.*, Electrostatic interactions between the CTX phage minor coat protein and the bacterial host receptor TolA drive the pathogenic conversion of Vibrio cholerae. *J Biol Chem* **292**, 13584–13598 (2017).

22. F. Rodriguez-Valera, A.-B. Martin-Cuadrado, M. López-Pérez, Flexible genomic islands as drivers of genome evolution. *Curr Opin Microbiol* **31**, 154–160 (2016).

23. M. J. Dorman, *et al.*, High quality reference genomes for toxigenic and non-toxigenic Vibrio cholerae serogroup O139. *Sci Rep-uk* **9**, 5865 (2019).

24. R. K. Taylor, V. L. Miller, D. B. Furlong, J. J. Mekalanos, Use of phoA gene fusions to identify a pilus colonization factor coordinately regulated with cholera toxin. *Proc National Acad Sci* **84**, 2833–2837 (1987).

25. D. K. Karaolis, *et al.*, A Vibrio cholerae pathogenicity island associated with epidemic and pandemic strains. *Proceedings of the National Academy of Sciences of the United States of America* **95**, 3134–3139 (1998).

26. S. M. Faruque, J. J. Mekalanos, Pathogenicity islands and phages in Vibrio cholerae evolution. *Trends in microbiology* **11**, 505–510 (2003).

27. Y. A. O'Shea, E. F. Boyd, Mobilization of the Vibrio pathogenicity island between Vibrio cholerae isolates mediated by CP-T1 generalized transduction. *FEMS Microbiology Letters* **214**, 153–157 (2002).

28. R. A. Murphy, E. F. Boyd, Three pathogenicity islands of Vibrio cholerae can excise from the chromosome and form circular intermediates. *Journal of bacteriology* **190**, 636–647 (2008).

29. T. J. Kirn, N. Bose, R. K. Taylor, Secretion of a soluble colonization factor by the TCP type 4 pilus biogenesis pathway in Vibrio cholerae. *Molecular microbiology* **49**, 81–92 (2003).

30. T. J. Kirn, R. K. Taylor, TcpF is a soluble colonization factor and protective antigen secreted by El Tor and classical O1 and O139 Vibrio cholerae serogroups. *Infection and immunity* **73**, 4461–4470 (2005).

31. C. J. Megli, *et al.*, Crystal structure of the Vibrio cholerae colonization factor TcpF and identification of a functional immunogenic site. *Journal of molecular biology* **409**, 146–158 (2011).

32. W. S. Jermyn, E. F. Boyd, Characterization of a novel Vibrio pathogenicity island (VPI-2) encoding neuraminidase (nanH) among toxigenic Vibrio cholerae isolates. *Microbiology* **148**, 3681–3693 (2002).

33. S. Almagro-Moreno, E. F. Boyd, Sialic acid catabolism confers a competitive advantage to pathogenic Vibrio cholerae in the mouse intestine. *Infection and immunity* **77**, 3807–3816 (2009).

34. G. Reddi, K. Pruss, R. K. Taylor, K. L. Cottingham, S. Almagro-Moreno, Catabolism of mucus components influences motility of Vibrio cholerae in the presence of environmental reservoirs. *PLoS ONE* **13**, e0201383 (2018).

35. S. Almagro-Moreno, M. G. Napolitano, E. F. Boyd, Excision dynamics of Vibrio pathogenicity island-2 from Vibrio cholerae: role of a recombination directionality factor VefA. *BMC microbiology* **10**, 306 (2010).

36. M. R. Carpenter, S. Rozovsky, E. F. Boyd, V. J. DiRita, Pathogenicity Island Cross Talk Mediated by Recombination Directionality Factors Facilitates Excision from the Chromosome. *The Journal of Bacteriology* **198**, 766–776 (2016).

37. C. J. Grim, *et al.*, Occurrence of the Vibrio cholerae Seventh Pandemic VSP-I Island and a New Variant. *OMICS: A Journal of Integrative Biology* **14**, 1–7 (2010).

38. B. W. Davies, R. W. Bogard, T. S. Young, J. J. Mekalanos, Coordinated regulation of accessory genetic elements produces cyclic di-nucleotides for V. cholerae virulence. *Cell* **149**, 358–370 (2012).

39. Y. A. O'Shea, *et al.*, The Vibrio seventh pandemic island-II is a 26.9 kb genomic island present in Vibrio cholerae El Tor and O139 serogroup isolates that shows homology to a 43.4 kb genomic island in V. vulnificus. *Microbiology* **150**, 4053–4063 (2004).

40. E. Taviani, *et al.*, Discovery of novel Vibrio cholerae VSP-II genomic islands using comparative genomic analysis. *FEMS Microbiology Letters* **308**, 130–137 (2010).

41. B. J. Haley, *et al.*, Genomic and Phenotypic Characterization of Vibrio cholerae Non-O1 Isolates from a US Gulf Coast Cholera Outbreak. *PLoS ONE* **9**, e86264 (2014).

42. S. Nusrin, *et al.*, Peruvian Vibrio cholerae O1 El Tor strains possess a distinct region in the Vibrio seventh pandemic island-II that differentiates them from the prototype seventh pandemic El Tor strains. *J Med Microbiol* **58**, 342–354 (2009).

43. A. Huq, *et al.*, Detection, Isolation, and Identification of Vibrio cholerae from the Environment. *Curr Protoc Microbiol* **26**, 6A.5.1-6A.5.51 (2012).

44. A. Naha, *et al.*, Development and Evaluation of a PCR Assay for Tracking the Emergence and Dissemination of Haitian Variant ctxB in Vibrio cholerae O1 Strains Isolated from Kolkata, India. *J Clin Microbiol* **50**, 1733–1736 (2012).

45. W. Zou, *et al.*, Evaluation of Pulsed-Field Gel Electrophoresis Profiles for Identification of Salmonella Serotypes. *J Clin Microbiol* **48**, 3122–3126 (2010).

46. A. M. Bolger, M. Lohse, B. Usadel, Trimmomatic: a flexible trimmer for Illumina sequence data. *Bioinformatics* **30**, 2114–2120 (2014).

47. A. Bankevich, *et al.*, SPAdes: A New Genome Assembly Algorithm and Its Applications to Single-Cell Sequencing. *Journal of Computational Biology* **19**, 455–477 (2012).

48. D. Hyatt, *et al.*, Prodigal: prokaryotic gene recognition and translation initiation site identification. *BMC Bioinformatics* **11**, 1–11 (2010).

49. T. M. Lowe, S. R. Eddy, tRNAscan-SE: a program for improved detection of transfer RNA genes in genomic sequence. *Nucleic acids research* **25**, 955–964 (1997).

50. Y. Huang, P. Gilna, W. Li, Identification of ribosomal RNA genes in metagenomic fragments. *Bioinformatics* **25**, 1338–1340 (2009).

51. B. Buchfink, C. Xie, D. H. Huson, Fast and sensitive protein alignment using DIAMOND. *Nature methods* **12**, 59–60 (2015).

52. S. R. Eddy, Accelerated Profile HMM Searches. *PLOS Computational Biology* **7**, e1002195 (2011).

53. R. L. Tatusov, *et al.*, The COG database: new developments in phylogenetic classification of proteins from complete genomes. *Nucleic acids research* **29**, 22–28 (2001).

54. D. H. Haft, *et al.*, TIGRFAMs: a protein family resource for the functional identification of proteins. *Nucleic acids research* **29**, 41–43 (2001).

55. M. R. Olm, C. T. Brown, B. Brooks, J. F. Banfield, dRep: a tool for fast and accurate genomic comparisons that enables improved genome recovery from metagenomes through de-replication. *Isme J* **11**, 2864–2868 (2017).

56. D. H. Parks, M. Imelfort, C. T. Skennerton, P. Hugenholtz, G. W. Tyson, CheckM: assessing the quality of microbial genomes recovered from isolates, single cells, and metagenomes. *Genome Research* **25**, 1043–1055 (2015).

57. M. Shakya, *et al.*, Standardized phylogenetic and molecular evolutionary analysis applied to species across the microbial tree of life. *Sci Rep-uk* **10**, 1723 (2020).

58. L.-T. Nguyen, H. A. Schmidt, A. von Haeseler, B. Q. Minh, IQ-TREE: A Fast and Effective Stochastic Algorithm for Estimating Maximum-Likelihood Phylogenies. *Mol Biol Evol* **32**, 268–274 (2015).

59. B. J. Grant, A. P. C. Rodrigues, K. M. ElSawy, J. A. McCammon, L. S. D. Caves, Bio3d: an R package for the comparative analysis of protein structures. *Bioinformatics* **22**, 2695–2696 (2006).

60. S. Lê, J. Josse, F. Husson, FactoMineR : An R Package for Multivariate Analysis. *J. Stat. Softw.* **25** (2008).

61. S. F. Altschul, W. Gish, W. Miller, E. W. Myers, D. J. Lipman, Basic local alignment search tool. *J Mol Biol* **215**, 403–410 (1990).

62. S. Altschul, Gapped BLAST and PSI-BLAST: a new generation of protein database search programs. *Nucleic acids research* **25**, 3389–3402 (1997).

63. B. J. Shapiro, I. Levade, G. Kovacikova, R. K. Taylor, S. Almagro-Moreno, Origins of pandemic Vibrio cholerae from environmental gene pools. *Nature Microbiology* **2**, 16240 (2017).

64. K. Skorupski, R. K. Taylor, Positive selection vectors for allelic exchange. *Gene* **169**, 47–52 (1996).

65. K.-H. Choi, H. P. Schweizer, mini-Tn7 insertion in bacteria with single attTn7 sites: example Pseudomonas aeruginosa. *Nat. Protoc.* **1**, 153–161 (2006).

66. Y. A. Millet, *et al.*, Insights into Vibrio cholerae intestinal colonization from monitoring fluorescently labeled bacteria. *PLoS pathogens* **10**, e1004405 (2014).

67. J. S. Matson, Vibrio Cholerae, Methods and Protocols. *Methods Mol. Biol.* **1839**, 147–152 (2018).

68. M.-N. Lee, S.-K. Kim, X.-H. Li, J.-H. Lee, Bacterial virulence analysis using brine shrimp as an infection model in relation to the importance of quorum sensing and proteases. *J Gen Appl Microbiol* **60**, 169–174 (2014).

69. M. S. Islam, *et al.*, Role of cyanobacteria in the persistence of Vibrio choleraeO139 in saline microcosms. *Canadian journal of microbiology* **50**, 127–131 (2004).

70. G. A. O'Toole, Microtiter Dish Biofilm Formation Assay. *Journal of visualized experiments : JoVE* (2011). https://doi.org/10.3791/2437.

71. L. Fu, B. Niu, Z. Zhu, S. Wu, W. Li, CD-HIT: accelerated for clustering the next-generation sequencing data. *Bioinformatics* **28**, 3150–3152 (2012).

72. G. Gautreau, *et al.*, PPanGGOLiN: Depicting microbial diversity via a partitioned pangenome graph. *Plos Comput Biol* **16**, e1007732 (2020).

73. L. Li, C. J. Stoeckert, D. S. Roos, OrthoMCL: identification of ortholog groups for eukaryotic genomes. *Genome Research* **13**, 2178–2189 (2003).

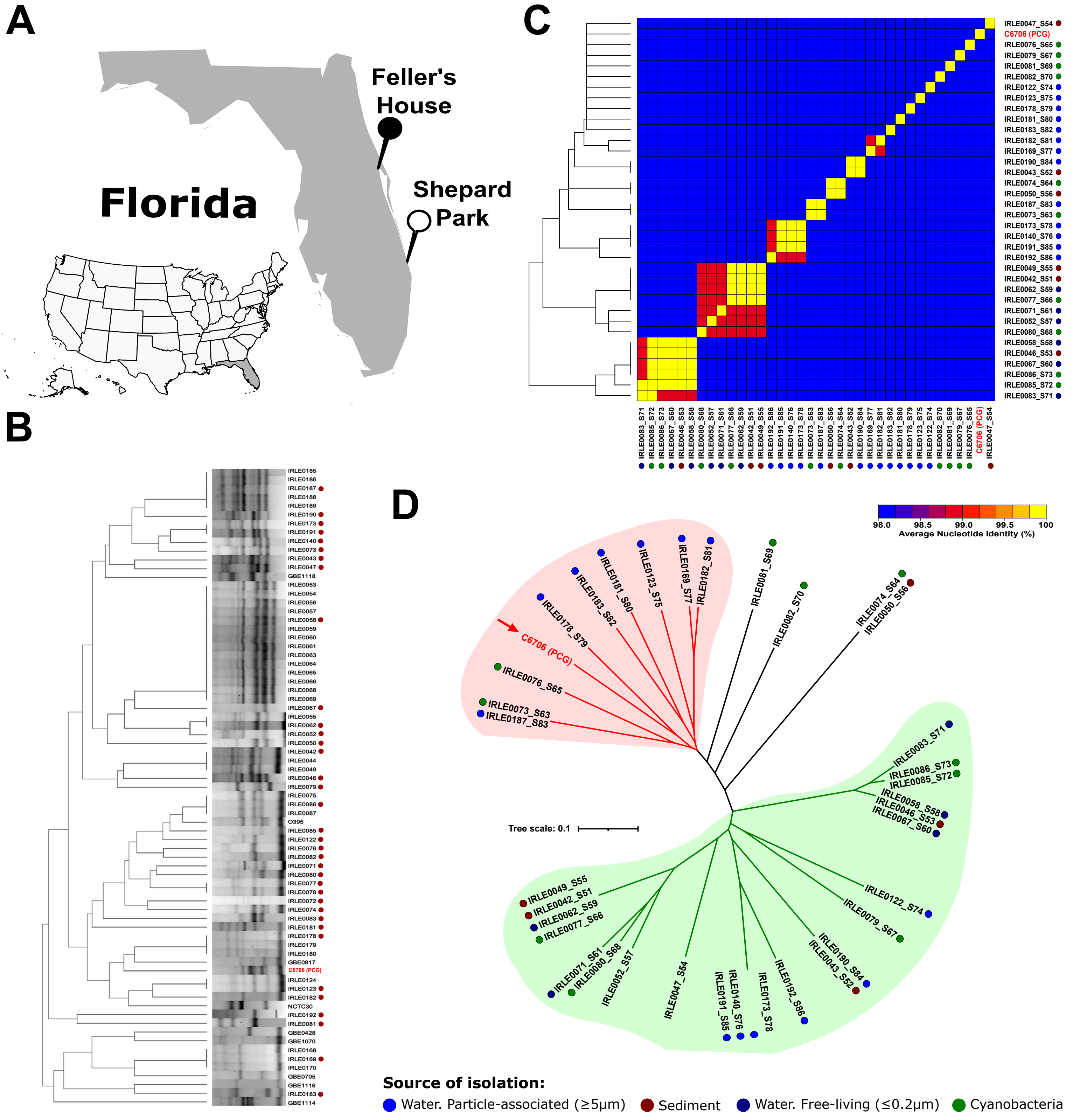

A
Feller's House
Shepard Park
Florida
B
C
IRLE0047_S54
C6706 (PCG)
IRLE0076_S65
IRLE0079_S67
IRLE0081_S69
IRLE0082_S70
IRLE0122_S74
IRLE0123_S75
IRLE0178_S79
IRLE0181_S80
IRLE0183_S82
IRLE0182_S81
IRLE0169_S77
IRLE0190_S84
IRLE0043_S52
IRLE0074_S64
IRLE0050_S56
IRLE0187_S83
IRLE0073_S63
IRLE0173_S78
IRLE0140_S76
IRLE0191_S85
IRLE0192_S86
IRLE0049_S55
IRLE0042_S51
IRLE0062_S59
IRLE0077_S66
IRLE0071_S61
IRLE0052_S57
IRLE0080_S68
IRLE0058_S58
IRLE0046_S53
IRLE0067_S60
IRLE0086_S73
IRLE0085_S72
IRLE0083_S71
D
98.0
98.5
99.0
99.5
100
Average Nucleotide Identity (%)
Tree scale: 0.1
Source of isolation:
Water. Particle-associated (≥5μm)
Sediment
Water. Free-living (≤0.2μm)
Cyanobacteria

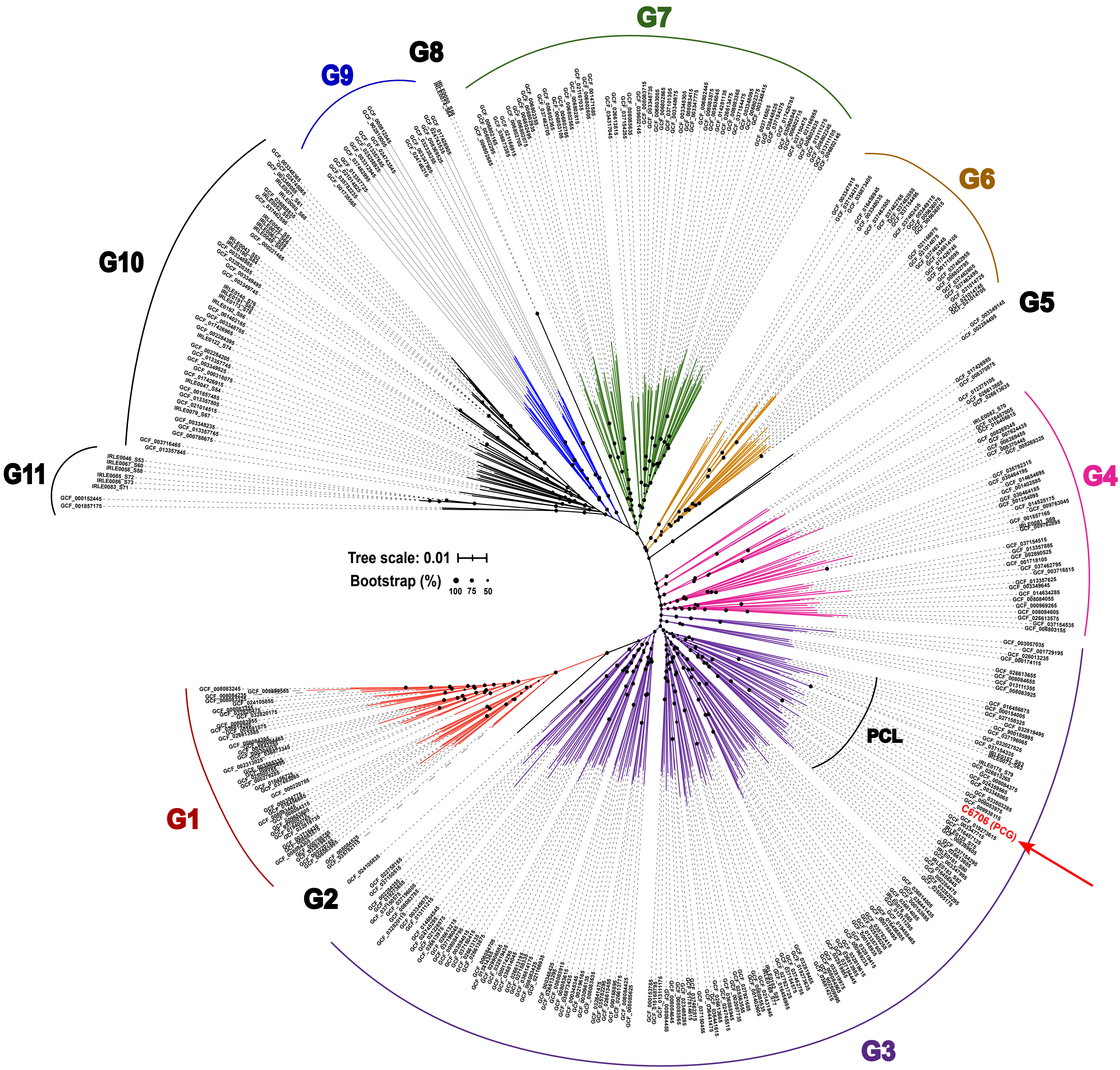

G7
G8
G9
G10
G11
G6
G5
G4
G3
G2
G1
PCL
C6706 (PCG)
Tree scale: 0.01
Bootstrap (%)
100
75
50

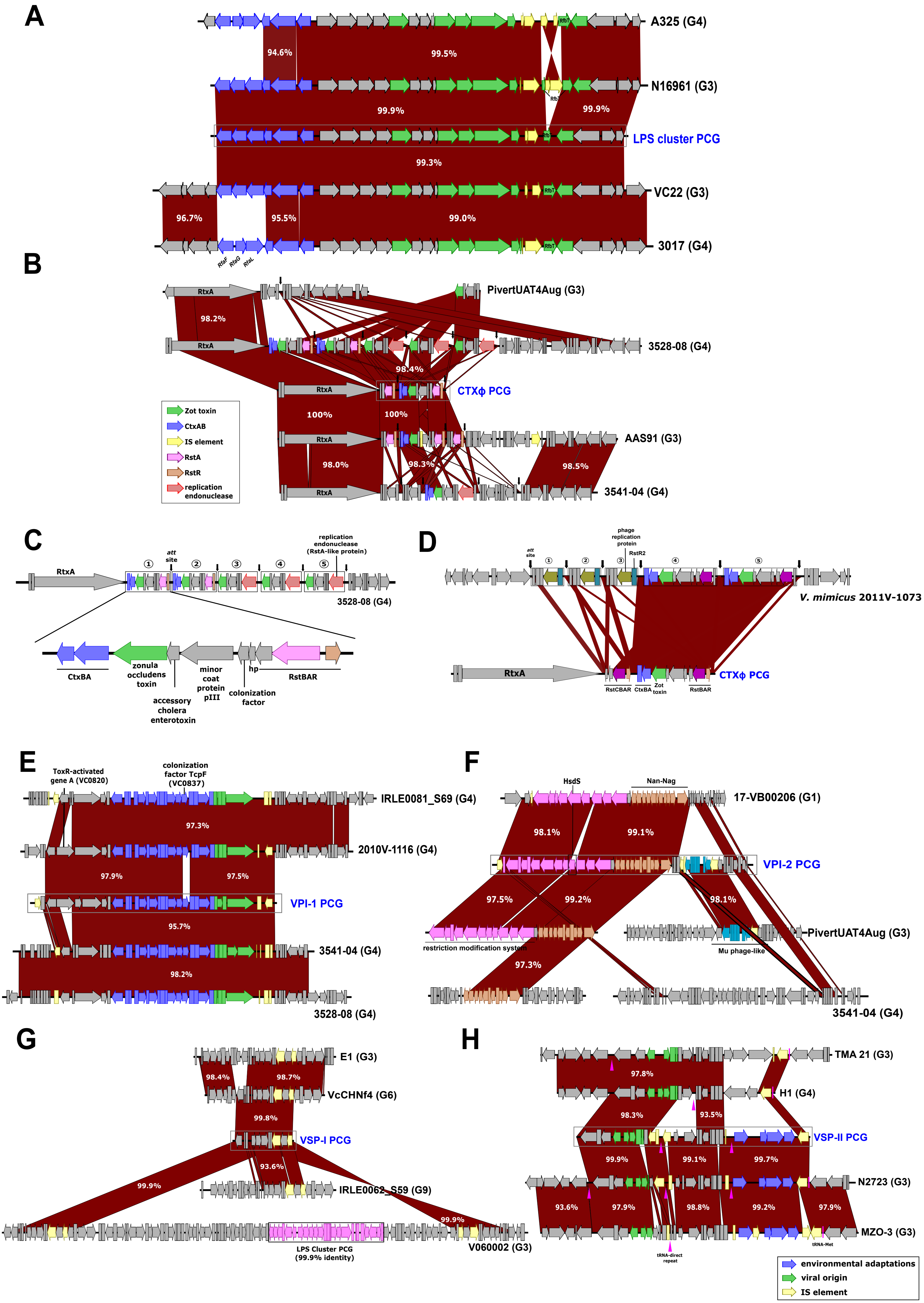

A
A325 (G4)
94.6%
99.5%
N16961 (G3)
99.9%
99.9%
LPS cluster PCG
99.3%
VC22 (G3)
96.7%
95.5%
99.0%
3017 (G4)
RfaF
RfaG
RfaL
B
RtxA
PivertUAT4Aug (G3)
98.2%
3528-08 (G4)
98.4%
CTXϕ PCG
100%
100%
AAS91 (G3)
98.0%
98.3%
98.5%
3541-04 (G4)
Zot toxin
CtxAB
IS element
RstA
RstR
replication endonuclease
C
att site
replication endonuclease (RstA-like protein)
3528-08 (G4)
CtxBA
zonula occludens toxin
accessory cholera enterotoxin
minor coat protein pIII
hp
colonization factor
RstBAR
D
phage replication protein
RstR2
V. mimicus 2011V-1073
CTXϕ PCG
RstCBAR
CtxBA
Zot toxin
RstBAR
E
ToxR-activated gene A (VC0820)
colonization factor TcpF (VC0837)
IRLE0081_S69 (G4)
97.3%
2010V-1116 (G4)
97.9%
97.5%
VPI-1 PCG
95.7%
3541-04 (G4)
98.2%
3528-08 (G4)
F
HsdS
Nan-Nag
17-VB00206 (G1)
98.1%
99.1%
VPI-2 PCG
97.5%
99.2%
98.1%
PivertUAT4Aug (G3)
restriction modification system
Mu phage-like
97.3%
3541-04 (G4)
G
E1 (G3)
98.4%
98.7%
VcCHNf4 (G6)
99.8%
VSP-I PCG
93.6%
99.9%
IRLE0062_S59 (G9)
99.9%
LPS Cluster PCG (99.9% identity)
V060002 (G3)
H
TMA 21 (G3)
97.8%
H1 (G4)
98.3%
93.5%
VSP-II PCG
99.9%
99.1%
99.7%
N2723 (G3)
93.6%
97.9%
98.8%
99.2%
97.9%
MZO-3 (G3)
tRNA-direct repeat
tRNA-Met
environmental adaptations
viral origin
IS element

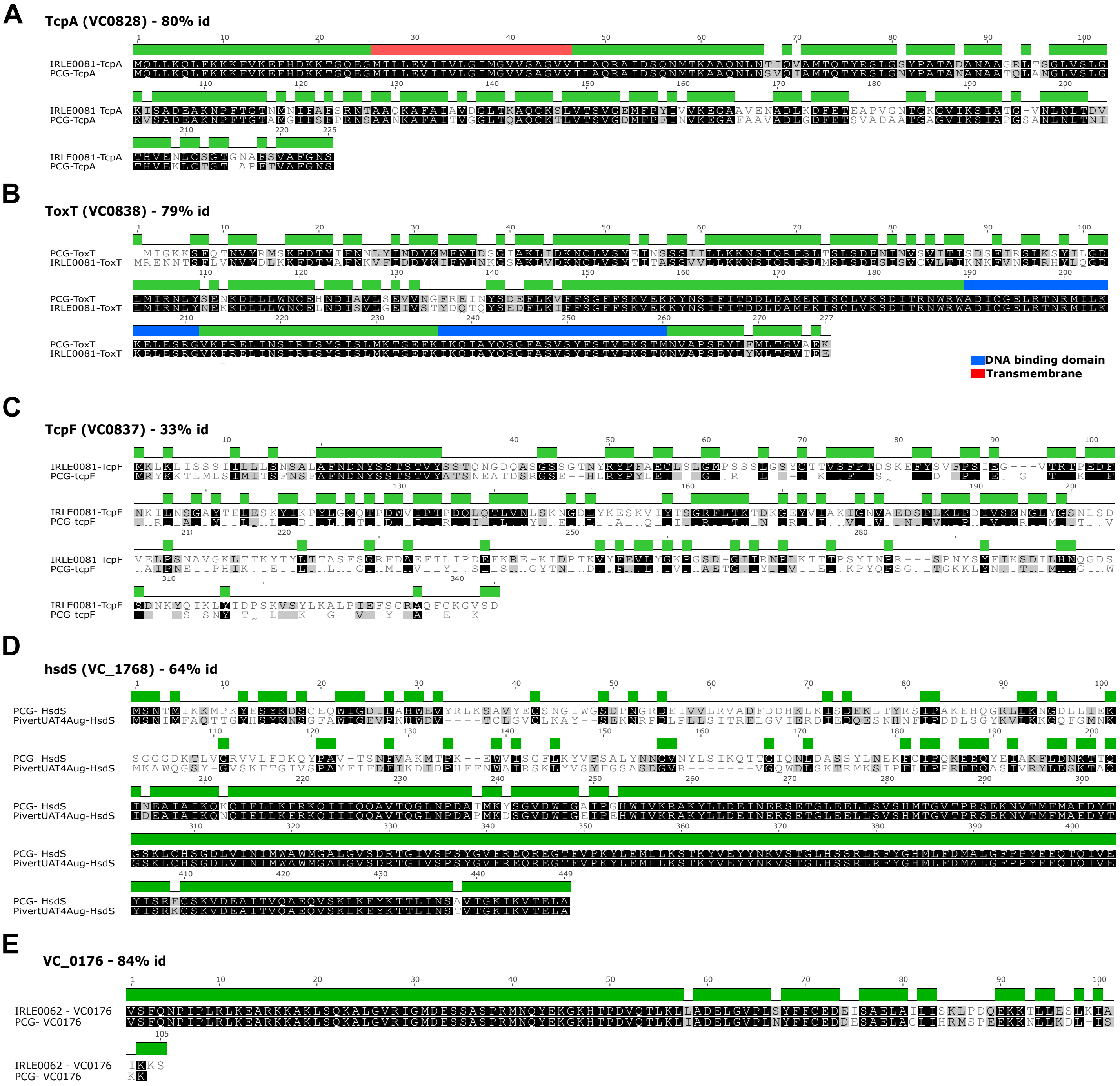
A
TcpA (VC0828) - 80% id
IRLE0081-TcpA
PCG-TcpA
B
ToxT (VC0838) - 79% id
PCG-ToxT
IRLE0081-ToxT
DNA binding domain
Transmembrane
C
TcpF (VC0837) - 33% id
IRLE0081-TcpF
PCG-tcpF
D
hsdS (VC_1768) - 64% id
PCG- HsdS
PivertUAT4Aug-HsdS
E
VC_0176 - 84% id
IRLE0062 - VC0176
PCG- VC0176

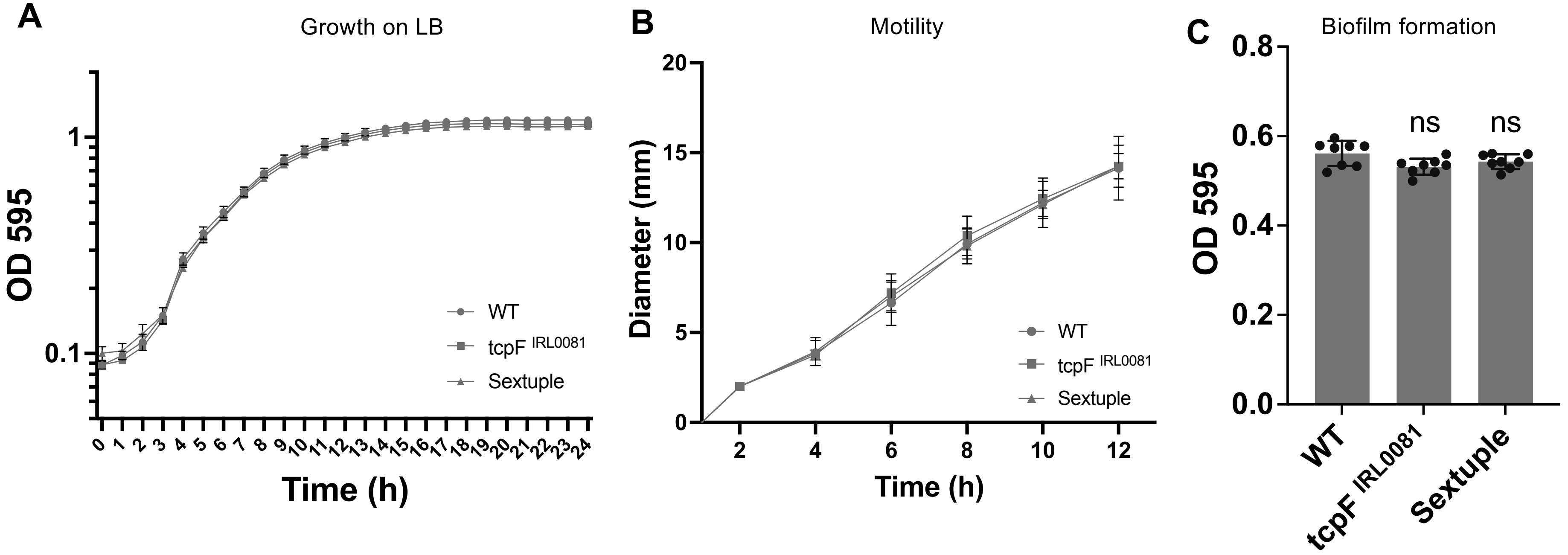
A
Growth on LB
OD 595
1
0.1
WT
tcpF IRL0081
Sextuple
0 1 2 3 4 5 6 7 8 9 10 11 12 13 14 15 16 17 18 19 20 21 22 23 24
Time (h)
B
Motility
Diameter (mm)
20
15
10
5
0
2 4 6 8 10 12
Time (h)
WT
tcpF IRL0081
Sextuple
C
Biofilm formation
OD 595
0.8
0.6
0.4
0.2
0.0
ns
ns
WT
tcpF IRL0081
Sextuple

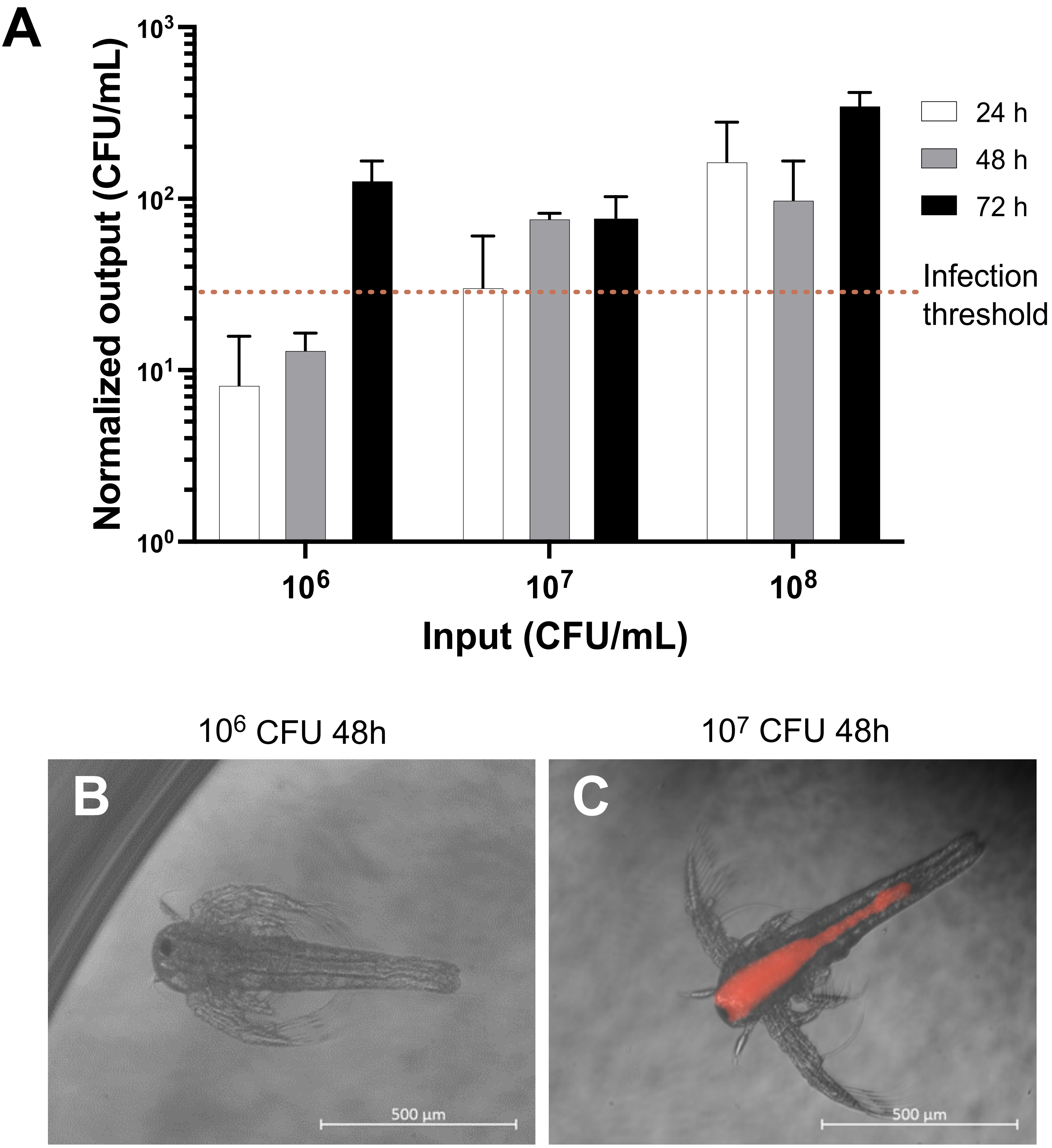

A
Normalized output (CFU/mL)
10^3
10^2
10^1
10^0
24 h
48 h
72 h
Infection threshold
10^6
10^7
10^8
Input (CFU/mL)
10^6 CFU 48h
10^7 CFU 48h
B
C
500 µm
500 µm

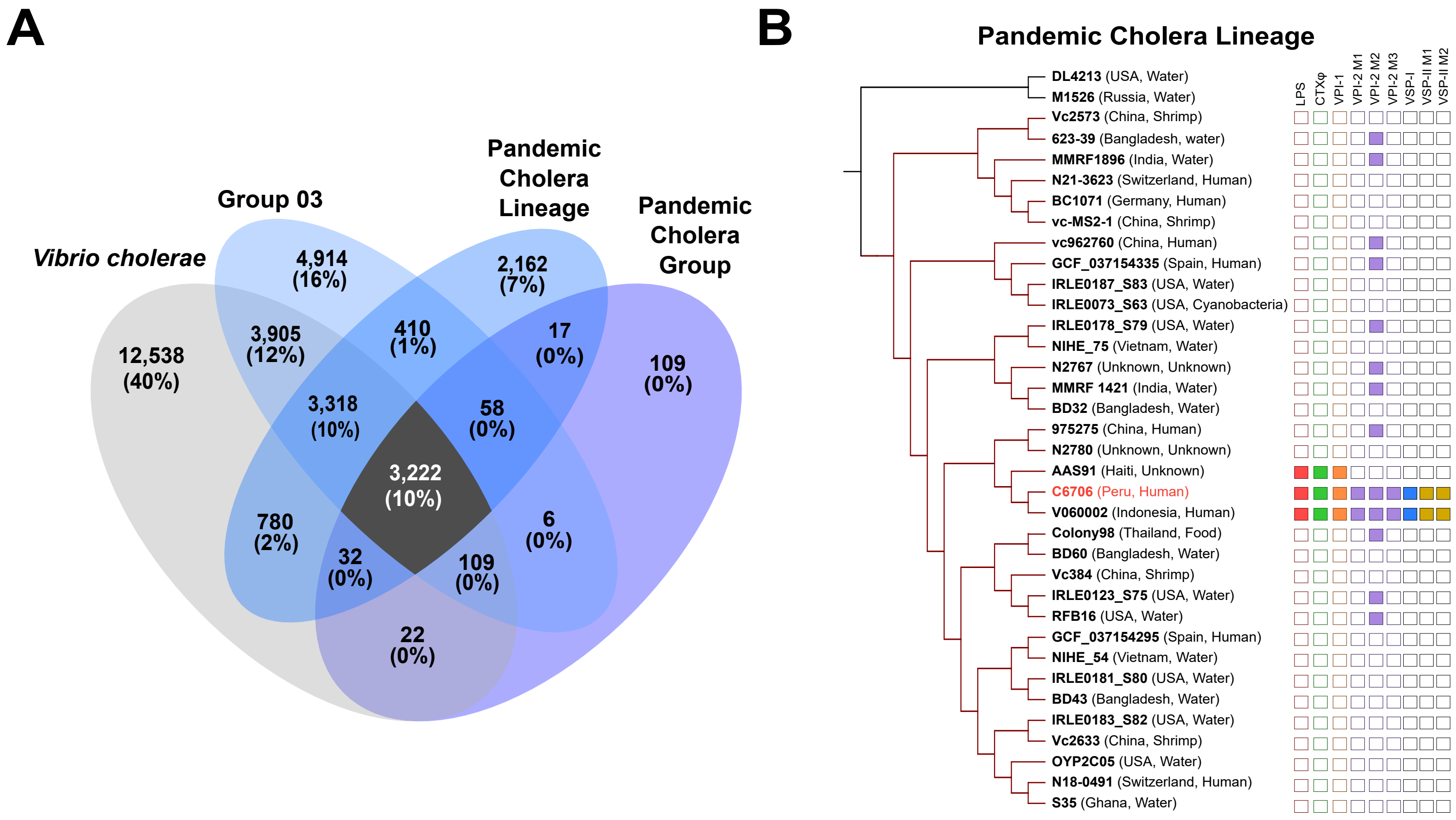
A
Vibrio cholerae
Group 03
Pandemic Cholera Lineage
Pandemic Cholera Group
12,538 (40%)
4,914 (16%)
2,162 (7%)
109 (0%)
3,905 (12%)
410 (1%)
17 (0%)
3,318 (10%)
58 (0%)
3,222 (10%)
780 (2%)
6 (0%)
32 (0%)
109 (0%)
22 (0%)
B
Pandemic Cholera Lineage
LPS
CTXφ
VPI-1
VPI-2 M1
VPI-2 M2
VPI-2 M3
VSP-I
VSP-II M1
VSP-II M2
DL4213 (USA, Water)
M1526 (Russia, Water)
Vc2573 (China, Shrimp)
623-39 (Bangladesh, water)
MMRF1896 (India, Water)
N21-3623 (Switzerland, Human)
BC1071 (Germany, Human)
vc-MS2-1 (China, Shrimp)
vc962760 (China, Human)
GCF_037154335 (Spain, Human)
IRLE0187_S83 (USA, Water)
IRLE0073_S63 (USA, Cyanobacteria)
IRLE0178_S79 (USA, Water)
NIHE_75 (Vietnam, Water)
N2767 (Unknown, Unknown)
MMRF 1421 (India, Water)
BD32 (Bangladesh, Water)
975275 (China, Human)
N2780 (Unknown, Unknown)
AAS91 (Haiti, Unknown)
C6706 (Peru, Human)
V060002 (Indonesia, Human)
Colony98 (Thailand, Food)
BD60 (Bangladesh, Water)
Vc384 (China, Shrimp)
IRLE0123_S75 (USA, Water)
RFB16 (USA, Water)
GCF_037154295 (Spain, Human)
NIHE_54 (Vietnam, Water)
IRLE0181_S80 (USA, Water)
BD43 (Bangladesh, Water)
IRLE0183_S82 (USA, Water)
Vc2633 (China, Shrimp)
OYP2C05 (USA, Water)
N18-0491 (Switzerland, Human)
S35 (Ghana, Water)

Table S1. List of *Vibrio cholerae* collected in this study, together with genomic features and metadata

Organism Name	Strain	#Contigs	Genome length (bp)	GC content (%)	# of protein coding genes	Location	Month of Isolation	Source	Fraction
Vibrio cholerae	IRLE0042	181	3,868,465	47.7	3,491	Shepards Park	Sept, 2018	Sediment	-
Vibrio cholerae	IRLE0043	227	3,915,606	47.4	3,575	Shepards Park	Sept, 2018	Sediment	-
Vibrio cholerae	IRLE0046	595	4,051,774	47.4	3,930	Shepards Park	Sept, 2018	Sediment	-
Vibrio cholerae	IRLE0047	373	3,929,895	47.7	3,666	Shepards Park	Sept, 2018	Sediment	-
Vibrio cholerae	IRLE0049	155	3,868,094	47.6	3,463	Shepards Park	Sept, 2018	Sediment	-
Vibrio cholerae	IRLE0050	503	3,847,763	48.0	3,726	Shepards Park	Sept, 2018	Sediment	-
Vibrio cholerae	IRLE0052	242	3,936,616	47.5	3,609	Shepards Park	Sept, 2018	Water	Particle-associated (≥5μm)
Vibrio cholerae	IRLE0058	564	4,051,230	47.4	3,897	Shepards Park	Sept, 2018	Water	Particle-associated (≥5μm)
Vibrio cholerae	IRLE0062	151	3,868,458	47.7	3,458	Shepards Park	Sept, 2018	Water	Particle-associated (≥5μm)
Vibrio cholerae	IRLE0067	508	4,049,234	47.4	3,848	Shepards Park	Sept, 2018	Water	Particle-associated (≥5μm)
Vibrio cholerae	IRLE0071	326	3,968,968	47.2	3,671	Shepards Park	Sept, 2018	Water	Particle-associated (≥5μm)
Vibrio cholerae	IRLE0073	319	3,939,905	47.3	3,646	Shepards Park	Sept, 2018	Cyanobacteria	-
Vibrio cholerae	IRLE0074	453	3,983,007	47.1	3,883	Shepards Park	Sept, 2018	Cyanobacteria	-
Vibrio cholerae	IRLE0076	227	3,938,718	48.7	3,616	Shepards Park	Sept, 2018	Cyanobacteria	-
Vibrio cholerae	IRLE0077	149	3,868,582	47.7	3,457	Shepards Park	Sept, 2018	Cyanobacteria	-
Vibrio cholerae	IRLE0079	268	3,915,255	47.7	3,623	Shepards Park	Sept, 2018	Cyanobacteria	-
Vibrio cholerae	IRLE0080	191	3,944,917	47.5	3,566	Shepards Park	Sept, 2018	Cyanobacteria	-
Vibrio cholerae	IRLE0081	166	3,922,461	47.7	3,535	Shepards Park	Sept, 2018	Cyanobacteria	-
Vibrio cholerae	IRLE0082	443	4,064,950	47.7	3,865	Shepards Park	Sept, 2018	Cyanobacteria	-
Vibrio cholerae	IRLE0083	713	4,070,289	47.5	3,935	Shepards Park	Sept, 2018	Water	Free-living (≤0.2μm)
Vibrio cholerae	IRLE0085	598	4,041,698	47.4	3,890	Shepards Park	Sept, 2018	Cyanobacteria	-
Vibrio cholerae	IRLE0086	623	4,039,358	47.4	3,898	Shepards Park	Sept, 2018	Cyanobacteria	-
Vibrio cholerae	IRLE0122	210	4,024,379	47.7	3,671	Shepards Park	Jun, 2019	Water	Particle-associated (≥5μm)
Vibrio cholerae	IRLE0123	276	4,026,501	47.4	3,766	Shepards Park	Jun, 2019	Water	Particle-associated (≥5μm)
Vibrio cholerae	IRLE0140	389	3,961,777	47.5	3,777	Shepards Park	Jun, 2019	Water	Free-living (≤0.2μm)
Vibrio cholerae	IRLE0169	320	3,919,979	47.7	3,662	Shepards Park	Jul, 2019	Water	Particle-associated (≥5μm)
Vibrio cholerae	IRLE0173	231	3,959,117	47.7	3,597	Shepards Park	Jul, 2019	Water	Free-living (≤0.2μm)
Vibrio cholerae	IRLE0178	312	3,992,006	46.4	3,723	Shepards Park	Jul, 2019	Water	Particle-associated (≥5μm)
Vibrio cholerae	IRLE0181	204	3,904,104	47.6	3,572	Shepards Park	Jul, 2019	Water	Free-living (≤0.2μm)
Vibrio cholerae	IRLE0182	243	3,892,734	47.6	3,550	Shepards Park	Jul, 2019	Water	Free-living (≤0.2μm)
Vibrio cholerae	IRLE0183	210	4,115,845	47.5	3,824	Shepards Park	Jul, 2019	Water	Free-living (≤0.2μm)
Vibrio cholerae	IRLE0187	318	3,938,072	47.6	3,645	Shepards Park	Aug, 2019	Water	Particle-associated (≥5μm)
Vibrio cholerae	IRLE0190	212	3,916,639	47.3	3,576	Shepards Park	Aug, 2019	Water	Free-living (≤0.2μm)
Vibrio cholerae	IRLE0191	231	3,957,203	47.5	3,597	Shepards Park	Aug, 2019	Water	Free-living (≤0.2μm)
Vibrio cholerae	IRLE0192	210	3,905,334	47.6	3,548	Shepards Park	Aug, 2019	Water	Free-living (≤0.2μm)

Table S2. List of dereplicated (ANI>99%) *Vibrio cholerae* reference including IRL strains

Species	Genome ID	NCBI name	Isolated source	Location	Group	PCL	LPS	CTX	VPI-1	VPI-2 M1	VPI-2 M2	VPI-2 M3	VSP-I	VSP-II M1	VSP-II M2 (N16961)	VSP-II M2 (C6760)
Vibrio cholerae	GCF_019400765	458_01	Sewer	Brazil	G1			x								x
Vibrio cholerae	GCF_035782115	R18275	Environment	Brazil	G1											x
Vibrio cholerae	GCF_024105855	PGB-VCH005	Water	Algeria	G1					x	x			x		
Vibrio cholerae	GCF_003591575	FORC_073	Human	South Korea	G1						x		x			
Vibrio cholerae	GCF_008083995	N2777	Unknown	Unknown	G1							x				
Vibrio cholerae	GCF_008085495	N2749	Unknown	Unknown	G1					x	x					
Vibrio cholerae	GCF_036814205	17-VB00206	Shrimps	Germany	G1					x	x					
Vibrio cholerae	GCF_000220765	HE39	Unknown	Unknown	G1						x					
Vibrio cholerae	GCF_000279285	HE-45	Unknown	Haiti	G1						x					
Vibrio cholerae	GCF_003585335	4119	Water	Bangladesh	G1						x					
Vibrio cholerae	GCF_008083355	N2818	Unknown	Unknown	G1						x					
Vibrio cholerae	GCF_008084395	N2764	Unknown	Unknown	G1						x					
Vibrio cholerae	GCF_009653605	Santiago	Human	Santiago, Chile	G1						x					
Vibrio cholerae	GCF_016456685	Vc3023	Animal	China	G1						x					
Vibrio cholerae	GCF_025505155	E19	Water	Ghana	G1						x					
Vibrio cholerae	GCF_032820175	N18-1318	Homo sapiens	Switzerland	G1						x					
Vibrio cholerae	GCF_000304915	CP1035(8)	Human	Mexico	G1		x									
Vibrio cholerae	GCF_000220785	HE48	Environment	Haiti	G1											
Vibrio cholerae	GCF_000304775	HC-1A2	Human	Haiti	G1											
Vibrio cholerae	GCF_000788755	2012Env-92	Environment	Haiti	G1											
Vibrio cholerae	GCF_002313025	FORC_055	Fish	South Korea	G1											
Vibrio cholerae	GCF_002843255	VcN1	Water	Bangladesh	G1											
Vibrio cholerae	GCF_003716435	2011EL-1271	Unknown	Haiti	G1											
Vibrio cholerae	GCF_008083245	N2829	Unknown	Unknown	G1											
Vibrio cholerae	GCF_008083325	N2822	Unknown	Unknown	G1											
Vibrio cholerae	GCF_008083445	N2813	Unknown	Unknown	G1											
Vibrio cholerae	GCF_008083575	N2808	Unknown	Unknown	G1											
Vibrio cholerae	GCF_008083955	N2779	Unknown	Unknown	G1											
Vibrio cholerae	GCF_008084235	N2775	Unknown	Unknown	G1											
Vibrio cholerae	GCF_008084305	N2769	Unknown	Unknown	G1											
Vibrio cholerae	GCF_008084315	N2771	Unknown	Unknown	G1											
Vibrio cholerae	GCF_008084465	N2761	Unknown	Unknown	G1											
Vibrio cholerae	GCF_008084525	N2759	Unknown	Unknown	G1											
Vibrio cholerae	GCF_009899585	1376_01	Sewage	Brazil	G1											
Vibrio cholerae	GCF_016456725	Vc382	Animal	China	G1											
Vibrio cholerae	GCF_026613585	NIHE_60	Environment	Vietnam	G1											
Vibrio cholerae	GCF_032819675	N19-2759	Homo sapiens	Switzerland	G1											
Vibrio cholerae	GCF_032819735	N19-1763	Homo sapiens	Switzerland	G1											
Vibrio cholerae	GCF_032820475	N17-0919	Homo sapiens	Switzerland	G1											
Vibrio cholerae	GCF_036873345	17-VB00001	Shrimps	Germany (Baltic Sea)	G1											
Vibrio cholerae	GCF_037483065	A3616	Homo sapiens	China	G1											
Vibrio cholerae	GCF_937000115	CNRVC190247	Homo sapiens	Yemen	G1											
Vibrio cholerae	GCF_002284205	OYP6D06	Water	USA	G10								x			
Vibrio cholerae	GCF_032335255	22-VB00037-2	Food	Germany	G10								x			
Vibrio cholerae	GCF_017426905	DL4212	Water	USA	G10							x				
Vibrio cholerae	GCF_013357725	SL6Y	Water	USA	G10						x					
Vibrio cholerae	GCF_013357745	SP6G	Water	USA	G10						x					
Vibrio cholerae	GCF_024743535	RM-160-1	Mollusks	Canada	G10						x					
Vibrio cholerae	GCF_037462995	18-VB00108	water	Germany (Baltic Sea)	G10						x					
Vibrio cholerae	GCF_000318075	PS15	Sediment	USA	G10											
Vibrio cholerae	GCF_000788675	2012Env-32	Environment	Haiti	G10											
Vibrio cholerae	GCF_000812045	ZWU0020	Fish	USA	G10											
Vibrio cholerae	GCF_001402185	YB1A01	Water	USA	G10											
Vibrio cholerae	GCF_001735565	S12	Water	Australia	G10											
Vibrio cholerae	GCF_001857485	TP	Water	USA	G10											
Vibrio cholerae	GCF_002284395	OYP2A12	Water	USA	G10											
Vibrio cholerae	GCF_003312945	09_113	Human	Brazil	G10											
Vibrio cholerae	GCF_003347905	BD47	Water	Bangladesh	G10											
Vibrio cholerae	GCF_003348235	BD26	Water	Bangladesh	G10											
Vibrio cholerae	GCF_003348755	OYP8E03	Water	USA	G10											
Vibrio cholerae	GCF_003348985	OYP6F09	Water	USA	G10											
Vibrio cholerae	GCF_003349495	OYP1E12	Water	USA	G10											
Vibrio cholerae	GCF_003349525	OYP6D03	Water	USA	G10											
Vibrio cholerae	GCF_003349745	OYP3D09	Water	USA	G10											
Vibrio cholerae	GCF_003716465	3523-03	Human	Unknown	G10											
Vibrio cholerae	GCF_008369525	V919-19	Bird	Germany	G10											
Vibrio cholerae	GCF_013357685	L6G	Water	USA	G10											
Vibrio cholerae	GCF_013357765	SP7G	Water	USA	G10											
Vibrio cholerae	GCF_013357805	W7G	Water	USA	G10											
Vibrio cholerae	GCF_013357845	SA7G	Water	USA	G10											
Vibrio cholerae	GCF_017426915	DL4211	Water	USA	G10											
Vibrio cholerae	GCF_017426965	DL4215	Water	USA	G10											
Vibrio cholerae	GCF_021014515	VN-10143	Blue mussel	Germany	G10											
Vibrio cholerae	GCF_021014825	VN-10012	Oyster	Germany	G10											
Vibrio cholerae	GCF_024743545	RM-195-1	Mollusks	Canada	G10											
Vibrio cholerae	GCF_024746215	ISF-77-7	Shrimps	Indonesia	G10											
Vibrio cholerae	GCF_025258925	PNUSAV001140	Homo sapiens	USA	G10											
Vibrio cholerae	GCF_032820355	N17-2730	Homo sapiens	Switzerland	G10											
Vibrio cholerae	GCF_035782235	R18301	Environment	Brazil	G10											
Vibrio cholerae	GCF_902810005	4772STDY6941189	Unknown	Argentina	G10											
Vibrio cholerae	this study	IRLE0043_S52	Sediment	USA (Florida)	G10											
Vibrio cholerae	this study	IRLE0047_S54	Sediment	USA (Florida)	G10											
Vibrio cholerae	this study	IRLE0079_S67	Cyanobacteria	USA (Florida)	G10											
Vibrio cholerae	this study	IRLE0122_S74	Water	USA (Florida)	G10											
Vibrio cholerae	this study	IRLE0140_S76	Water	USA (Florida)	G10											
Vibrio cholerae	this study	IRLE0173_S78	Water	USA (Florida)	G10											
Vibrio cholerae	this study	IRLE0190_S84	Water	USA (Florida)	G10											
Vibrio cholerae	this study	IRLE0191_S85	Water	USA (Florida)	G10											
Vibrio cholerae	this study	IRLE0192_S86	Water	USA (Florida)	G10											
Vibrio cholerae	GCF_000152445	RC385	Plankton	USA	G11											
Vibrio cholerae	GCF_001857175	VC56	Oyster	USA	G11											
Vibrio cholerae	this study	IRLE0046_S53	Sediment	USA (Florida)	G11											
Vibrio cholerae	this study	IRLE0058_S58	Water	USA (Florida)	G11											
Vibrio cholerae	this study	IRLE0067_S60	Water	USA (Florida)	G11											
Vibrio cholerae	this study	IRLE0083_S71	Water	USA (Florida)	G11											
Vibrio cholerae	this study	IRLE0085_S72	Cyanobacteria	USA (Florida)	G11											
Vibrio cholerae	this study	IRLE0086_S73	Cyanobacteria	USA (Florida)	G11											
Vibrio cholerae	GCF_024105835	PGB-VCH018	Water	Algeria	G2						x					
Vibrio cholerae	GCF_015482825	C6706 (PCG)	Homo sapiens	Peru	G3	PCL	x	x	x	x	x	x	x	x		x
Vibrio cholerae	GCF_003574155	V060002	Human	Indonesia	G3	PCL	x	x	x	x	x	x	x	x	x	
Vibrio cholerae	GCF_000154005	623-39	Water	Bangladesh	G3	PCL					x					
Vibrio cholerae	GCF_008084375	N2767	Unknown	Unknown	G3	PCL					x					
Vibrio cholerae	GCF_008369605	RFB16	Water	USA	G3	PCL					x					
Vibrio cholerae	GCF_019273615	Colony98	Food	Thailand	G3	PCL					x					
Vibrio cholerae	GCF_024359565	MMRF 1421	Seawater	India	G3	PCL					x					
Vibrio cholerae	GCF_027158325	MMRF1896	Water	India	G3	PCL					x					
Vibrio cholerae	GCF_032027525	vc962760	Homo sapiens	China	G3	PCL					x					
Vibrio cholerae	GCF_033803285	975275	Homo sapiens	China	G3	PCL					x					
Vibrio cholerae	GCF_037154335	non-O1/non-O150	Homo sapiens	Spain	G3	PCL					x					
Vibrio cholerae	this study	IRLE0123_S75	Water	USA (Florida)	G3	PCL					x					
Vibrio cholerae	this study	IRLE0178_S79	Water	USA (Florida)	G3	PCL					x					
Vibrio cholerae	GCF_009938115	O1 AAS91	Unknown	Haiti	G3	PCL	x	x	x							
Vibrio cholerae	GCF_002284475	OYP2C05	Water	USA	G3	PCL										
Vibrio cholerae	GCF_003347715	BD60	Water	Bangladesh	G3	PCL										
Vibrio cholerae	GCF_003347995	BD43	Water	Bangladesh	G3	PCL										
Vibrio cholerae	GCF_003348065	BD32	Water	Bangladesh	G3	PCL										
Vibrio cholerae	GCF_008083975	N2780	Unknown	Unknown	G3	PCL										
Vibrio cholerae	GCF_016456845	Vc2633	Shrimp	China	G3	PCL										
Vibrio cholerae	GCF_016456875	Vc2573	Shrimp	China	G3	PCL										
Vibrio cholerae	GCF_016457125	Vc384	Shrimp	China	G3	PCL										
Vibrio cholerae	GCF_025505175	S35	Water	Ghana	G3	PCL										
Vibrio cholerae	GCF_026613265	NIHE_75	Environment	Vietnam	G3	PCL										
Vibrio cholerae	GCF_026613685	NIHE_54	Environment	Vietnam	G3	PCL										
Vibrio cholerae	GCF_032819495	N21-3623	Homo sapiens	Switzerland	G3	PCL										
Vibrio cholerae	GCF_032820295	N18-0491	Homo sapiens	Switzerland	G3	PCL										
Vibrio cholerae	GCF_037154295	non-O1/non-O154	Homo sapiens	Spain	G3	PCL										
Vibrio cholerae	GCF_037196065	vc-MS2-1	Shrimps	China	G3	PCL										
Vibrio cholerae	GCF_900185995	BC1071	Human	Germany	G3	PCL										
Vibrio cholerae	this study	IRLE0073_S63	Cyanobacteria	USA (Florida)	G3	PCL										
Vibrio cholerae	this study	IRLE0181_S80	Water	USA (Florida)	G3	PCL										
Vibrio cholerae	this study	IRLE0183_S82	Water	USA (Florida)	G3	PCL										
Vibrio cholerae	this study	IRLE0187_S83	Water	USA (Florida)	G3	PCL										
Vibrio cholerae	GCF_015168795	VC_hf7	Human	China	G3						x			x	x	
Vibrio cholerae	GCF_017814615	H20002	Human	China	G3						x			x	x	
Vibrio cholerae	GCF_000168935	MZO-3	Human	Bangladesh	G3									x	x	
Vibrio cholerae	GCF_008083865	N2786	Unknown	Unknown	G3						x				x	
Vibrio cholerae	GCF_008085625	N2723	Unknown	Unknown	G3						x				x	
Vibrio cholerae	GCF_000174295	TMA 21	Seawater	Brazil	G3						x			x		
Vibrio cholerae	GCF_021166935	173	Homo sapiens	Russia	G3									x		
Vibrio cholerae	GCF_036873435	16-VB00110	Mussels	Germany (Baltic Sea)	G3									x		
Vibrio cholerae	GCF_026613135	NIHE_82	Environment	Vietnam	G3						x		x			
Vibrio cholerae	GCF_013111355	PivertUAT4Aug	Environment	Haiti	G3			x	x	x	x	x				
Vibrio cholerae	GCF_036814005	17-VB00080	Shrimps	Germany (Baltic Sea)	G3						x	x				
Vibrio cholerae	GCF_008084795	N2722	Unknown	Unknown	G3							x				
Vibrio cholerae	GCF_016456805	Vc2634	Shrimp	China	G3							x				
Vibrio cholerae	GCF_021431945	GXFL1-4	/lacrobrachium rosenberg	China	G3							x				
Vibrio cholerae	GCF_036813985	16-VB00247	Shrimps	Germany (Baltic Sea)	G3							x				
Vibrio cholerae	GCF_026613655	NIHE_58	Environment	Vietnam	G3				x		x					

Vibrio cholerae	GCF_000174115	12129(1)	Water	Australia	G3	x			x			
Vibrio cholerae	GCF_001729195	VC22	Oyster	USA	G3	x			x			
Vibrio cholerae	GCF_003057035	3178	Water	Russia	G3	x			x			
Vibrio cholerae	GCF_026013235	E1	Water	China	G3	x			x			
Vibrio cholerae	GCF_000153785	AM-19226	Human	Bangladesh	G3				x			
Vibrio cholerae	GCF_000168895	1587	Human	Peru	G3				x			
Vibrio cholerae	GCF_000348345	O1 str. EM-1676A	Water	Bangladesh	G3				x			
Vibrio cholerae	GCF_000348425	O1 str. NHCC-008D	Human	Bangladesh	G3				x			
Vibrio cholerae	GCF_001402605	YB4B03	Oyster	USA	G3				x			
Vibrio cholerae	GCF_002196165	5	Human	Ukraine	G3				x			
Vibrio cholerae	GCF_002284355	OYP3F10	Water	USA	G3				x			
Vibrio cholerae	GCF_003349575	OYP5H09	Water	USA	G3				x			
Vibrio cholerae	GCF_003594515	195V0316	Water	Bangladesh	G3				x			
Vibrio cholerae	GCF_008083425	N2815	Unknown	Unknown	G3				x			
Vibrio cholerae	GCF_008083615	N2799	Unknown	Unknown	G3				x			
Vibrio cholerae	GCF_008083635	N2798	Unknown	Unknown	G3				x			
Vibrio cholerae	GCF_008083785	N2788	Unknown	Unknown	G3				x			
Vibrio cholerae	GCF_008083915	N2783	Unknown	Unknown	G3				x			
Vibrio cholerae	GCF_008084565	N2754	Unknown	Unknown	G3				x			
Vibrio cholerae	GCF_008084645	N2752	Unknown	Unknown	G3				x			
Vibrio cholerae	GCF_013414365	O139 NALMLE47	Human	Bangladesh	G3				x			
Vibrio cholerae	GCF_014654545	H10	Human	Qatar	G3				x			
Vibrio cholerae	GCF_019273655	Colony605	Food	Thailand	G3				x			
Vibrio cholerae	GCF_021228575	VCH20210731	Homo sapiens	China	G3				x			
Vibrio cholerae	GCF_022758165	VCHL017	Homo sapiens	China	G3				x			
Vibrio cholerae	GCF_023488285	153	Homo sapiens	USA	G3				x			
Vibrio cholerae	GCF_024745295	ISF-237-6	Shrimps	India	G3				x			
Vibrio cholerae	GCF_024745515	ISF-208-8	Shrimps	Thailand	G3				x			
Vibrio cholerae	GCF_026613215	NIHE_78	Environment	Vietnam	G3				x			
Vibrio cholerae	GCF_026613295	NIHE_74	Environment	Vietnam	G3				x			
Vibrio cholerae	GCF_026613455	NIHE_67	Environment	Vietnam	G3				x			
Vibrio cholerae	GCF_026613975	NIHE_39	Environment	Vietnam	G3				x			
Vibrio cholerae	GCF_027921725	22-19	Water	Russia	G3				x			
Vibrio cholerae	GCF_032819435	N22-0171	Homo sapiens	Switzerland	G3				x			
Vibrio cholerae	GCF_032819455	N21-2239	Homo sapiens	Switzerland	G3				x			
Vibrio cholerae	GCF_032819855	N18-2287	Homo sapiens	Switzerland	G3				x			
Vibrio cholerae	GCF_036814085	17-VB00089	Black tiger shrimps	Germany (Baltic Sea)	G3				x			
Vibrio cholerae	GCF_036888945	B20286	Estuarine water	Ireland	G3				x			
Vibrio cholerae	GCF_037150575	non-O1/non-O164	Homo sapiens	Spain	G3				x			
Vibrio cholerae	GCF_037462815	18-VB00278	Sediment	Germany (Baltic Sea)	G3				x			
Vibrio cholerae	GCF_019458465	ICDC-VC702	Frog	Unknown	G3			x				
Vibrio cholerae	GCF_000153985	MZO-2	Human	Bangladesh	G3							
Vibrio cholerae	GCF_000788495	2012Env-2	Water	Haiti	G3							
Vibrio cholerae	GCF_000969235	1154-74	Water	India	G3							
Vibrio cholerae	GCF_002097735	CISM_300055	Human	Mozambique	G3							
Vibrio cholerae	GCF_003096135	9507	Human	Russia	G3							
Vibrio cholerae	GCF_008083925	N2782	Unknown	Unknown	G3							
Vibrio cholerae	GCF_008084425	N2766	Unknown	Unknown	G3							
Vibrio cholerae	GCF_008084455	N2762	Unknown	Unknown	G3							
Vibrio cholerae	GCF_008084655	N2742	Unknown	Unknown	G3							
Vibrio cholerae	GCF_008084705	N2735	Unknown	Unknown	G3							
Vibrio cholerae	GCF_008085535	N2744	Unknown	Unknown	G3							
Vibrio cholerae	GCF_013111175	EDC_701	Environment	Bangladesh	G3							
Vibrio cholerae	GCF_013111215	EDC_694	Environment	Bangladesh	G3							
Vibrio cholerae	GCF_013111295	EDC_689	Environment	Bangladesh	G3							
Vibrio cholerae	GCF_013357605	W10G	Water	USA	G3							
Vibrio cholerae	GCF_016456985	Vc730	Shrimp	China	G3							
Vibrio cholerae	GCF_019273635	Colony268	Food	Thailand	G3							
Vibrio cholerae	GCF_025505255	S1	Water	Ghana	G3							
Vibrio cholerae	GCF_025563555	E4	Environment	Vietnam	G3							
Vibrio cholerae	GCF_026613195	NIHE_80	Environment	Vietnam	G3							
Vibrio cholerae	GCF_026613665	NIHE_55	Environment	Vietnam	G3							
Vibrio cholerae	GCF_026613775	NIHE_56	Environment	Vietnam	G3							
Vibrio cholerae	GCF_026613875	NIHE_47	Environment	Vietnam	G3							
Vibrio cholerae	GCF_027158335	MMRF1910	Water	India	G3							
Vibrio cholerae	GCF_027921685	1-19	Water	Russia	G3							
Vibrio cholerae	GCF_029906585	2352495169	Homo sapiens	China	G3							
Vibrio cholerae	GCF_032819415	N21-2192	Homo sapiens	Switzerland	G3							
Vibrio cholerae	GCF_032819615	N19-2998	Homo sapiens	Switzerland	G3							
Vibrio cholerae	GCF_032819975	N18-1815	Homo sapiens	Switzerland	G3							
Vibrio cholerae	GCF_032820115	N18-1566	Homo sapiens	Switzerland	G3							
Vibrio cholerae	GCF_032841475	973360	Homo sapiens	China	G3							
Vibrio cholerae	GCF_035782295	R18297	Environment	Brazil	G3							
Vibrio cholerae	GCF_035782415	R18281	Environment	Brazil	G3							
Vibrio cholerae	GCF_036441435	14-VB00093	King prawns	Germany (Baltic Sea)	G3							
Vibrio cholerae	GCF_036441475	16-VB00025	Mussels	Germany (Baltic Sea)	G3							
Vibrio cholerae	GCF_036441615	16-VB00077	Mussels	Germany (Baltic Sea)	G3							
Vibrio cholerae	GCF_036814045	17-VB00067	Shrimps	Germany (Baltic Sea)	G3							
Vibrio cholerae	GCF_036814175	17-VB00147	White tiger shrimps	Germany	G3							
Vibrio cholerae	GCF_036814215	17-VB00226	Mussels	Germany (Baltic Sea)	G3							
Vibrio cholerae	GCF_037150415	non-O1/non-O166	Homo sapiens	Spain	G3							
Vibrio cholerae	GCF_037150455	non-O1/non-O160	Homo sapiens	Spain	G3							
Vibrio cholerae	GCF_037150515	non-O1/non-O165	Homo sapiens	Spain	G3							
Vibrio cholerae	GCF_037154445	non-O1/non-O149	Homo sapiens	Spain	G3							
Vibrio cholerae	GCF_037154575	non-O1/non-O151	Homo sapiens	Spain	G3							
Vibrio cholerae	GCF_037196005	vc-CLC6-1	Chicken	China	G3							
Vibrio cholerae	GCF_037196045	vc-TS4-1	Shrimps	China	G3							
Vibrio cholerae	GCF_037462755	18-VB00347	water	Germany (Baltic Sea)	G3							
Vibrio cholerae	GCF_902809995	4772STDY6940818	Unknown	Argentina	G3							
Vibrio cholerae	this study	IRLE0076_S65	Cyanobacteria	USA (Florida)	G3							
Vibrio cholerae	this study	IRLE0169_S77	Water	USA (Florida)	G3							
Vibrio cholerae	this study	IRLE0182_S81	Water	USA (Florida)	G3							
Vibrio cholerae	GCF_001254095	A325	Unknown	Argentina	G4	x			x			x
Vibrio cholerae	GCF_014654695	H1(2020)	Human	Haiti	G4				x			x
Vibrio cholerae	GCF_030464185	20-17	Water	Russia	G4				x			x
Vibrio cholerae	GCF_001402585	YB4H02	Oyster	USA	G4				x			
Vibrio cholerae	GCF_009763045	3541-04	Unknown	Unknown	G4		x	x	x	x		
Vibrio cholerae	GCF_000969265	10432-62	Human	Philippines	G4				x	x		
Vibrio cholerae	GCF_009762895	3528-08	Human	USA	G4		x	x	x			
Vibrio cholerae	GCF_001857165	VC48	Oyster	USA	G4			x	x			
Vibrio cholerae	GCF_012275105	2010V-1116	Unknown	Haiti	G4			x	x			
Vibrio cholerae	GCF_014525175	2009V-1135	Unknown	USA	G4			x	x			
Vibrio cholerae	this study	IRLE0081_S69	Cyanobacteria	USA (Florida)	G4			x	x			
Vibrio cholerae	GCF_007624435	EL2313	Unknown	Unknown	G4	x			x			
Vibrio cholerae	GCF_008269325	3017	Water	Russia	G4	x			x			
Vibrio cholerae	GCF_008269345	136	Water	Russia	G4	x			x			
Vibrio cholerae	GCF_008269455	M1467	Water	Russia	G4	x			x			
Vibrio cholerae	GCF_008370445	29	Water	Russia	G4	x			x			
Vibrio cholerae	GCF_008370875	M1526	Water	Russia	G4	x			x			
Vibrio cholerae	GCF_001718105	L11	Mussel	Sweden	G4				x			
Vibrio cholerae	GCF_002890525	11116	Human	Sweden	G4				x			
Vibrio cholerae	GCF_003349645	OYP4H04	Oyster	USA	G4				x			
Vibrio cholerae	GCF_003716515	2015V-1118	Human	USA	G4				x			
Vibrio cholerae	GCF_006803155	A110509W1	Water	Austria	G4				x			
Vibrio cholerae	GCF_008084055	N2774	Unknown	Unknown	G4				x			
Vibrio cholerae	GCF_008084605	N2751	Unknown	Unknown	G4				x			
Vibrio cholerae	GCF_013357625	SL4G	Water	USA	G4				x			
Vibrio cholerae	GCF_013357885	SA3G	Water	USA	G4				x			
Vibrio cholerae	GCF_014634285	PS-7702	Fish	Japan	G4				x			
Vibrio cholerae	GCF_026613575	NIHE_61	Environment	Vietnam	G4				x			
Vibrio cholerae	GCF_026613635	NIHE_57	Environment	Vietnam	G4				x			
Vibrio cholerae	GCF_037154515	non-O1/non-O147	Homo sapiens	Spain	G4				x			
Vibrio cholerae	GCF_037154535	non-O1/non-O143	Homo sapiens	Spain	G4				x			
Vibrio cholerae	GCF_037462795	18-VB00287	water	Germany (Baltic Sea)	G4				x			
Vibrio cholerae	GCF_026613885	NIHE_45	Environment	Vietnam	G4	x						
Vibrio cholerae	GCF_030464195	7-17	Silt	Russia	G4	x						
Vibrio cholerae	GCF_016456815	Vc2632	Shrimp	China	G4							
Vibrio cholerae	GCF_016457005	Vc517	Shrimp	China	G4							
Vibrio cholerae	GCF_017426985	DL4213	Water	USA	G4							
Vibrio cholerae	GCF_035782315	R18350	Environment	Brazil	G4							
Vibrio cholerae	this study	IRLE0082_S70	Cyanobacteria	USA (Florida)	G4							
Vibrio cholerae	GCF_002284495	OYP1G01	Water	USA	G5							
Vibrio cholerae	GCF_003349145	OYP6D07	Water	USA	G5							
Vibrio cholerae	GCF_005636015	VcCHNf2	Seawater	China	G6						x	
Vibrio cholerae	GCF_005636075	VcCHNf4	Seawater	China	G6						x	
Vibrio cholerae	GCF_001718095	L15	Mussel	Sweden	G6				x			
Vibrio cholerae	GCF_003347815	BD56	Water	Bangladesh	G6							
Vibrio cholerae	GCF_003348035	BD37	Water	Bangladesh	G6							
Vibrio cholerae	GCF_003449115	VN-2825	Water	Germany	G6							
Vibrio cholerae	GCF_006802795	A12JL4W4	Water	Austria	G6							
Vibrio cholerae	GCF_016456945	Vc601	Shrimp	China	G6							
Vibrio cholerae	GCF_017426745	DL2111	Water	USA	G6							
Vibrio cholerae	GCF_021014705	VN-0462	seawater	Germany	G6							
Vibrio cholerae	GCF_021014725	VN-00470	seawater	Germany	G6							
Vibrio cholerae	GCF_021014745	VN-00471	seawater	Germany	G6							
Vibrio cholerae	GCF_021014875	VN-10133	Oyster	Germany	G6							
Vibrio cholerae	GCF_021166975	41	Homo sapiens	Germany	G6							
Vibrio cholerae	GCF_036814105	17-VB00115	Black tiger shrimps	Germany (Baltic Sea)	G6							
Vibrio cholerae	GCF_036873405	16-VB00130	Black tiger shrimps	Germany (Baltic Sea)	G6							
Vibrio cholerae	GCF_037154215	non-O1/non-O141	Homo sapiens	Spain	G6							
Vibrio cholerae	GCF_037154495	non-O1/non-O148	Homo sapiens	Spain	G6							

Vibrio cholerae	GCF_037462435	18-VB00676	water	Germany (Baltic Sea)	G6			
Vibrio cholerae	GCF_037462445	18-VB00656	water	Germany (Baltic Sea)	G6			
Vibrio cholerae	GCF_037462495	18-VB00596	water	Germany (Baltic Sea)	G6			
Vibrio cholerae	GCF_037462505	18-VB00598	water	Germany (Baltic Sea)	G6			
Vibrio cholerae	GCF_037462605	18-VB00587	water	Germany (Baltic Sea)	G6			
Vibrio cholerae	GCF_037462765	18-VB00405	water	Germany (Baltic Sea)	G6			
Vibrio cholerae	GCF_037462855	18-VB00273	Sediment	Germany (Baltic Sea)	G6			
Vibrio cholerae	GCF_037462955	18-VB00160	water	Germany (Baltic Sea)	G6			
Vibrio cholerae	GCF_001471585	FDAARGOS_103	Fish	Germany	G7			x
Vibrio cholerae	GCF_008083415	N2814	Unknown	Unknown	G7		x	
Vibrio cholerae	GCF_008083715	N2793	Unknown	Unknown	G7		x	
Vibrio cholerae	GCF_037154475	non-O1/non-O140	Homo sapiens	Spain	G7		x	
Vibrio cholerae	GCF_013111105	EDC_715	Environment	Bangladesh	G7	x		
Vibrio cholerae	GCF_003347775	BD55	Water	Bangladesh	G7			
Vibrio cholerae	GCF_003348095	BD31	Water	Bangladesh	G7			
Vibrio cholerae	GCF_003348305	BD17	Water	Bangladesh	G7			
Vibrio cholerae	GCF_003348415	BD11	Water	Bangladesh	G7			
Vibrio cholerae	GCF_003348675	BD07	Water	Bangladesh	G7			
Vibrio cholerae	GCF_003348735	BD01	Water	Bangladesh	G7			
Vibrio cholerae	GCF_003716505	2015V-1126	Human	USA	G7			
Vibrio cholerae	GCF_005636045	VcCHN8	Seawater	China	G7			
Vibrio cholerae	GCF_006802075	920008-15	Human	Austria	G7			
Vibrio cholerae	GCF_006802145	P12-CHT68-05	Human	Austria	G7			
Vibrio cholerae	GCF_006802165	P19-CHT78-07	Human	Austria	G7			
Vibrio cholerae	GCF_006802245	P2-CHT15-00	Human	Austria	G7			
Vibrio cholerae	GCF_006802295	A12JL36W91	Water	Austria	G7			
Vibrio cholerae	GCF_006802355	A12JL36W74	Water	Austria	G7			
Vibrio cholerae	GCF_006802365	A12JL36W67	Water	Austria	G7			
Vibrio cholerae	GCF_006802375	A12JL36W49	Water	Austria	G7			
Vibrio cholerae	GCF_006802445	A12JL36W30	Water	Austria	G7			
Vibrio cholerae	GCF_006802495	A12JL36W17	Water	Austria	G7			
Vibrio cholerae	GCF_006802555	A12JL5W86	Water	Austria	G7			
Vibrio cholerae	GCF_006802605	A12JL5W14	Water	Austria	G7			
Vibrio cholerae	GCF_006802635	A12JL4W93	Water	Austria	G7			
Vibrio cholerae	GCF_006802685	A12JL4W72	Water	Austria	G7			
Vibrio cholerae	GCF_006802705	A12JL4W81	Water	Austria	G7			
Vibrio cholerae	GCF_006802715	A12JL4W21	Water	Austria	G7			
Vibrio cholerae	GCF_006802785	A121001W2	Water	Austria	G7			
Vibrio cholerae	GCF_006802815	A121001W1	Water	Austria	G7			
Vibrio cholerae	GCF_006802865	A120716Z3	Zooplankton	Austria	G7			
Vibrio cholerae	GCF_006802935	A120502W2	Water	Austria	G7			
Vibrio cholerae	GCF_006803005	A110926W4	Water	Austria	G7			
Vibrio cholerae	GCF_006803105	A110704W4	Water	Austria	G7			
Vibrio cholerae	GCF_008083365	N2817	Unknown	Unknown	G7			
Vibrio cholerae	GCF_008083875	N2785	Unknown	Unknown	G7			
Vibrio cholerae	GCF_008085635	N2725	Unknown	Unknown	G7			
Vibrio cholerae	GCF_013111275	EDC_688	Environment	Bangladesh	G7			
Vibrio cholerae	GCF_014281135	C16-2-29	Animal	China	G7			
Vibrio cholerae	GCF_017426785	DL2114	Water	USA	G7			
Vibrio cholerae	GCF_021166915	735	Homo sapiens	Russia	G7			
Vibrio cholerae	GCF_021166955	1175	Homo sapiens	Russia	G7			
Vibrio cholerae	GCF_021167035	20284	Homo sapiens	Russia	G7			
Vibrio cholerae	GCF_026613475	NIHE_64	Environment	Vietnam	G7			
Vibrio cholerae	GCF_026613815	NIHE_49	Environment	Vietnam	G7			
Vibrio cholerae	GCF_030908845	897_2023_HUN_NNGYK	Water	Hungary	G7			
Vibrio cholerae	GCF_034317045	NB-183	Environmental	Canada	G7			
Vibrio cholerae	GCF_036873385	17-VB00162	Mussels	Germany (Baltic Sea)	G7			
Vibrio cholerae	GCF_037101305	VN152	Mastacembelus	Vietnam	G7			
Vibrio cholerae	GCF_037150475	non-O1/non-O163	Homo sapiens	Spain	G7			
Vibrio cholerae	GCF_037154255	non-O1/non-O155	Homo sapiens	Spain	G7			
Vibrio cholerae	GCF_037154375	non-O1/non-O156	Homo sapiens	Spain	G7			
Vibrio cholerae	GCF_037462705	18-VB00559	water	Germany (Baltic Sea)	G7			
Vibrio cholerae	GCF_038448525	VB-27	Bird	China	G7			
Vibrio cholerae	GCF_039602145	LK-18	Procambarus clarkii	China	G7			
Vibrio cholerae	this study	IRLE0050_S56	Sediment	USA (Florida)	G8			
Vibrio cholerae	this study	IRLE0074_S64	Cyanobacteria	USA (Florida)	G8			
Vibrio cholerae	this study	IRLE0049_S55	Sediment	USA (Florida)	G9			x
Vibrio cholerae	this study	IRLE0062_S59	Water	USA (Florida)	G9			x
Vibrio cholerae	this study	IRLE0077_S66	Cyanobacteria	USA (Florida)	G9			x
Vibrio cholerae	GCF_000221465	BJG-01	Unknown	Unknown	G9			
Vibrio cholerae	GCF_003348365	BD18	Water	Bangladesh	G9			
Vibrio cholerae	GCF_003349055	OYP6E05	Water	USA	G9			
Vibrio cholerae	GCF_024745065	ISF-243-9	Shrimps	India	G9			
Vibrio cholerae	GCF_036888835	B20237A	Seawater	Ireland	G9			
Vibrio cholerae	GCF_037462595	18-VB00588	water	Germany (Baltic Sea)	G9			
Vibrio cholerae	this study	IRLE0042_S51	Sediment	USA (Florida)	G9			
Vibrio cholerae	this study	IRLE0052_S57	Water	USA (Florida)	G9			
Vibrio cholerae	this study	IRLE0071_S61	Water	USA (Florida)	G9			
Vibrio cholerae	this study	IRLE0080_S68	Cyanobacteria	USA (Florida)	G9			

Table S3. Unique genes found in the PCG and acquired from PCL and G3.

Hierarchical levels	*Vibrio cholerae* C6706	Chromosome	*Vibrio cholerae* O1 biovar eltor str. N16961	Reference	Annotation	Genomic Island
PCG	C6706-C1-cds0136	I	VC_0455	-	HemN COG0635 416 Coproporphyrinogen III oxidase and related Fe-S oxidoreductases	
PCG	C6706-C1-cds0168	I	-	-	OG0449 597 Glucosamine 6-phosphate synthetase, contains amidotransferase and phosphosugar isomerase domains	
PCG	C6706-C1-cds0171	I	-	-	COG3176 COG3176 292 Putative hemolysin	
PCG	C6706-C1-cds0181	I	-	-	Tra5 COG2801 232 Transposase and inactivated derivatives	
PCG	C6706-C1-cds0182	I	-	-	COG2963 COG2963 116 Transposase and inactivated derivatives	
PCG	C6706-C1-cds0183	I	-	-	-	
PCG	C6706-C1-cds0247	I	VC_0568	-	COG1485 COG1485 367 Predicted ATPase	
PCG	C6706-C1-cds0277	I	-	-	-	
PCG	C6706-C1-cds0285	I	-	-	PotA COG3842 352 ABC-type spermidine/putrescine transport systems, ATPase components	
PCG	C6706-C1-cds0329	I	-	-	-	
PCG	C6706-C1-cds0474	I	VC_0811		-	
PCG	C6706-C1-cds0480	I	-	-	-	
PCG	C6706-C1-cds0508	I	-	-	-	
PCG	C6706-C1-cds0588	I	VC_0934	-	COG1086 COG1086 588 Predicted nucleoside-diphosphate sugar epimerases	
PCG	C6706-C1-cds0688	I	-	-	FadL COG2067 440 Long-chain fatty acid transport protein	
PCG	C6706-C1-cds0852	I	-	-	UvrC COG0322 581 Nuclease subunit of the excinuclease complex	
PCG	C6706-C1-cds0923	I	-	-	COG2206 COG2206 344 c-di-GMP phosphodiesterase class II (HD-GYP domain)	
PCG	C6706-C1-cds1071	I	VC_1453	AE003852	-	CTX-Phi-cds2|CTX-Phi
PCG	C6706-C1-cds1095	I	-	-	-	
PCG	C6706-C1-cds1097	I	-	-	COG2963 COG2963 116 Transposase and inactivated derivatives	
PCG	C6706-C1-cds1098	I	VC_A0791	AE003853	Tra5 COG2801 232 Transposase and inactivated derivatives	
PCG	C6706-C1-cds1123	I	-	-	-	
PCG	C6706-C1-cds1173	I	-	-	PepN COG0308 859 Aminopeptidase N	
PCG	C6706-C1-cds1206	I	VC_1528	AE003852	HisJ COG0834 275 ABC-type amino acid transport/signal transduction systems, periplasmic component/domain	
PCG	C6706-C1-cds1207	I	VC_1529	AE003852	-	
PCG	C6706-C1-cds1218	I	VC_1541	AE003852	-	
PCG	C6706-C1-cds1281	I	VC_1607	-	conserved_hypothetical_protein	
PCG	C6706-C1-cds1361	I	VC_1689	AE003852	-	
PCG	C6706-C1-cds1362	I	VC_1690	AE003852	GalA COG3345 687 Alpha-galactosidase	
PCG	C6706-C1-cds1422	I	-	-	XerC COG0582 309 Integrase	VPI-2-cds2|VPI-2
PCG	C6706-C1-cds1430	I	VC_1768	AE003852	HsdS COG0732 391 Restriction endonuclease S subunits	VPI-2-cds11|VPI-2
PCG	C6706-C1-cds1448	I	VC_1787	AE003852	-	VPI-2-cds29|VPI-2
PCG	C6706-C1-cds1449	I	VC_A0370	AE003853	-	VPI-2-cds30|VPI-2
PCG	C6706-C1-cds1450	I	VC_A0791	AE003853	Tra5 COG2801 232 Transposase and inactivated derivatives	VPI-2-cds31|VPI-2
PCG	C6706-C1-cds1452	I	VC_1791	AE003852	COG3941 COG3941 633 Mu-like prophage protein	VPI-2-cds33|VPI-2
PCG	C6706-C1-cds1469	I	-	-	-	VPI-2-cds50|VPI-2
PCG	C6706-C1-cds1470	I	-	-	-	
PCG	C6706-C1-cds1583	I	VC_1926	-	C COG2204 464 Response regulator containing CheY-like receiver, AAA-type ATPase, and DNA-binding domains	
PCG	C6706-C1-cds1616	I	-	-	COG3492 COG3492 104 Uncharacterized protein conserved in bacteria	
PCG	C6706-C1-cds1768	I	VC_2121	-	FliR COG1684 258 Flagellar biosynthesis pathway, component FliR	
PCG	C6706-C1-cds1852	I	VC_2212	AE003852	-	
PCG	C6706-C1-cds1856	I	VC_2215	-	ZntA COG2217 713 Cation transport ATPase	
PCG	C6706-C1-cds2013	I	-	-	-	
PCG	C6706-C1-cds2105	I	VC_2484	-	FAA1 COG1022 613 Long-chain acyl-CoA synthetases (AMP-forming)	
PCG	C6706-C1-cds2304	I	-	-	-	
PCG	C6706-C1-cds2305	I	-	-	-	
PCG	C6706-C1-cds2331	I	VC_2726	-	PulK COG3156 323 Type II secretory pathway, component PulK	
PCG	C6706-C1-cds2543	I	VC_0183	-	-	
PCG	C6706-C1-cds2546	I	-	-	XerC COG0582 309 Integrase	
PCG	C6706-C1-cds2547	I	VC_A0791	AE003853	Tra5 COG2801 232 Transposase and inactivated derivatives	
PCG	C6706-C1-cds2549	I	-	-	-	
PCG	C6706-C1-cds2589	I	VC_0229	-	-	
PCG	C6706-C1-cds2614	I	-	-	-	Capsule-cds19|Capsule
PCG	C6706-C1-cds2615	I	-	-	-	Capsule-cds20|Capsule
PCG	C6706-C1-cds2616	I	-	-	-	
PCG	C6706-C1-cds2617	I	-	-	-	
PCG	C6706-C1-cds2640	I	-	-	CirA COG1629 768 Outer membrane receptor proteins, mostly Fe transport	
PCG	C6706-C1-cds2641	I	-	-	CirA COG1629 768 Outer membrane receptor proteins, mostly Fe transport	
PCG	C6706-C2-cds117	II	-	-	-	
PCG	C6706-C2-cds197	II	VC_A0163	AE003853	COG3530 COG3530 71 Uncharacterized protein conserved in bacteria	
PCG	C6706-C2-cds230	II	VC_A0199 70	-	-	
PCG	C6706-C2-cds239	II	-	-	-	
PCG	C6706-C2-cds256	II	VC_A0228 70	-	CeuB COG4606 321 ABC-type enterochelin transport system, permease component	
PCG	C6706-C2-cds264	II	VC_A0236	AE003853	-	
PCG	C6706-C2-cds280	II	-	-	-	
PCG	C6706-C2-cds327	II	VC_A0385	AE003853	ParE COG3668 98 Plasmid stabilization system protein	Superintegron
PCG	C6706-C2-cds340	II	-	-	-	Superintegron
PCG	C6706-C2-cds342	II	-	-	-	Superintegron
PCG	C6706-C2-cds347	II	VC_A0338	AE003853	COG3324 COG3324 127 Predicted enzyme related to lactoylglutathione lyase	Superintegron
PCG	C6706-C2-cds354	II	VC_A0506	AE003853	GloA COG0346 138 Lactoylglutathione lyase and related lyases	Superintegron
PCG	C6706-C2-cds357	II	VC_A0350	AE003853	Blc COG3040 174 Bacterial lipocalin	Superintegron
PCG	C6706-C2-cds359	II	VC_A0354	AE003853	GrpB COG2320 185 Uncharacterized conserved protein	Superintegron
PCG	C6706-C2-cds368	II	VC_A0363	AE003853	-	Superintegron
PCG	C6706-C2-cds370	II	VC_A0293	AE003853	-	Superintegron
PCG	C6706-C2-cds374	II	-	-	-	Superintegron
PCG	C6706-C2-cds375	II	VC_A0370	AE003853	-	Superintegron
PCG	C6706-C2-cds376	II	VC_A0791	AE003853	Tra5 COG2801 232 Transposase and inactivated derivatives	Superintegron
PCG	C6706-C2-cds378	II	VC_A0374	AE003853	-	Superintegron
PCG	C6706-C2-cds393	II	-	-	-	Superintegron
PCG	C6706-C2-cds394	II	VC_A0398	AE003853	-	Superintegron
PCG	C6706-C2-cds400	II	VC_A0406	AE003853	Blc COG3040 174 Bacterial lipocalin	Superintegron
PCG	C6706-C2-cds405	II	VC_A0414	AE003853	-	Superintegron
PCG	C6706-C2-cds413	II	VC_A0423	AE003853	ParE COG3668 98 Plasmid stabilization system protein	Superintegron
PCG	C6706-C2-cds415	II	VC_A0426	AE003853	COG3313 COG3313 74 Predicted Fe-S protein	Superintegron
PCG	C6706-C2-cds416	II	VC_A0428	AE003853	-	Superintegron
PCG	C6706-C2-cds424	II	VC_A0442	AE003853	RimL COG1670 187 Acetyltransferases, including N-acetylases of ribosomal proteins	Superintegron
PCG	C6706-C2-cds437	II	-	-	Sbp COG1613 348 ABC-type sulfate transport system, periplasmic component	Superintegron
PCG	C6706-C2-cds448	II	VC_A0426	AE003853	COG3313 COG3313 74 Predicted Fe-S protein	Superintegron
PCG	C6706-C2-cds452	II	VC_A0471	AE003853	-	Superintegron
PCG	C6706-C2-cds454	II	VC_A0473	AE003853	-	Superintegron
PCG	C6706-C2-cds464	II	VC_A0484	AE003853	-	Superintegron
PCG	C6706-C2-cds466	II	-	-	COG4453 COG4453 95 Uncharacterized protein conserved in bacteria	Superintegron
PCG	C6706-C2-cds471	II	-	-	-	Superintegron
PCG	C6706-C2-cds472	II	-	-	-	Superintegron
PCG	C6706-C2-cds474	II	VC_A0494	AE003853	-	Superintegron
PCG	C6706-C2-cds482	II	VC_A0505	AE003853	RimL COG1670 187 Acetyltransferases, including N-acetylases of ribosomal proteins	Superintegron
PCG	C6706-C2-cds486	II	VC_A0791	AE003853	Tra5 COG2801 232 Transposase and inactivated derivatives	
PCG	C6706-C2-cds499	II	-	-	-	
PCG	C6706-C2-cds502	II	-	-	EriC COG0038 443 Chloride channel protein EriC	
PCG	C6706-C2-cds549	II	-	-	BtuB COG4206 608 Outer membrane cobalamin receptor protein	
PCG	C6706-C2-cds611	II	-	-	-	
PCG	C6706-C2-cds677	II	VC_A0716	-	COG3658 COG3658 192 Cytochrome b	
PCG	C6706-C2-cds748	II	VC_A0791	AE003853	Tra5 COG2801 232 Transposase and inactivated derivatives	
PCG	C6706-C2-cds751	II	VC_A0794	AE003853	-	
PCG	C6706-C2-cds831	II	-	-	ThiJ COG0693 188 Putative intracellular protease/amidase	
PCG	C6706-C2-cds860	II	VC_A0915	-	COG4559 COG4559 259 ABC-type hemin transport system, ATPase component	
PCG	C6706-C2-cds916	II	VC_A0979	AE003853	Tar COG0840 408 Methyl-accepting chemotaxis protein	
PCG	C6706-C2-cds917	II	VC_A0980	AE003853	-	
PCG	C6706-C2-cds975	II	VC_A1044	AE003853	-	
PCG, PCL	C6706-C1-cds0001	I	-	-	conserved hypothetical protein	
PCG, PCL	C6706-C1-cds0121	I	VC_0439	AE003852	COG3610 COG3610 156 Uncharacterized conserved protein	
PCG, PCL	C6706-C1-cds0122	I	VC_0440	AE003852	FolA COG0262 167 Dihydrofolate reductase	
PCG, PCL	C6706-C1-cds0283	I	VC_0608	AE003852	AfuA COG1840 299 ABC-type Fe3+ transport system, periplasmic component	
PCG, PCL	C6706-C1-cds1073	I	VC_1464	AE003852	HipB COG1396 120 Predicted transcriptional regulators	CTX-Phi-cds14|CTX-Phi
PCG, PCL	C6706-C1-cds1090	I	VC_1471	AE003852	-	
PCG, PCL	C6706-C1-cds1451	I	VC_1790	AE003852	COG2963 COG2963 116 Transposase and inactivated derivatives	VPI-2-cds32|VPI-2
PCG, PCL	C6706-C1-cds2275	I	VC_2669	AE003852	HpaF COG3232 127 5-carboxymethyl-2-hydroxymuconate isomerase	
PCG, PCL	C6706-C2-cds0058	II	VC_A0005	AE003853	-	
PCG, PCL	C6706-C2-cds262	II	VC_A0233	AE003853	COG3553 COG3553 96 Uncharacterized protein conserved in bacteria	
PCG, PCL	C6706-C2-cds328	II	VC_A0386	AE003853	-	Superintegron
PCG, PCL	C6706-C2-cds356	II	VC_A0504	AE003853	-	Superintegron
PCG, PCL	C6706-C2-cds360	II	VC_A0355	AE003853	RhtB COG1280 208 Putative threonine efflux protein	Superintegron
PCG, PCL	C6706-C2-cds366	II	VC_A0361	AE003853	-	Superintegron
PCG, PCL	C6706-C2-cds396	II	VC_A0479	AE003853	-	Superintegron
PCG, PCL	C6706-C2-cds408	II	-	-	-	Superintegron
PCG, PCL	C6706-C2-cds443	II	-	-	-	Superintegron
PCG, PCL, G3	C6706-C1-cds0031	I	VC_0345	AE003852	MutL COG0323 638 DNA mismatch repair enzyme (predicted ATPase)	
PCG, PCL, G3	C6706-C1-cds0032	I	VC_0346	AE003852	MiaA COG0324 308 tRNA delta(2)-isopentenylpyrophosphate transferase	
PCG, PCL, G3	C6706-C1-cds0197	I	VC_A0791	AE003852	Tra5 COG2801 232 Transposase and inactivated derivatives	
PCG, PCL, G3	C6706-C1-cds0420	I	VC_0754	AE003852	COG2975 COG2975 64 Uncharacterized protein conserved in bacteria	
PCG, PCL, G3	C6706-C1-cds0421	I	-	AE003852	-	
PCG, PCL, G3	C6706-C1-cds0818	I	VC_1180	AE003852	G4987 573 ABC-type transport system involved in cytochrome bd biosynthesis, fused ATPase and permease components	
PCG, PCL, G3	C6706-C1-cds0819	I	VC_1181	AE003852	OG4988 559 ABC-type transport system involved in cytochrome bd biosynthesis, ATPase and permease components	
PCG, PCL, G3	C6706-C1-cds0840	I	VC_1203	AE003852	HutU COG2987 561 Urocanate hydratase	
PCG, PCL, G3	C6706-C1-cds0841	I	VC_1204	AE003852	SpeB COG0010 305 Arginase/agmatinase/formimionoglutamate hydrolase, arginase family	
PCG, PCL, G3	C6706-C1-cds0860	I	VC_1225	AE003852	-	
PCG, PCL, G3	C6706-C1-cds0951	I	VC_1322	AE003852	COG2991 COG2991 77 Uncharacterized protein conserved in bacteria	
PCG, PCL, G3	C6706-C1-cds0952	I	VC_1323	AE003852	COG1433 COG1433 121 Uncharacterized conserved protein	
PCG, PCL, G3	C6706-C1-cds1089	I	-	AE003852	COG1433 COG1433 121 Uncharacterized conserved protein	
PCG, PCL, G3	C6706-C1-cds1192	I	VC_1514	AE003852	-	
PCG, PCL, G3	C6706-C1-cds1196	I	VC_1518	AE003852	-	
PCG, PCL, G3	C6706-C1-cds1203	I	VC_1525	AE003852	CbiO COG1122 235 ABC-type cobalt transport system, ATPase component	
PCG, PCL, G3	C6706-C1-cds1204	I	VC_1526	AE003852	MobA COG0746 192 Molybdopterin-guanine dinucleotide biosynthesis protein A	
PCG, PCL, G3	C6706-C1-cds1216	I	-	AE003852	-	
PCG, PCL, G3	C6706-C1-cds1252	I	VC_1578	AE003852	AcpP COG0236 80 Acyl carrier protein	
PCG, PCL, G3	C6706-C1-cds1288	I	VC_1613	AE003852	-	

PCG, PCL, G3	C6706-C1-cds1880	I	VC_2240	AE003852	COG3479 COG3479 175 Phenolic acid decarboxylase	
PCG, PCL, G3	C6706-C1-cds1898	I	VC_2259	AE003852	Tsf COG0264 296 Translation elongation factor Ts	
PCG, PCL, G3	C6706-C1-cds1941	I	VC_2305	AE003852	Tsx COG3248 284 Nucleoside-binding outer membrane protein	
PCG, PCL, G3	C6706-C1-cds2045	I	VC_2423	AE003852	PilA COG4969 125 Tfp pilus assembly protein, major pilin PilA	
PCG, PCL, G3	C6706-C1-cds2053	I	VC_2431	AE003852	GyrB COG0187 635 Type IIA topoisomerase (DNA gyrase/topo II, topoisomerase IV), B subunit	
PCG, PCL, G3	C6706-C1-cds2206	I	VC_2593	AE003852	RplB COG0090 275 Ribosomal protein L2	
PCG, PCL, G3	C6706-C1-cds2349	I	VC_2745	AE003852	-	
PCG, PCL, G3	C6706-C1-cds2407	I	VC_0097	AE003852	FliL COG1580 159 Flagellar basal body-associated protein	
PCG, PCL, G3	C6706-C1-cds2432	I	VC_0070	AE003852	-	
PCG, PCL, G3	C6706-C1-cds2493	I	VC_0007	AE003852	RpmH COG0230 44 Ribosomal protein L34	
PCG, PCL, G3	C6706-C1-cds2497	I	VC_0002	AE003852	FldA COG0716 151 Flavodoxins	
PCG, PCL, G3	C6706-C1-cds2518	I	VC_2755	AE003852	HemG COG4635 175 Flavodoxin	
PCG, PCL, G3	C6706-C2-cds0044	II	-	AE003853	BaeS COG0642 336 Signal transduction histidine kinase	
PCG, PCL, G3	C6706-C2-cds0045	II	VC_A1105	AE003853	COG0745 229 Response regulators consisting of a CheY-like receiver domain and a winged-helix DNA-binding domain	
PCG, PCL, G3	C6706-C2-cds102	II	VC_A0056	AE003853	-	
PCG, PCL, G3	C6706-C2-cds209	II	VC_A0177	AE003853	-	
PCG, PCL, G3	C6706-C2-cds322	II	VC_A0303	AE003853	-	Superintegron
PCG, PCL, G3	C6706-C2-cds334	II	VC_A0482	AE003853	COG4453 COG4453 95 Uncharacterized protein conserved in bacteria	Superintegron
PCG, PCL, G3	C6706-C2-cds391	II	VC_A0395	AE003853	COG0384 COG0384 291 Predicted epimerase, PhzC/PhzF homolog	Superintegron
PCG, PCL, G3	C6706-C2-cds392	II	VC_A0396	AE003853	-	Superintegron
PCG, PCL, G3	C6706-C2-cds397	II	VC_A0476	AE003853	GloA COG0346 138 Lactoylglutathione lyase and related lyases	Superintegron
PCG, PCL, G3	C6706-C2-cds412	II	VC_A0422	AE003853	StbD COG2161 86 Antitoxin of toxin-antitoxin stability system	Superintegron
PCG, PCL, G3	C6706-C2-cds418	II	VC_A0432	AE003853	GloA COG0346 138 Lactoylglutathione lyase and related lyases	Superintegron
PCG, PCL, G3	C6706-C2-cds433	II	VC_A0451	AE003853	-	Superintegron
PCG, PCL, G3	C6706-C2-cds434	II	-	AE003853	-	Superintegron
PCG, PCL, G3	C6706-C2-cds450	II	VC_A0469	AE003853	COG2944 COG2944 104 Predicted transcriptional regulator	Superintegron
PCG, PCL, G3	C6706-C2-cds458	II	VC_A0477	AE003853	StbD COG2161 86 Antitoxin of toxin-antitoxin stability system	Superintegron
PCG, PCL, G3	C6706-C2-cds528	II	VC_A0554	AE003853	-	
PCG, PCL, G3	C6706-C2-cds580	II	VC_A0609	AE003853	-	
PCG, PCL, G3	C6706-C2-cds640	II	VC_A0677	AE003853	NapD COG3062 94 Uncharacterized protein involved in formation of periplasmic nitrate reductase	
PCG, PCL, G3	C6706-C2-cds682	II	VC_A0721	AE003853	-	
PCG, PCL, G3	C6706-C2-cds685	II	VC_A0724	AE003853	ModC COG4148 352 ABC-type molybdate transport system, ATPase component	
PCG, PCL, G3	C6706-C2-cds686	II	VC_A0724]	AE003853	ModC COG4149 225 ABC-type molybdate transport system, permease component	
PCG, PCL, G3	C6706-C2-cds740	II	VC_A0782	AE003853	1 COG4181 228 Predicted ABC-type transport system involved in lysophospholipase L1 biosynthesis, ATPase component	
PCG, PCL, G3	C6706-C2-cds750	II	VC_A0793	AE003853	-	
PCG, PCL, G3	C6706-C2-cds775	II	VC_A0821	AE003853	-	
PCG, PCL, G3	C6706-C2-cds794	II	VC_A0845	AE003853	-	
PCG, PCL, G3	C6706-C2-cds837	II	VC_A0893	AE003853	CybC COG3783 100 Soluble cytochrome b562	

Table S4. List of strains used in this study

Strain	Description	Source
V. cholerae C6706	Wild-type, clinical *V. cholerae* O1 El Tor strain C6706; Sm^R	Lab collection
V. cholerae 2010EL-1786	Wild type, clinical *V. cholerae* O1 El Tor strain from the outbreak in Haiti	ATCC
V. cholerae O395	Wild type, clinical *V. cholerae* Classical strain	Lab collection
E. coli S17 pir	Host for cloning vector	Lab collection
C6706Δ*toxR*	*toxR* gene deleted in the El Tor C6706 strain	This study
C6706Δ*lacZ*	*lacZ* gene deleted in the El Tor C6706 strain	This study
ompU^{GBE1114}	*V. cholerae* C6706 encoding *ompU* allele from environmental strain *V. cholerae* GBE1114	Shapiro et al, 2017
rfbT^{A325}	*V. cholerae* C6706 encoding *rfbT* allele from environmental strain *V. cholerae* A325	This study
tcpA^{IRLE0081}	*V. cholerae* C6706 encoding *tcpA* allele from environmental strain *V. cholerae* IRLE0081	This study
toxT^{IRLE0081}	*V. cholerae* C6706 encoding *toxT* allele from environmental strain *V. cholerae* IRLE0081	This study
tcpF^{IRLE0081}	*V. cholerae* C6706 encoding *tcpF* allele from environmental strain *V. cholerae* IRLE0081	This study
tcpF$^{NIHE\_58}$	*V. cholerae* C6706 encoding *tcpF* allele from environmental strain *V. cholerae* NIHE_58	This study
hsdS^{Pivert}	*V. cholerae* C6706 encoding *hsdS* allele from environmental strain *V. cholerae* PivertUAT4Aug	This study
VC1791^{Pivert}	*V. cholerae* C6706 encoding tape measure (VC1791) allele from environmental strain *V. cholerae* PivertUAT4Aug	This study
VC0176^{IRLE0062}	*V. cholerae* C6706 encoding VC0176 allele from environmental strain *V. cholerae* IRLE0062	This study
VC0176^{IRLE0062}:: *rfbT*^{A325}	Double mutant; *rfbT*^{A325} environmental allele introduced into the *V. cholerae* C6706 encoding *VC0176*^{IRL0062}	This study
Double mutant:: *VC1791*^{Pivert}	Triple mutant; *VC1791*^{PivertUAT4Aug} environmental allele introduced into the *V. cholerae* C6706 double mutant	This study
Triple mutant::*ompU*^{GBE1114}	Quadruple mutant; *ompU*^{GBE1114} environmental allele introduced into the *V. cholerae* C6706 triple mutant	This study
Quadruple mutant::*toxT*^{IRLE0081}	Quintuple mutant; *toxT*^{IRLE0081} environmental allele introduced into the *V. cholerae* C6706 quadruple mutant	This study
Quintuple mutant::*tcpA*^{IRLE0081}	Sextuple mutant; *tcpA*^{IRLE0081} environmental allele introduced into the *V. cholerae* C6706 quintuple mutant	This study
O395Δ*lacZ*	*lacZ* gene deleted in the Classical O395 strain	This study
O395::*tcpF*^{IRLE0081}	*V. cholerae* O395 encoding *tcpF* allele from environmental strain *V. cholerae* IRLE0081	This study
2010ELΔ*lacZ*	*lacZ* gene deleted in the Haiti isolate 2010EL-1786	This study
2010EL::*tcpF*^{IRLE0081}	*V. cholerae* 2010EL-1786 (Haiti isolate) encoding *tcpF* allele from environmental strain *V. cholerae* IRLE0081	This study
